# Supplementary figures and images for: Ezrin defines TSC complex activation at endosomal compartments through EGFR–AKT signaling
Source: eLife. 2025 Feb 12;13:RP98523. doi: 10.7554/eLife.98523 (PMC11820125; doi:10.7554/eLife.98523)

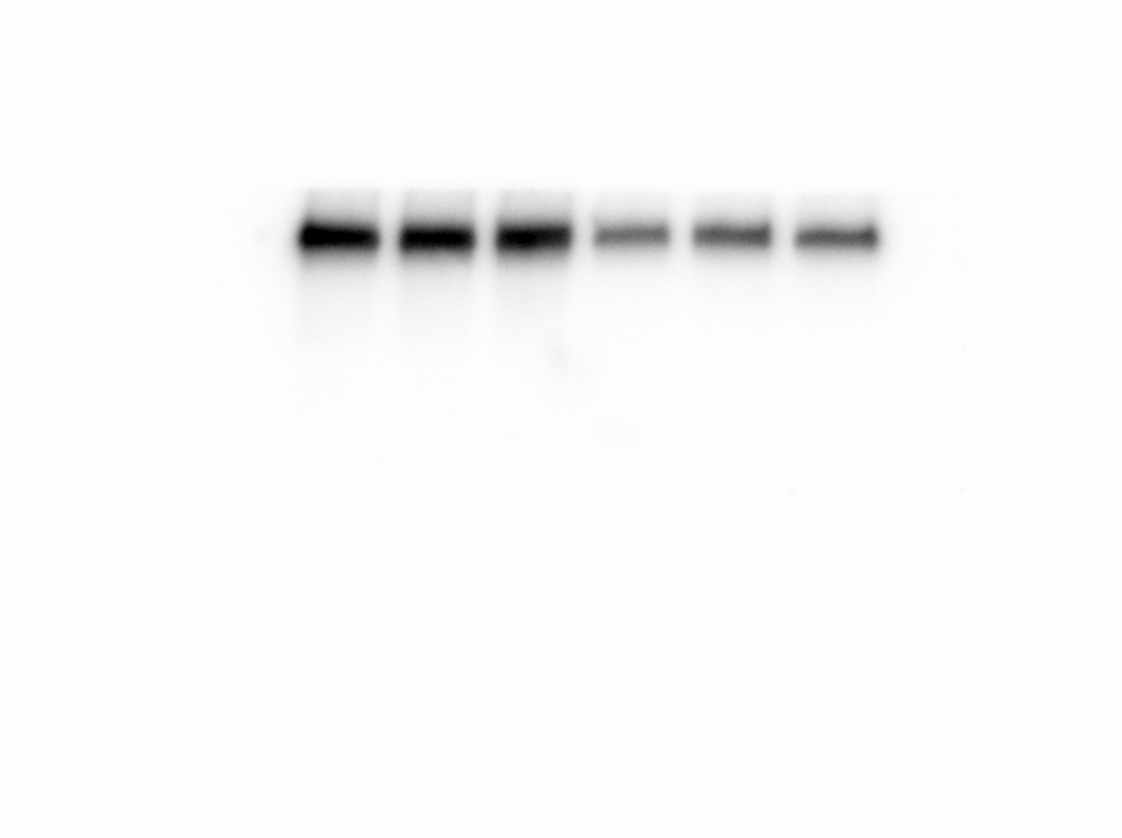

Supplement: Figure 1—source data 1. [file elife-98523-fig1-data1.zip › Figure 1/MEF WT KO P62.tif]

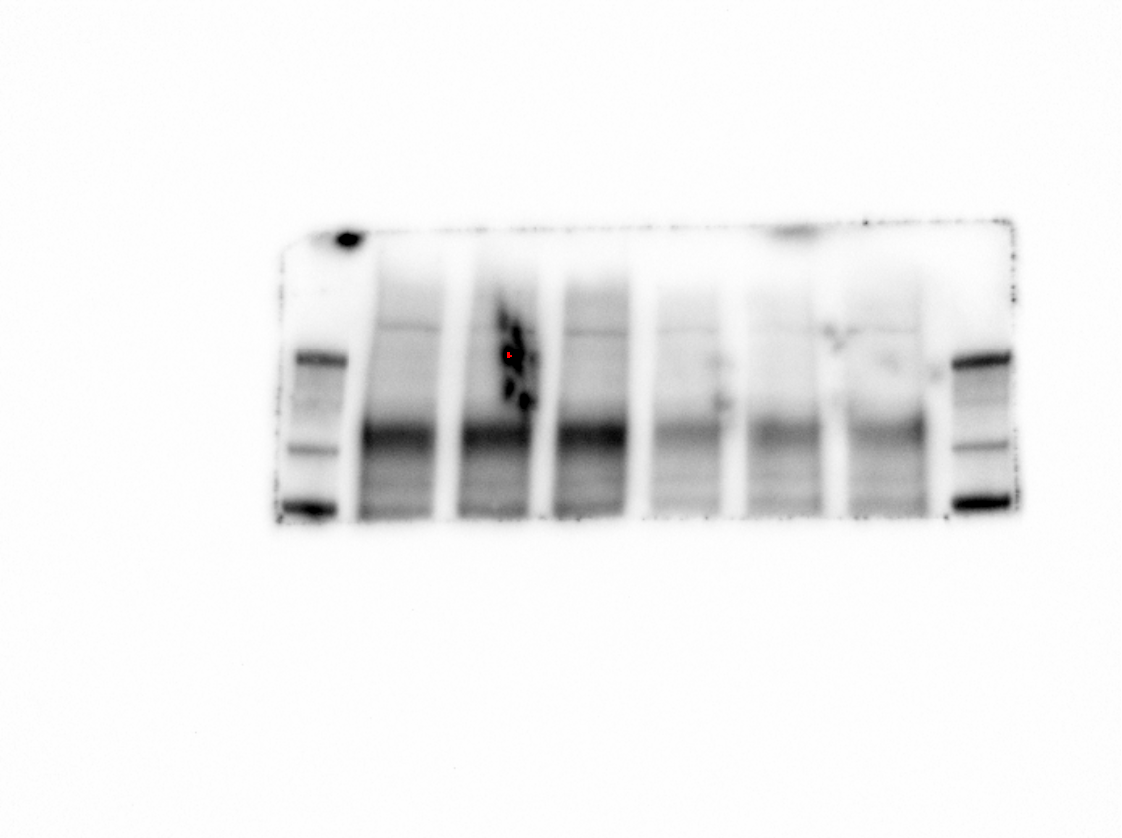

Supplement: Figure 1—source data 1. [file elife-98523-fig1-data1.zip › Figure 1/MEF WT KO NBR1.tif]

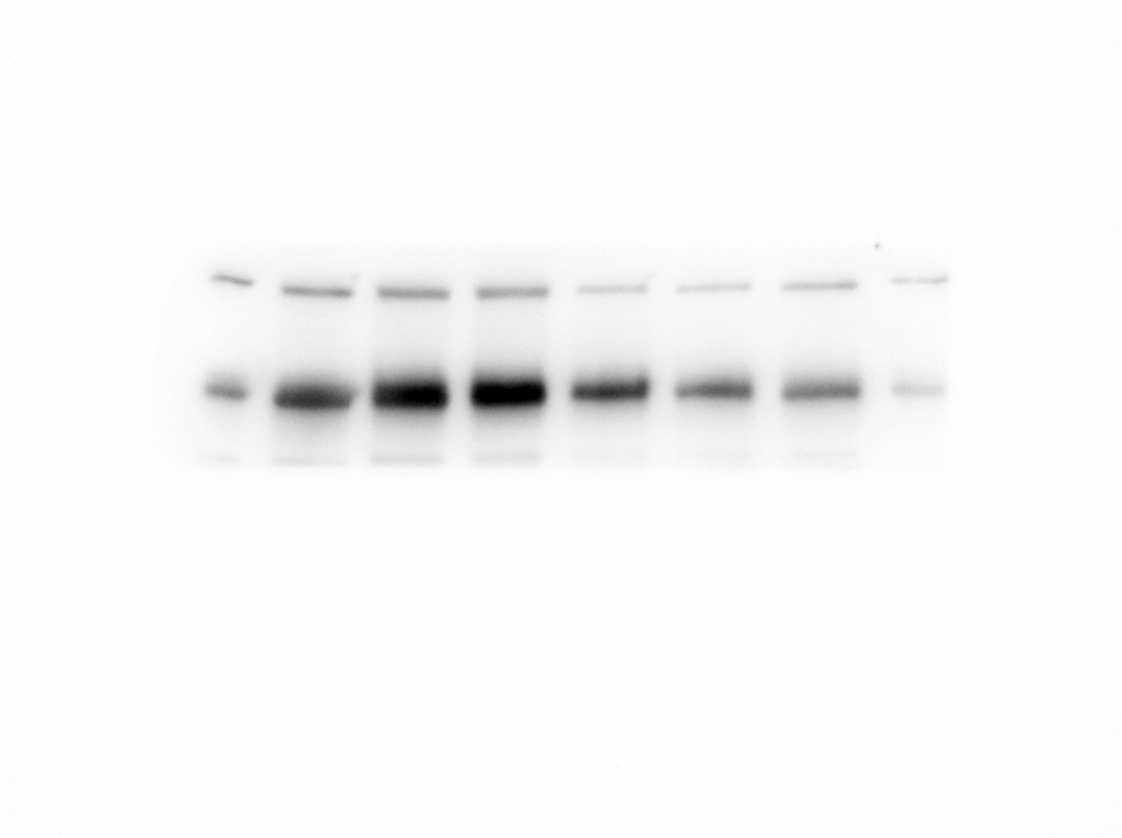

Supplement: Figure 1—source data 1. [file elife-98523-fig1-data1.zip › Figure 1/MEF 2 GEL CATHEPSIN D.tif]

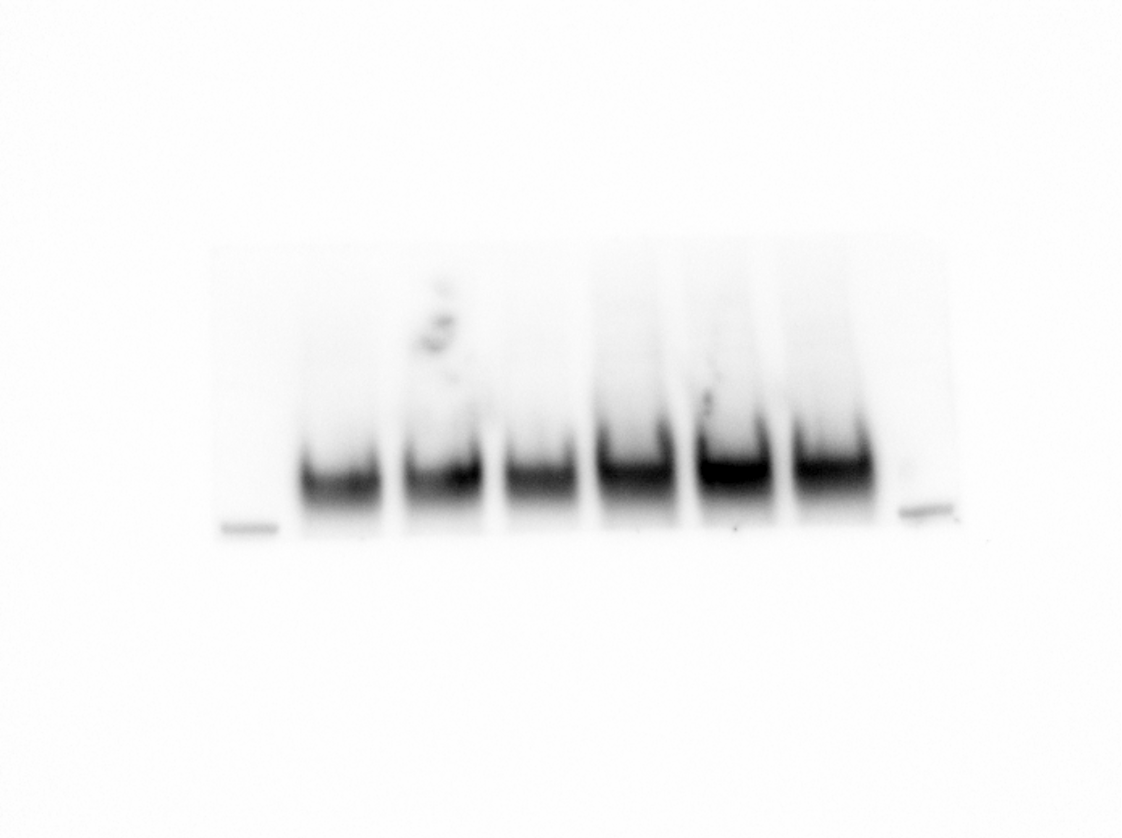

Supplement: Figure 1—source data 1. [file elife-98523-fig1-data1.zip › Figure 1/MEF WT KO LAMP1 .tif]

**Figure 1 h**

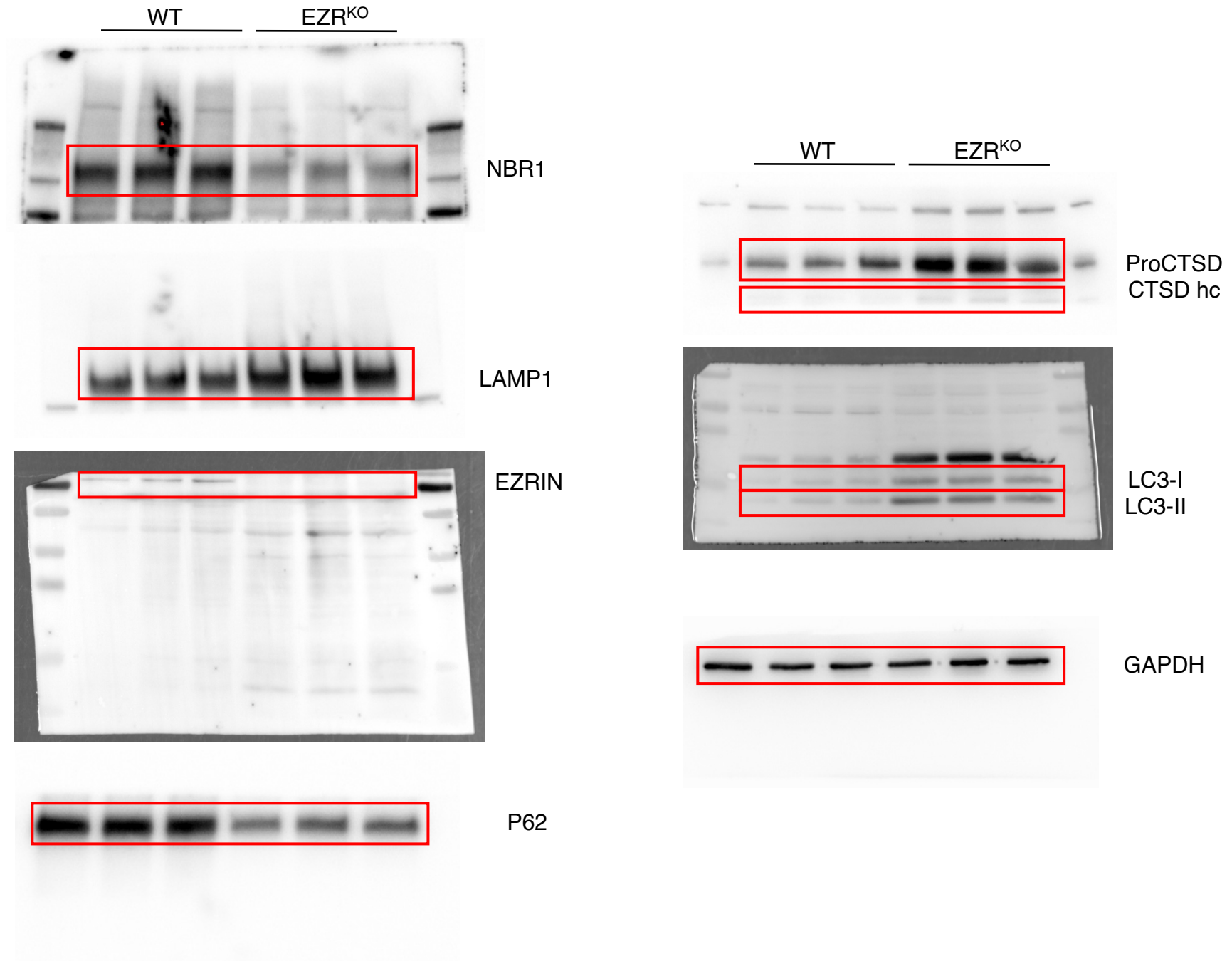

Supplement: Figure 1—source data 1. [file elife-98523-fig1-data1.zip › Figure 1/Figure 1.pdf]

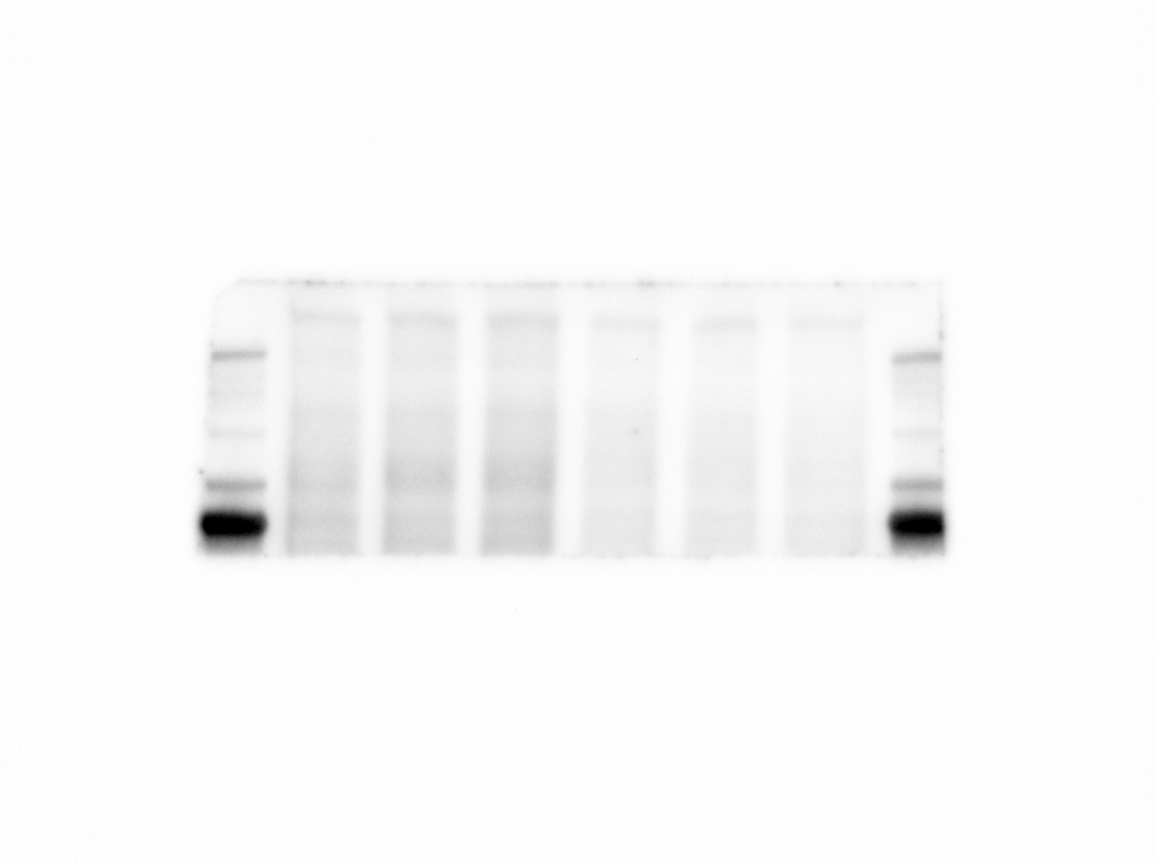

Supplement: Figure 1—figure supplement 1—source data 1. [file elife-98523-fig1-figsupp1-data1.zip › Figure suppl 1/HELA WT KO NBR1.tif]

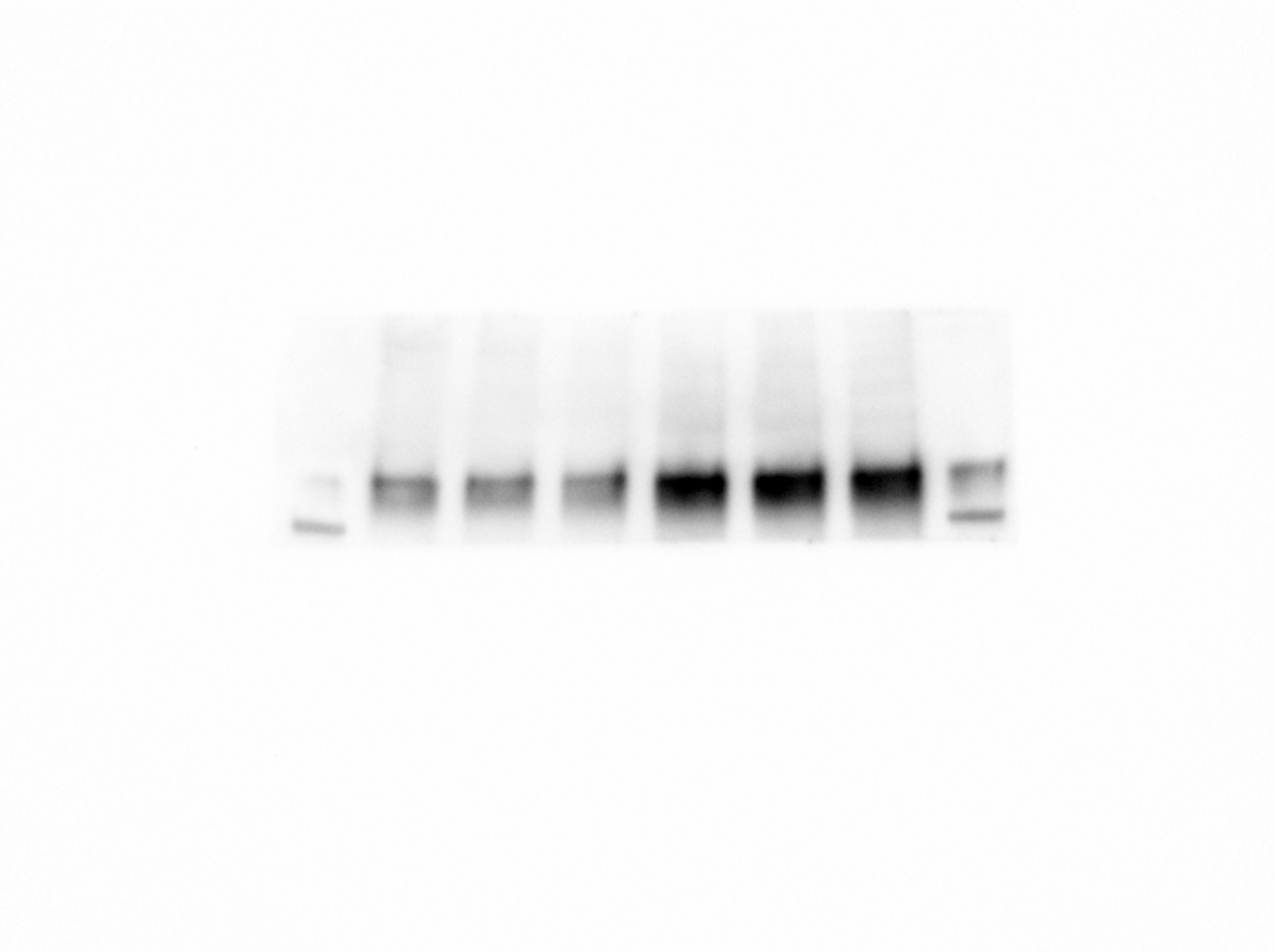

Supplement: Figure 1—figure supplement 1—source data 1. [file elife-98523-fig1-figsupp1-data1.zip › Figure suppl 1/HELA WT KO LAMP1.tif]

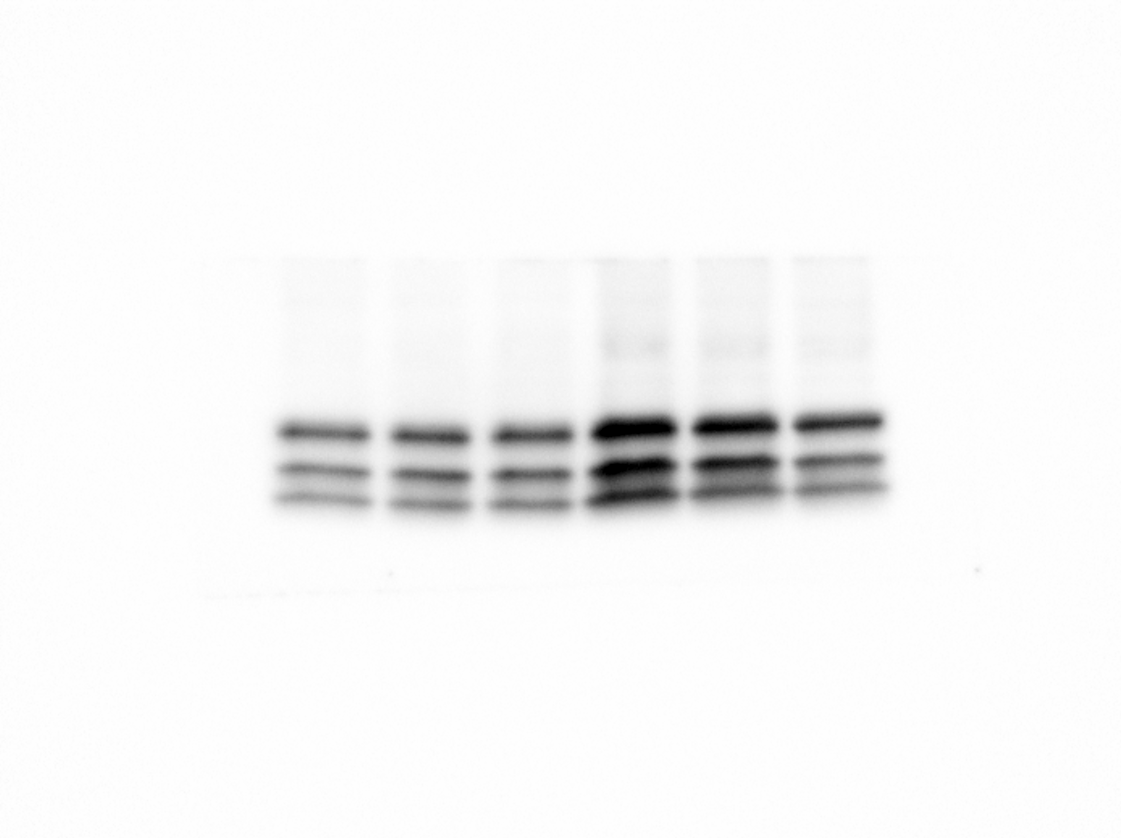

Supplement: Figure 1—figure supplement 1—source data 1. [file elife-98523-fig1-figsupp1-data1.zip › Figure suppl 1/HELA WT KO LC3.tif]

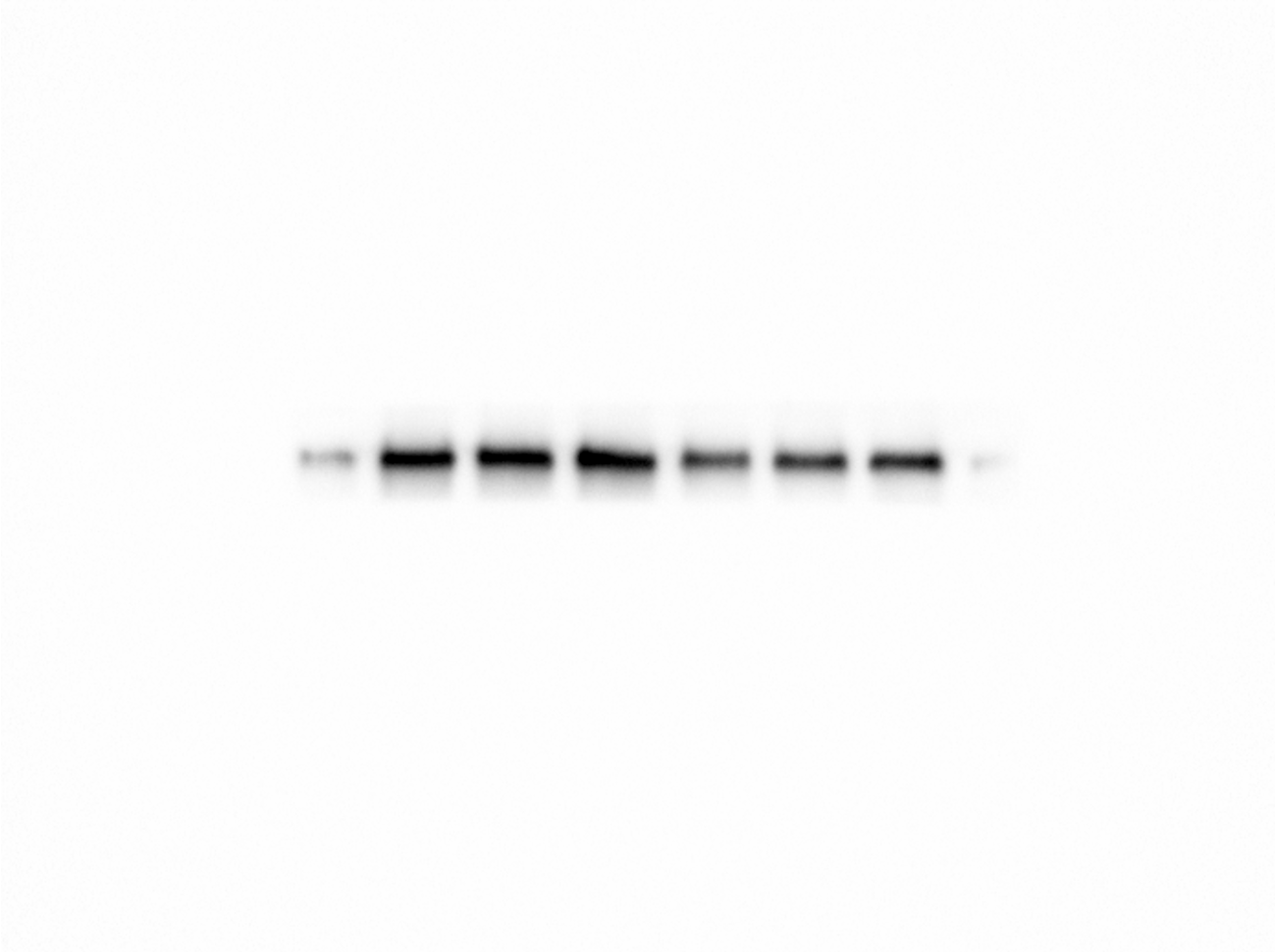

Supplement: Figure 1—figure supplement 1—source data 1. [file elife-98523-fig1-figsupp1-data1.zip › Figure suppl 1/p62 2gel.tif]

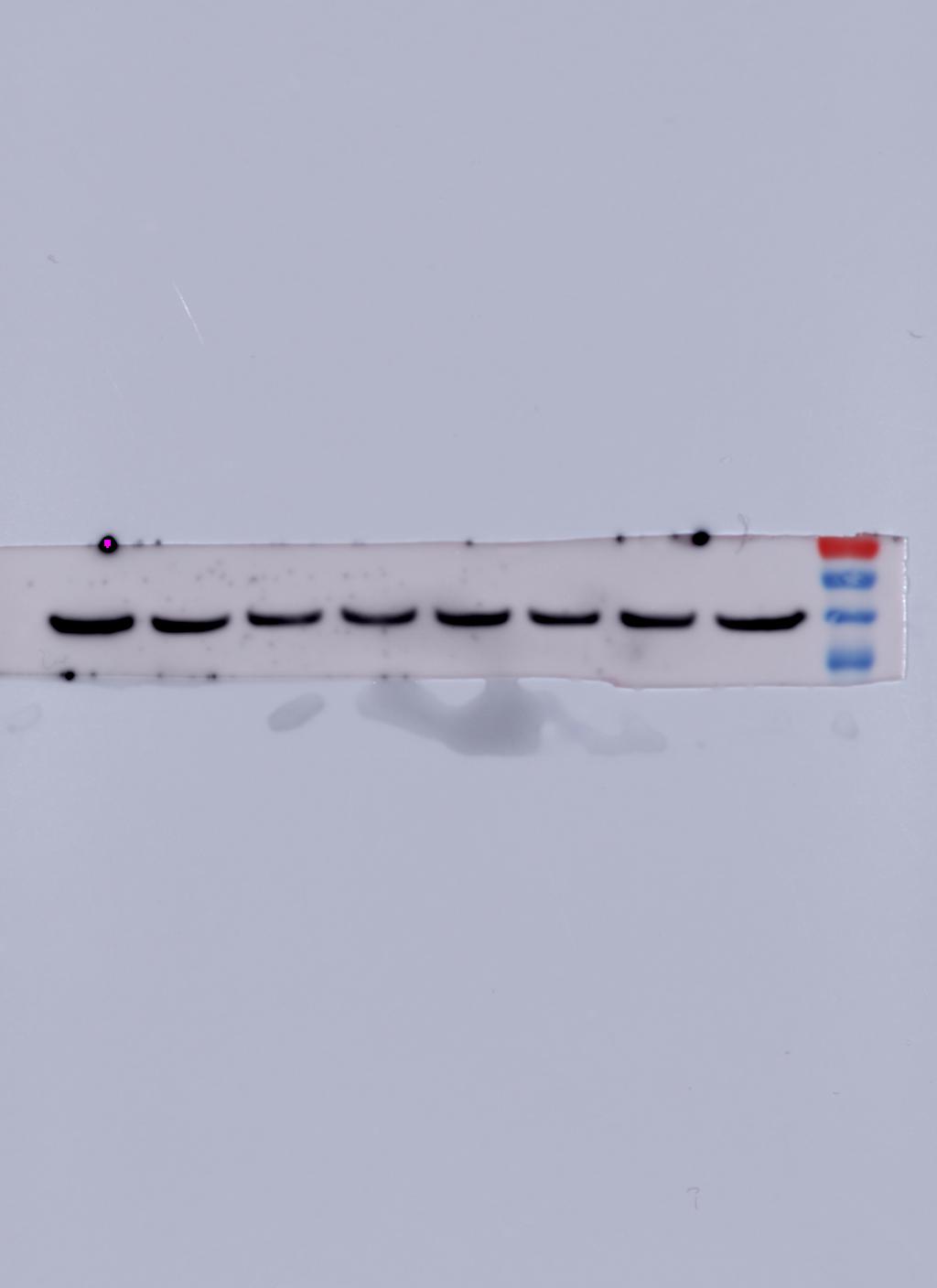

Supplement: Figure 1—figure supplement 1—source data 1. [file elife-98523-fig1-figsupp1-data1.zip › Figure suppl 1/WT KO STV BAF GAPDH .jpg]

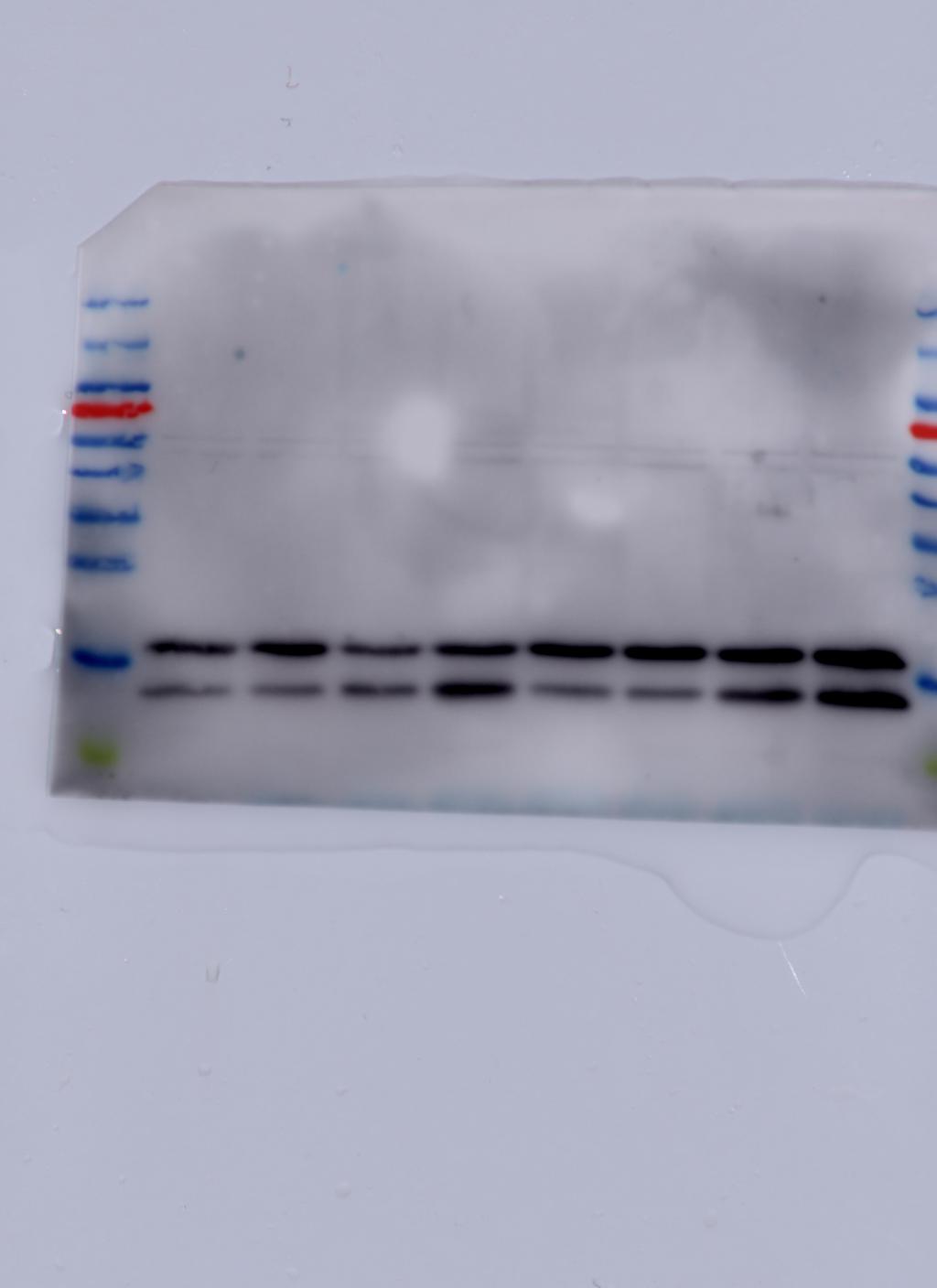

Supplement: Figure 1—figure supplement 1—source data 1. [file elife-98523-fig1-figsupp1-data1.zip › Figure suppl 1/WT KO STV BAF LC3.jpg]

Figure Supplementary 1 h

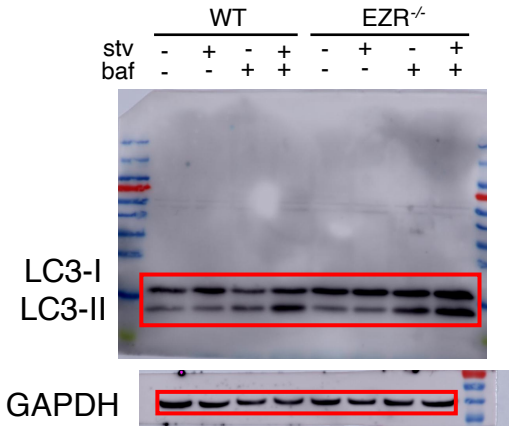

Figure Supplementary 1 i

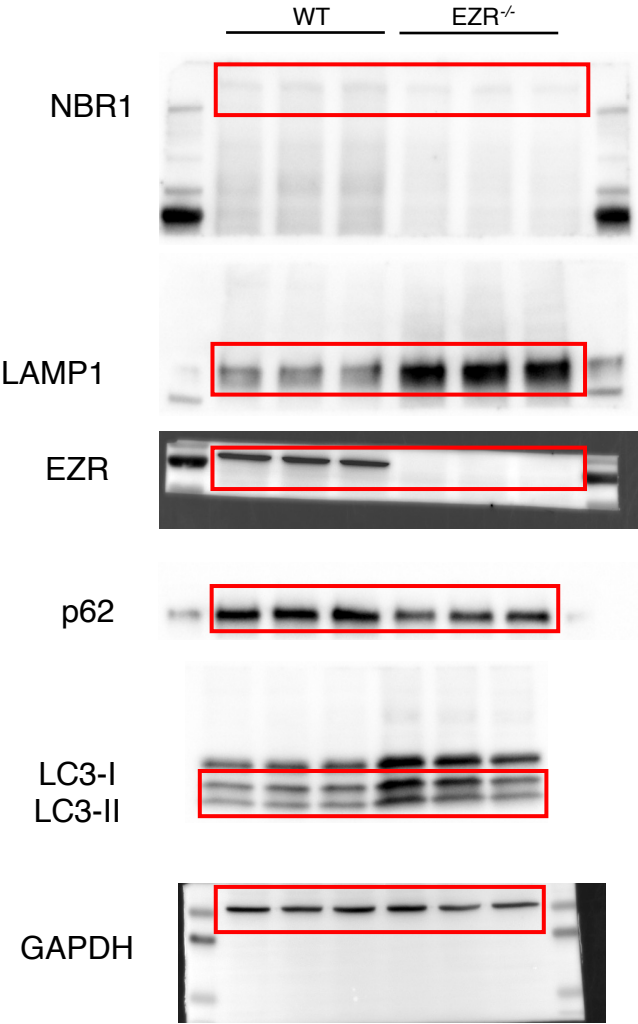

Supplement: Figure 1—figure supplement 1—source data 1. [file elife-98523-fig1-figsupp1-data1.zip › Figure suppl 1/Figure suppl. 1.pdf]

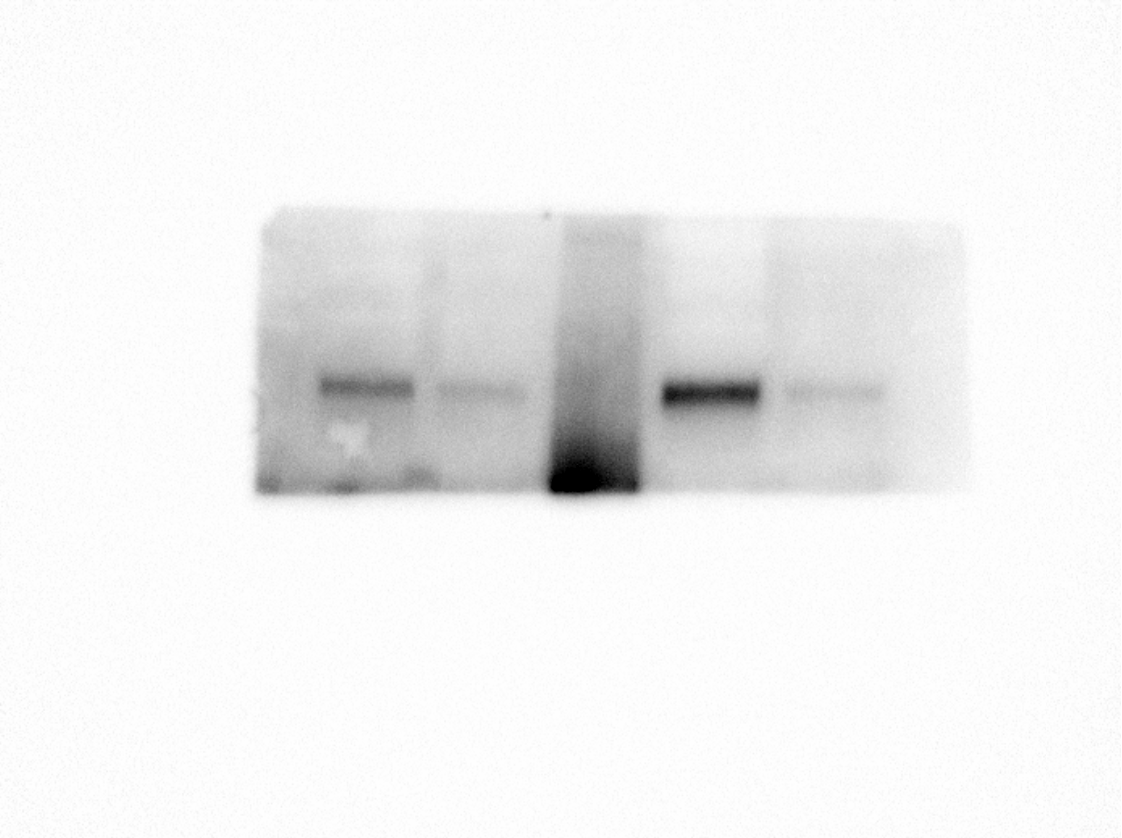

Supplement: Figure 2—source data 1. [file elife-98523-fig2-data1.zip › Figure 2/IP X EZR- WB X EGFR 2.tif]

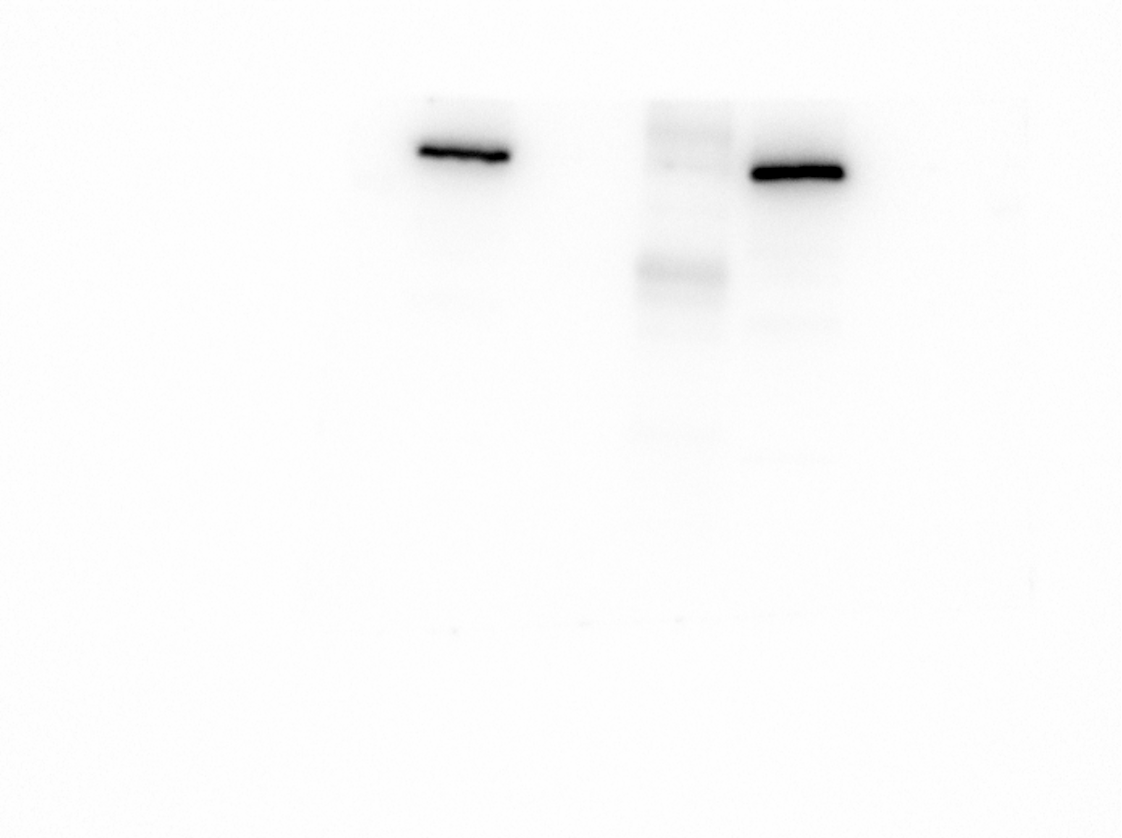

Supplement: Figure 2—source data 1. [file elife-98523-fig2-data1.zip › Figure 2/IP X EZR-WB X EZR 1.tif]

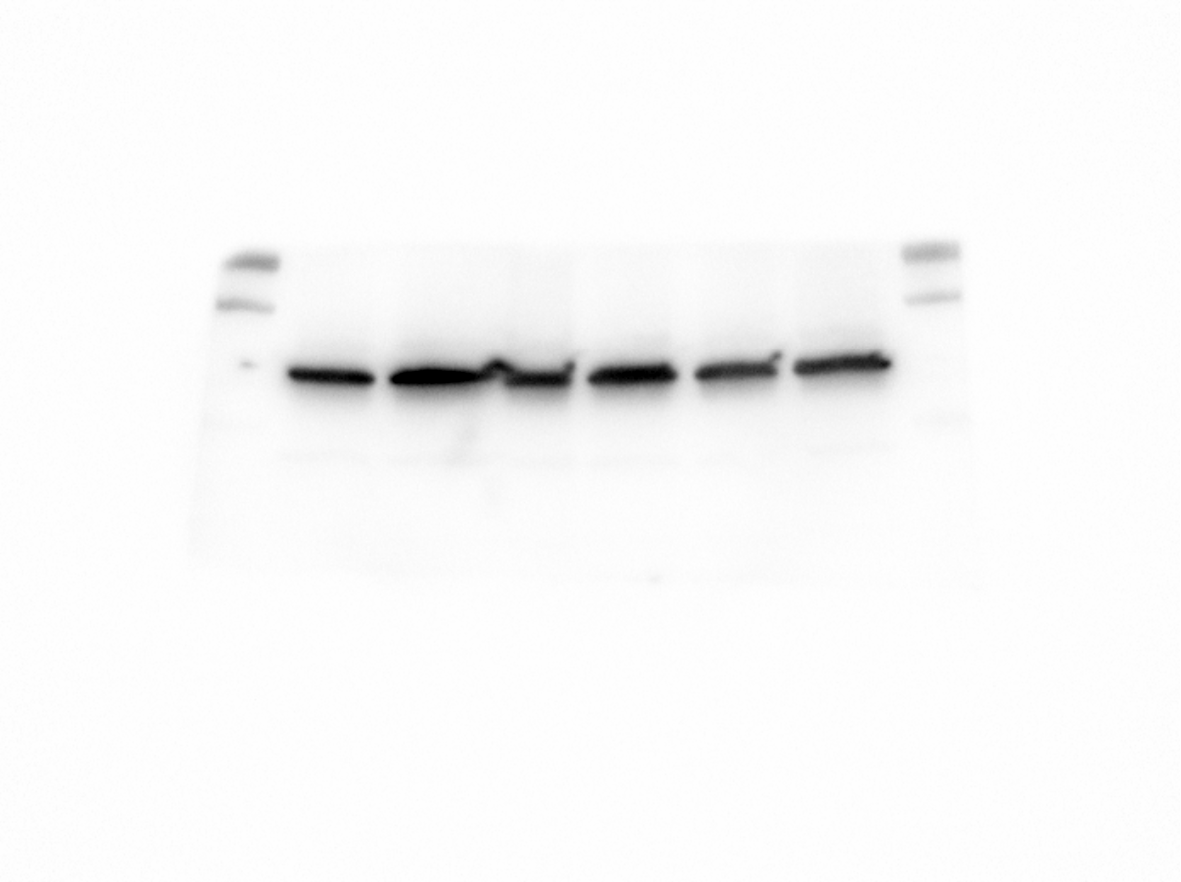

Supplement: Figure 2—source data 1. [file elife-98523-fig2-data1.zip › Figure 2/HELA WT KO GAPDH .tif]

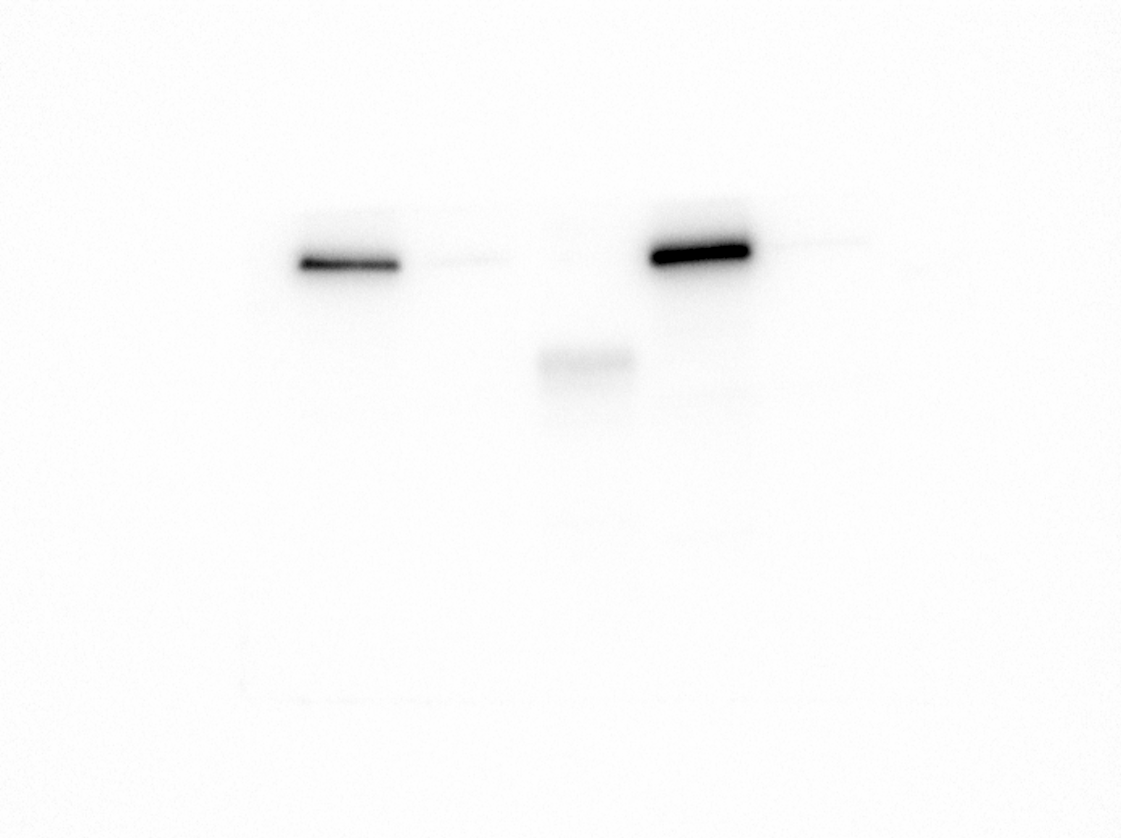

Supplement: Figure 2—source data 1. [file elife-98523-fig2-data1.zip › Figure 2/IP X EZR-WB X EZR 2.tif]

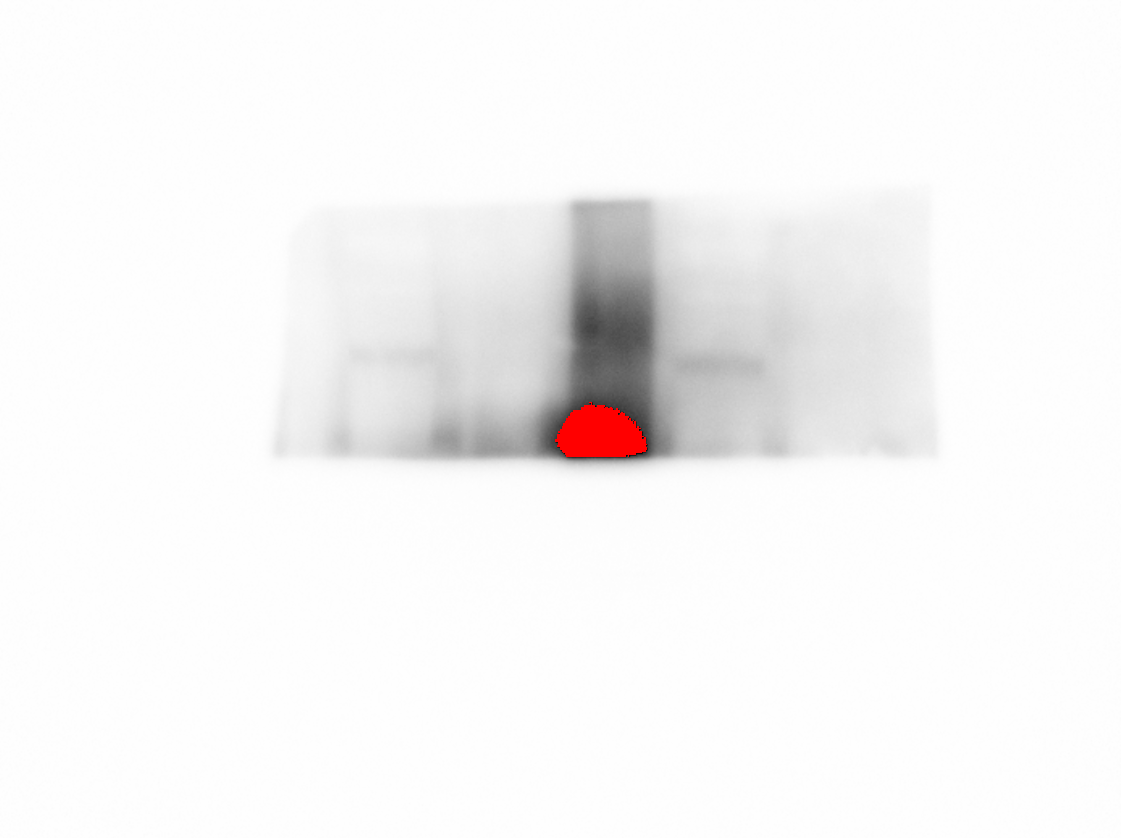

Supplement: Figure 2—source data 1. [file elife-98523-fig2-data1.zip › Figure 2/IP X EZR- WB X EGFR 1.tif]

Figure 2 d

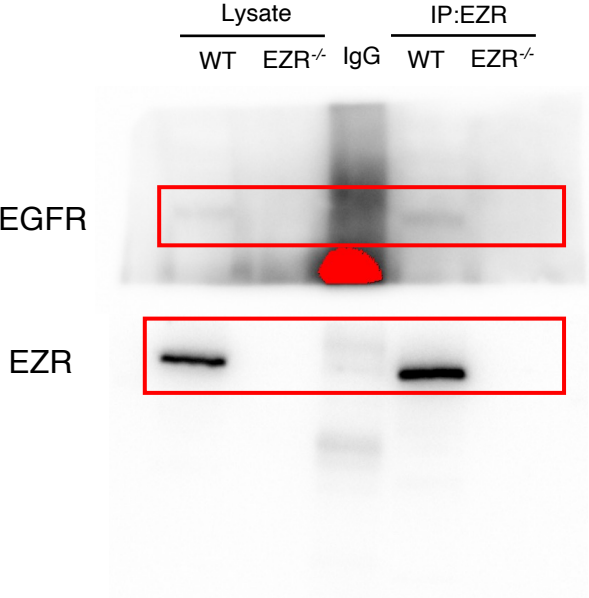

Figure 2 e

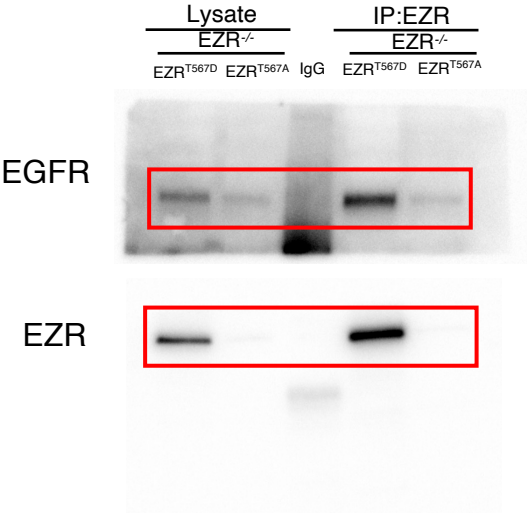

Figure 2 i

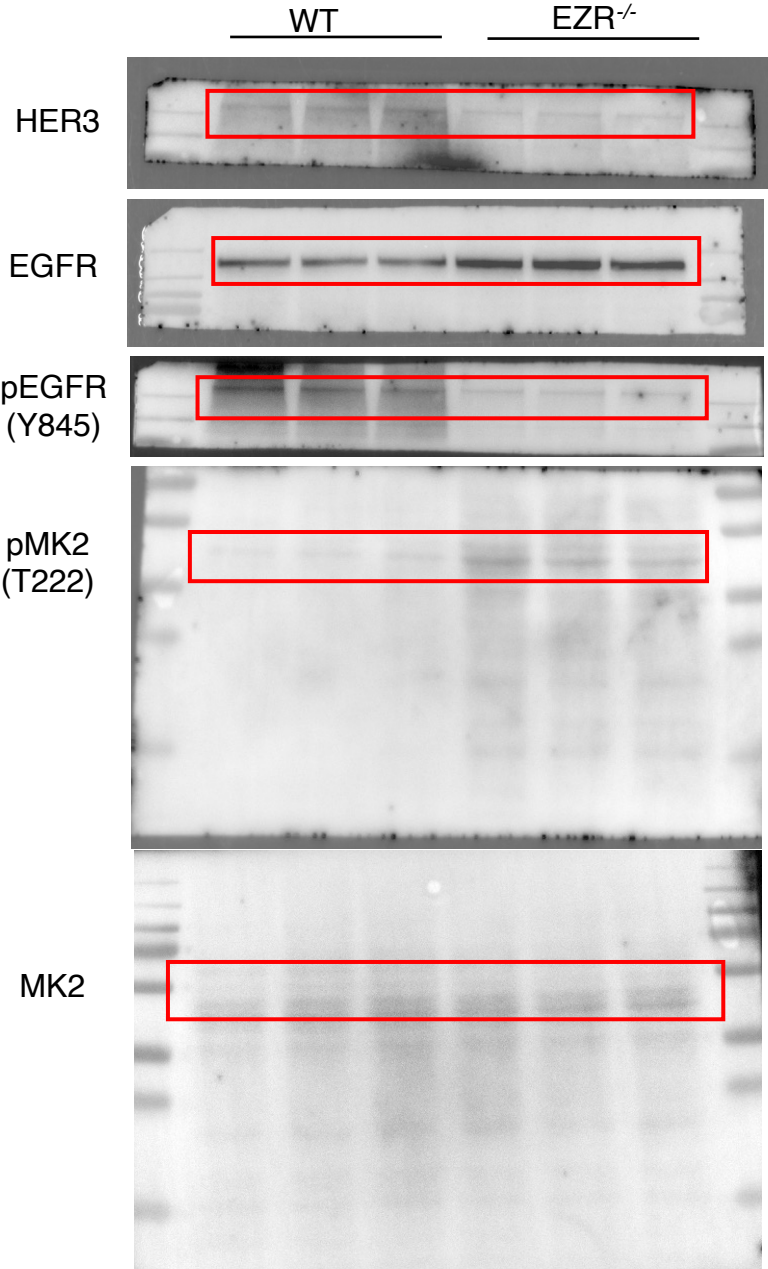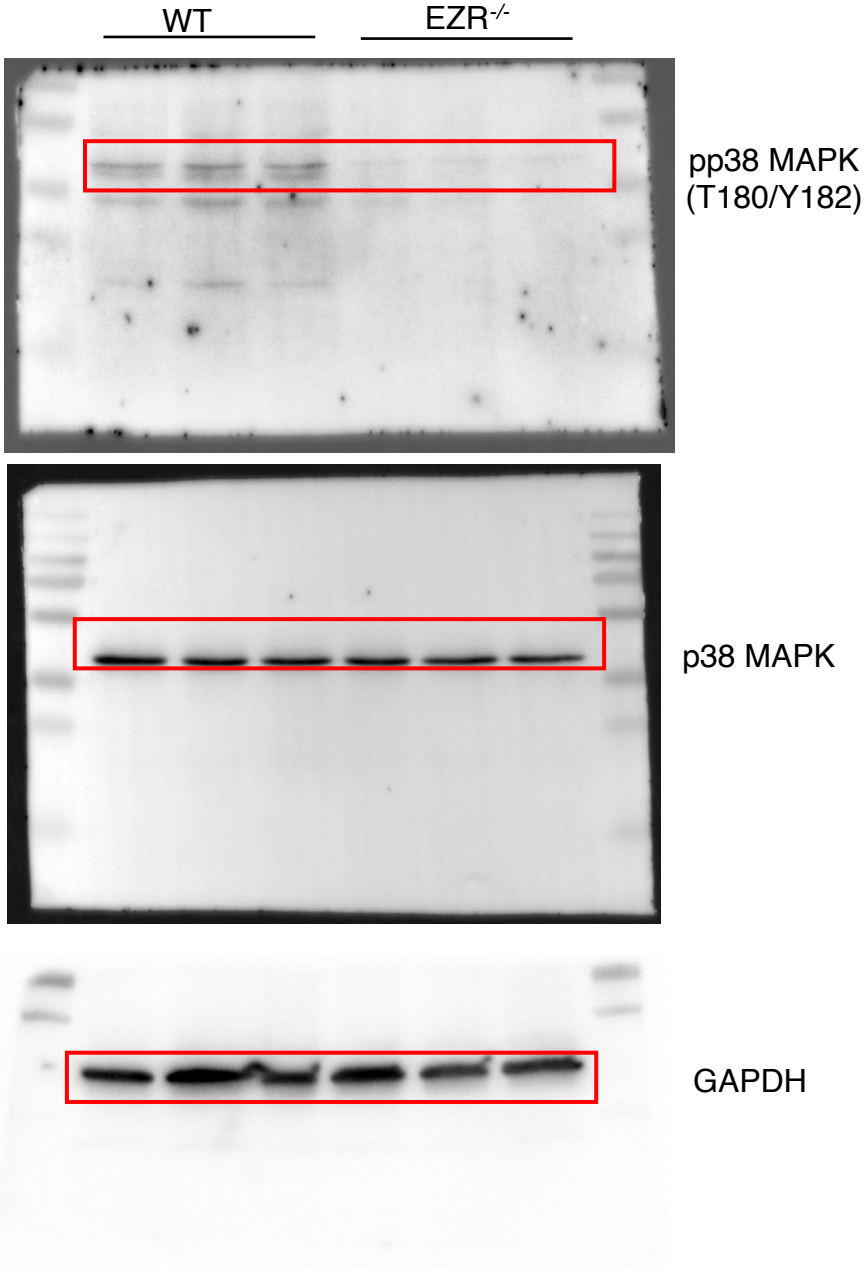

Supplement: Figure 2—source data 1. [file elife-98523-fig2-data1.zip › Figure 2/Figure 2.pdf]

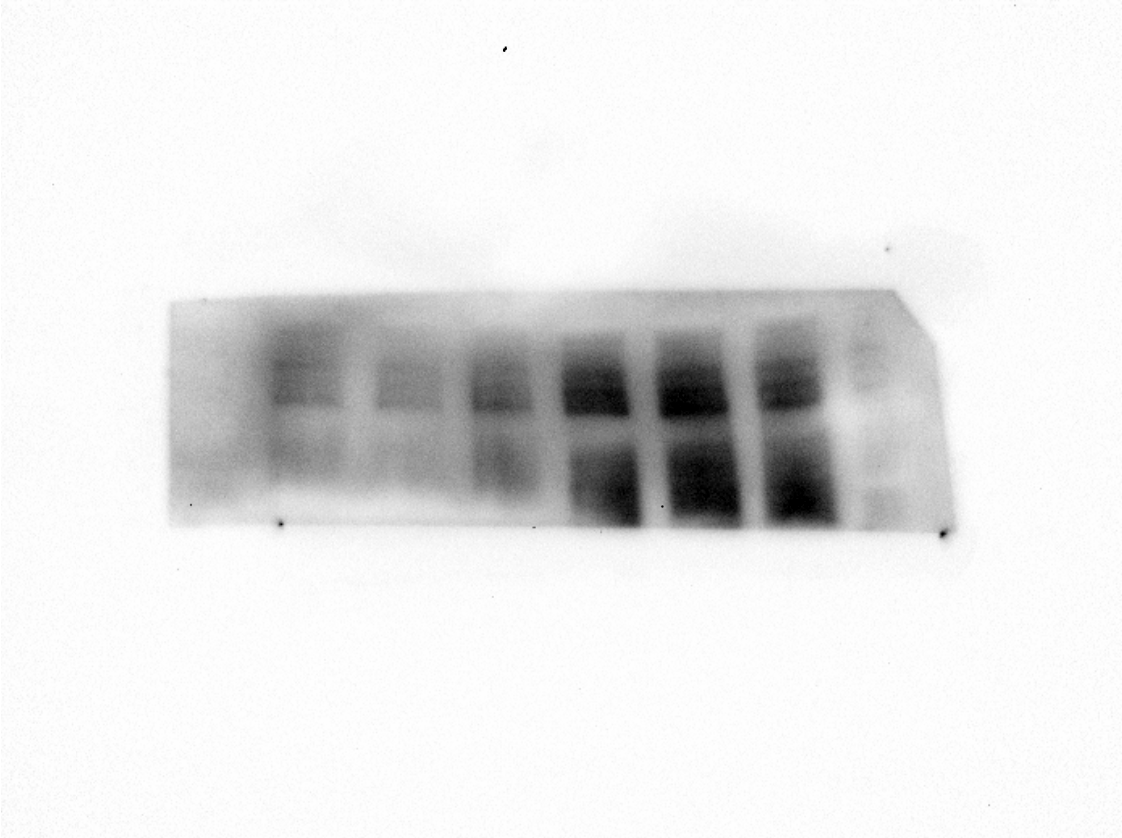

Supplement: Figure 2—figure supplement 1—source data 1. [file elife-98523-fig2-figsupp1-data1.zip › Figure suppl 2/MEF WT KO EGFR.tif]

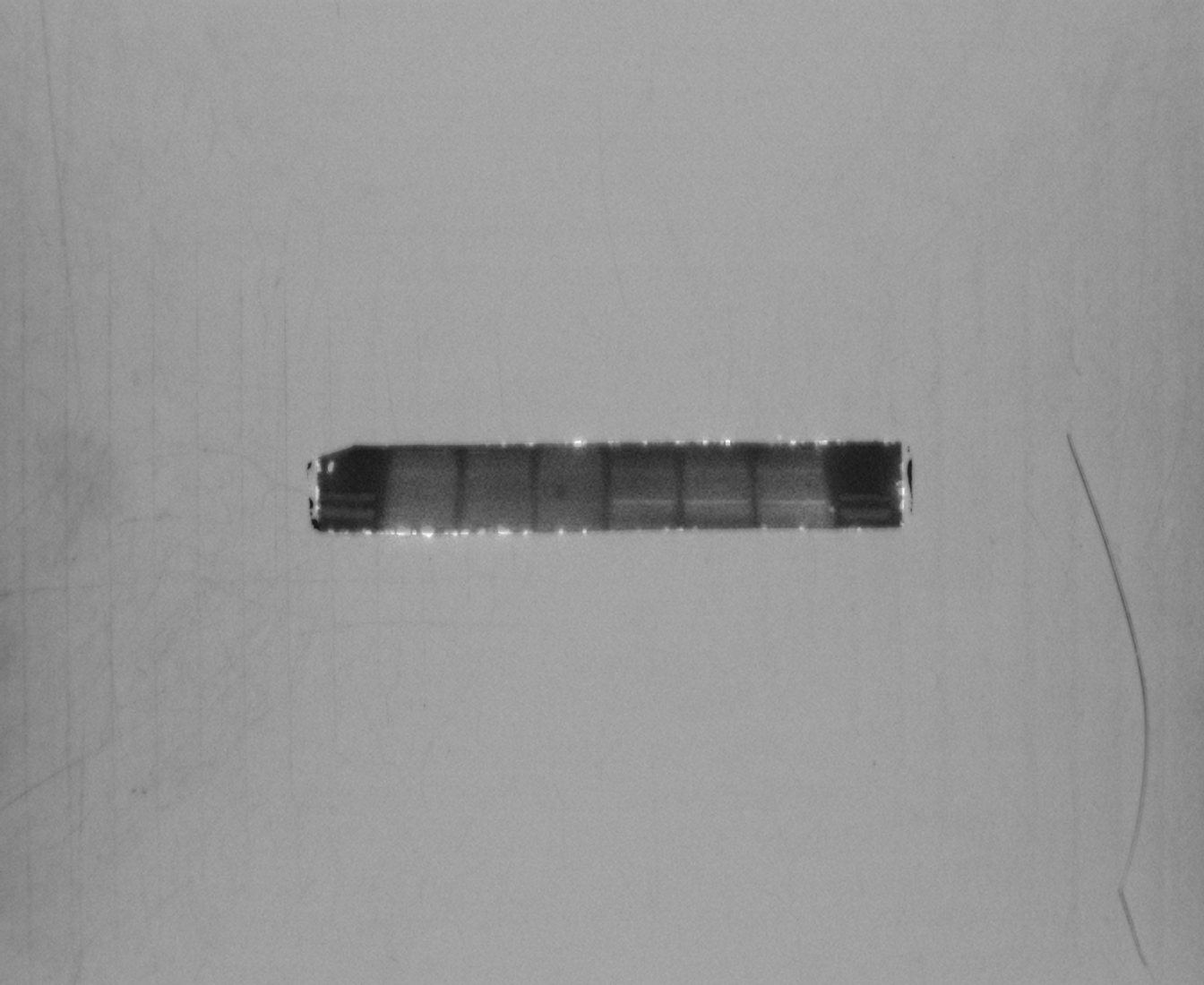

Supplement: Figure 2—figure supplement 1—source data 1. [file elife-98523-fig2-figsupp1-data1.zip › Figure suppl 2/MEF WT KO HER2.Tif]

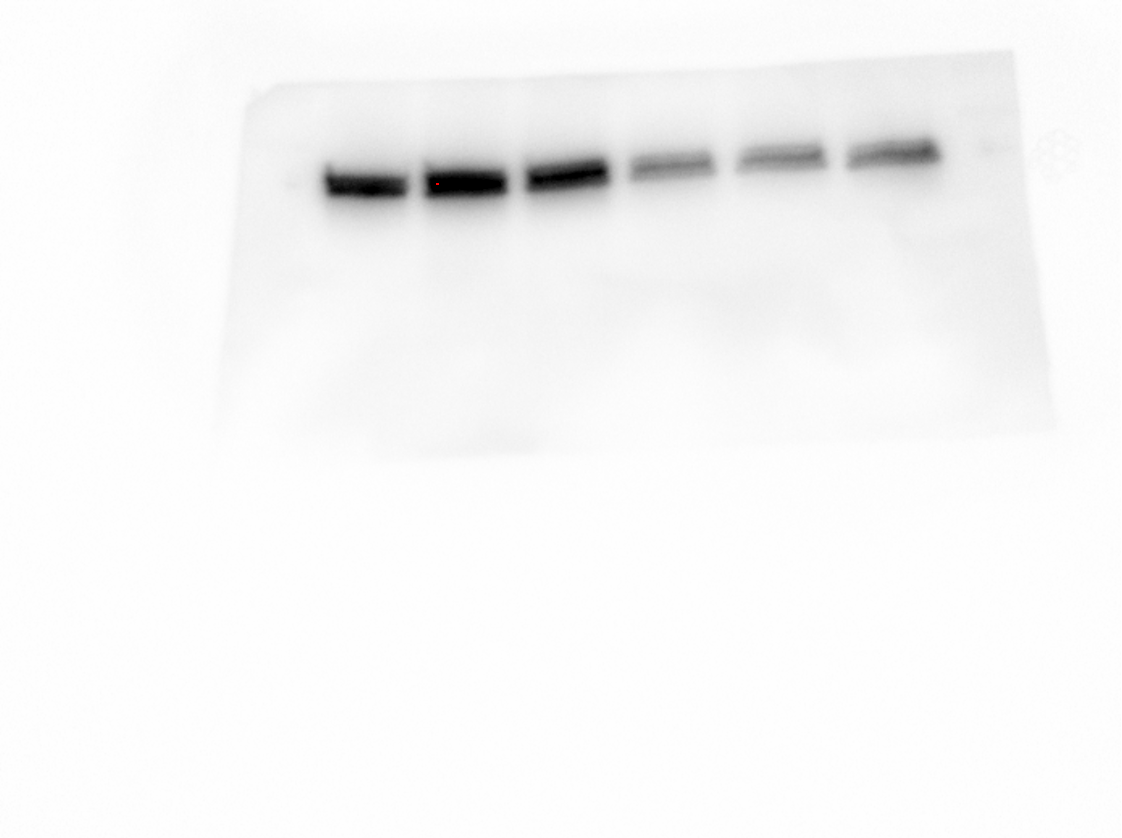

Supplement: Figure 2—figure supplement 1—source data 1. [file elife-98523-fig2-figsupp1-data1.zip › Figure suppl 2/MEF WT KO pP44:42.tif]

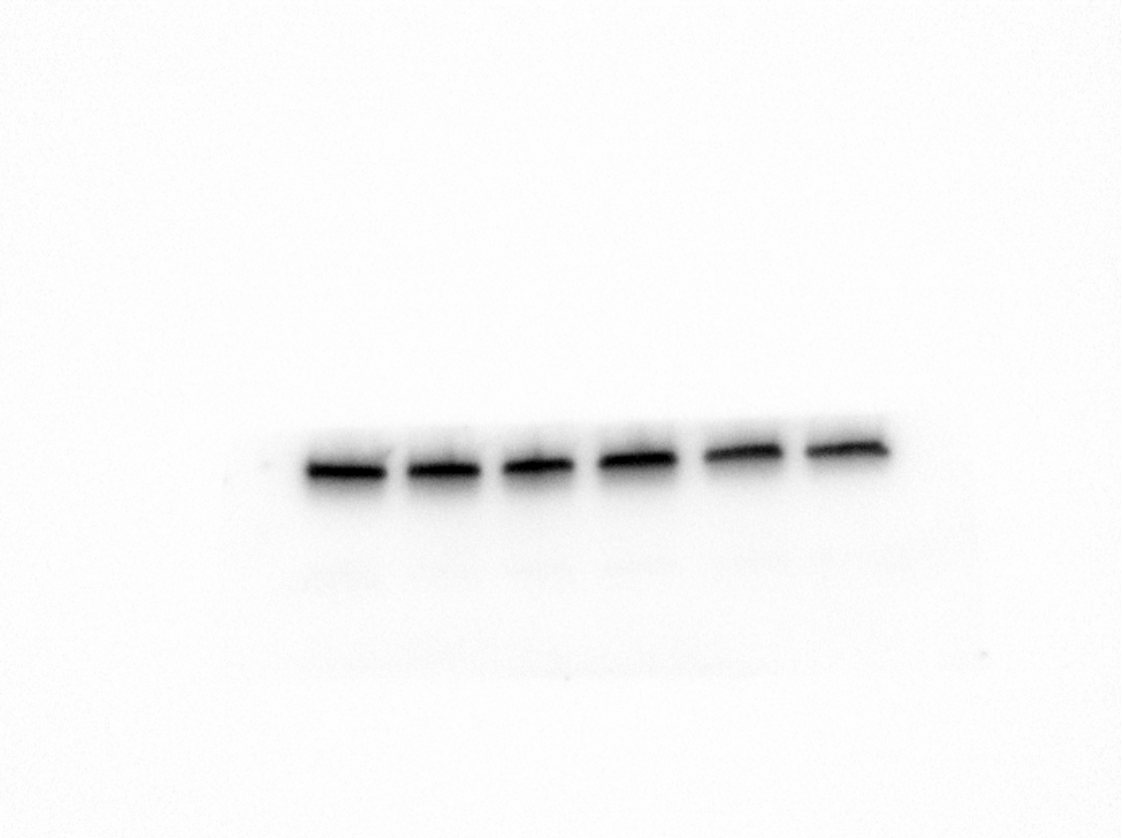

Supplement: Figure 2—figure supplement 1—source data 1. [file elife-98523-fig2-figsupp1-data1.zip › Figure suppl 2/MEF WT KO P38.tif]

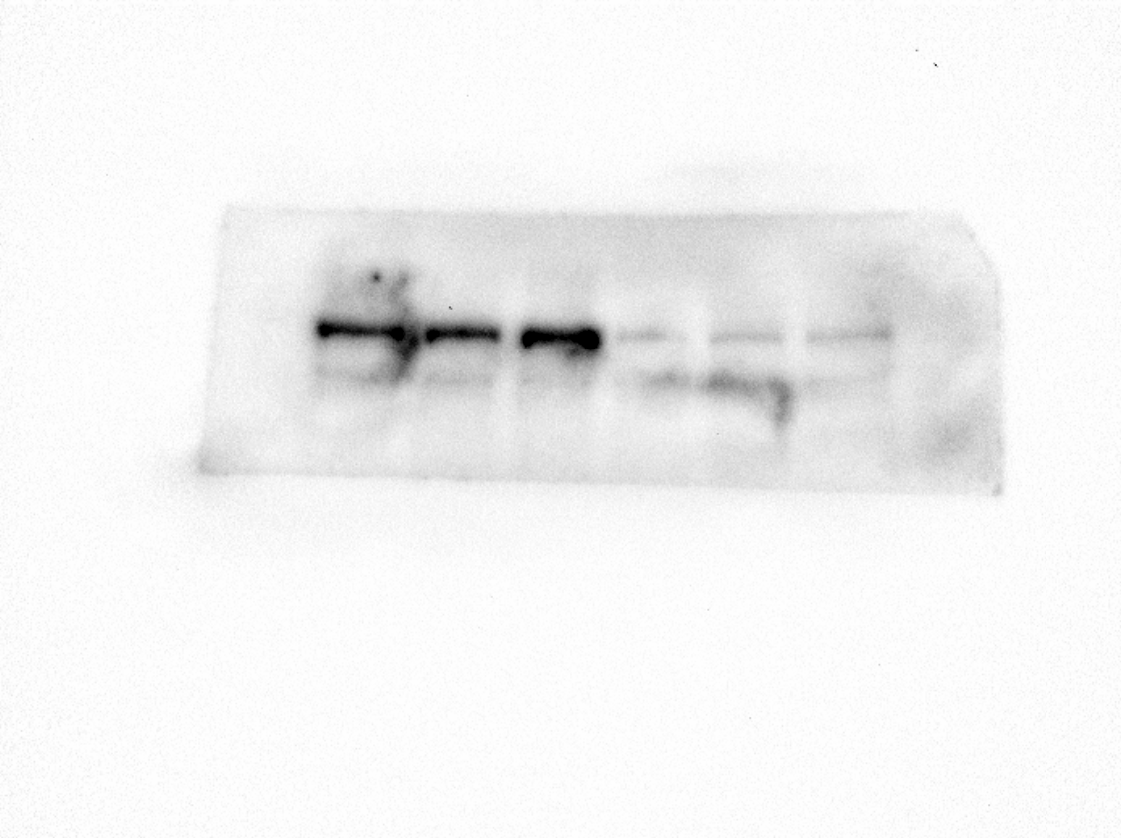

Supplement: Figure 2—figure supplement 1—source data 1. [file elife-98523-fig2-figsupp1-data1.zip › Figure suppl 2/MEF WT KO ENDOSOME EGFR.tif]

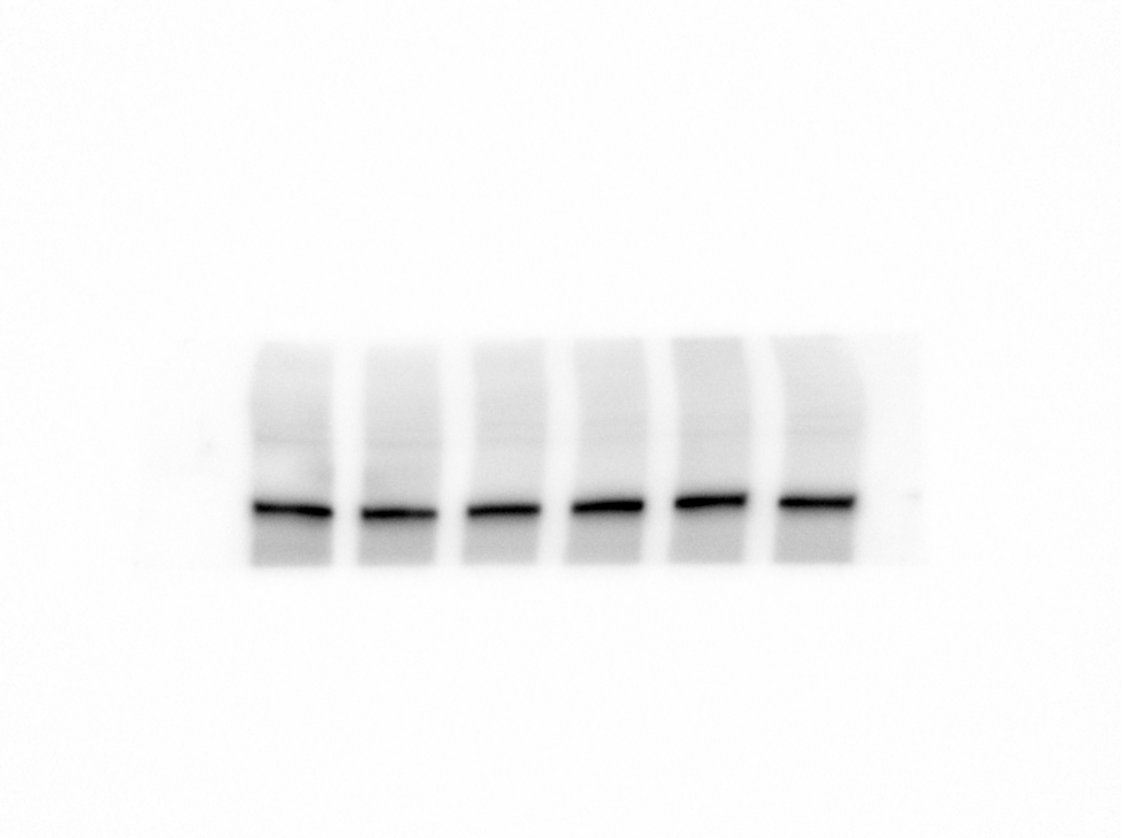

Supplement: Figure 2—figure supplement 1—source data 1. [file elife-98523-fig2-figsupp1-data1.zip › Figure suppl 2/MEF WT KO ENDOSOME EEA1.tif]

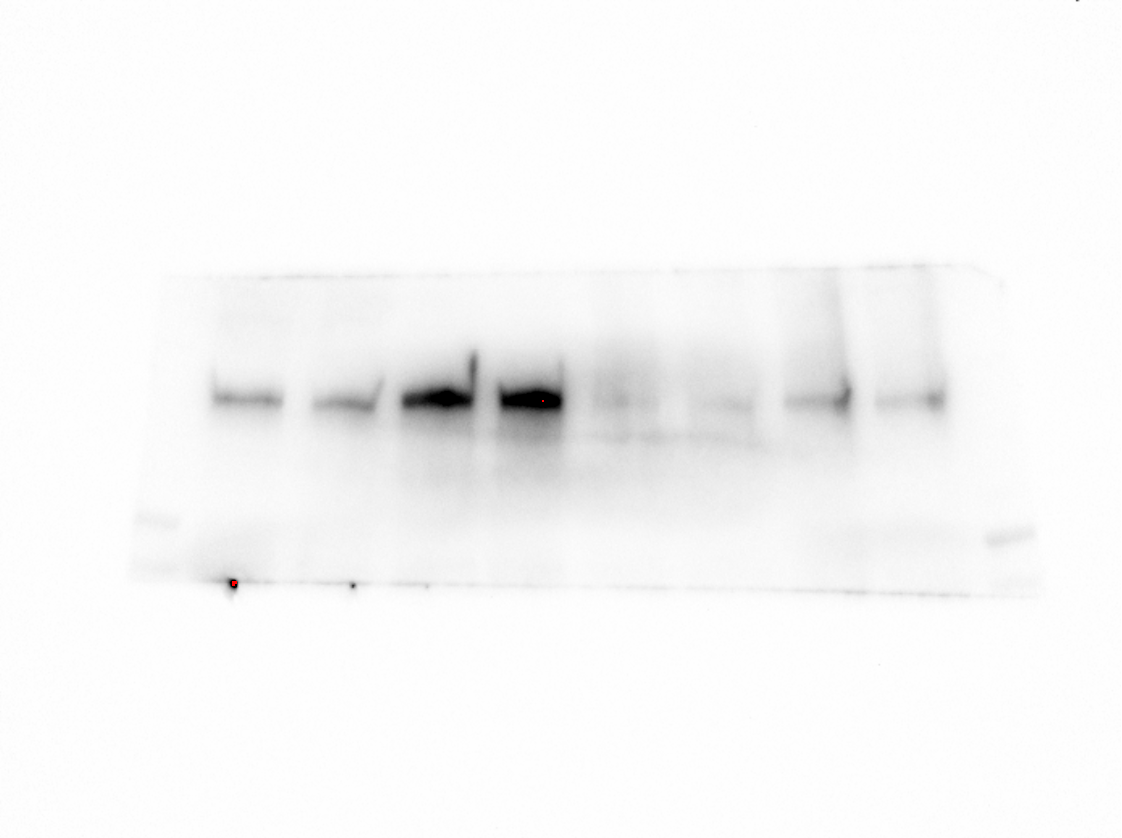

Supplement: Figure 2—figure supplement 1—source data 1. [file elife-98523-fig2-figsupp1-data1.zip › Figure suppl 2/MEF WT KO + EGF ENDOSOME EGFR.tif]

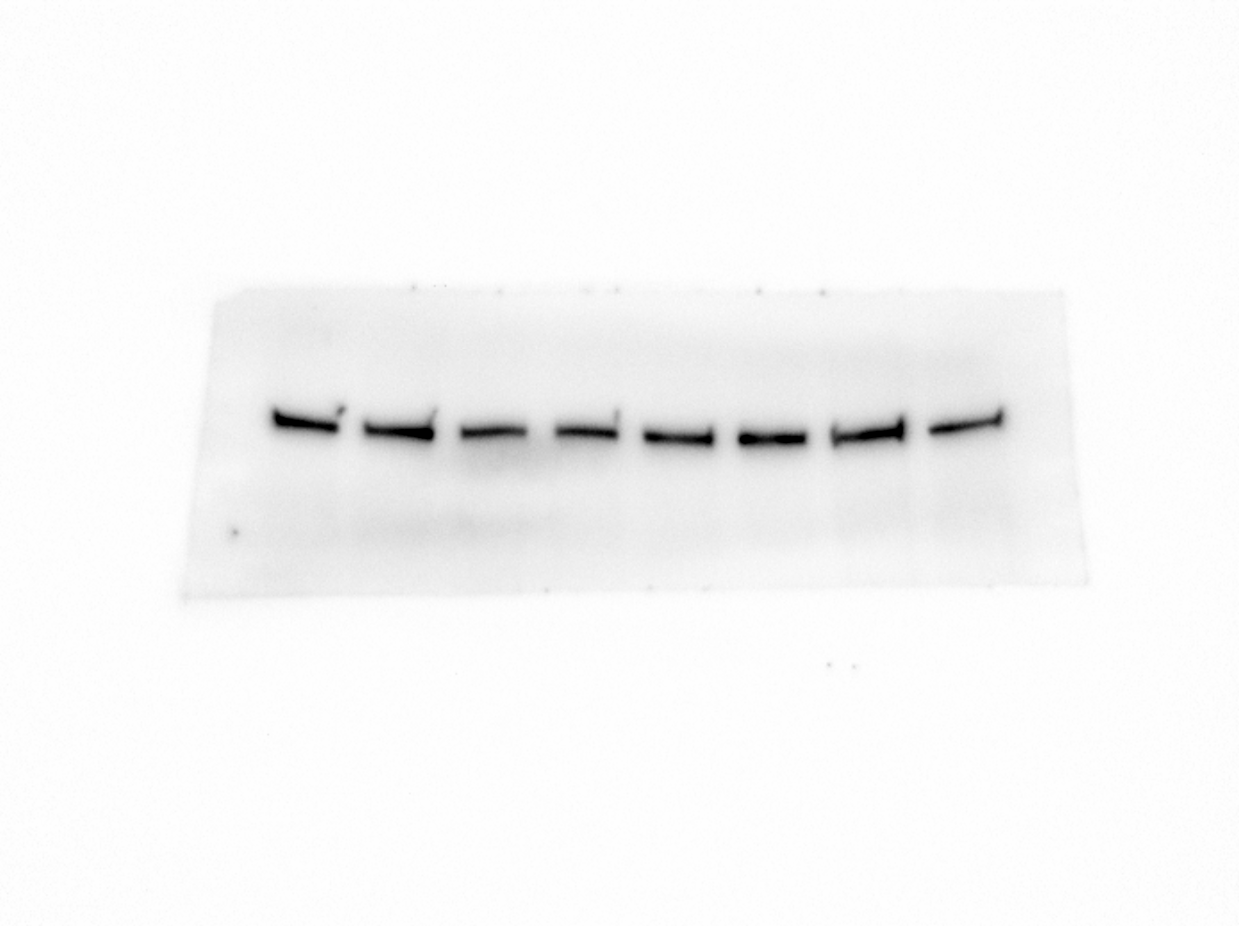

Supplement: Figure 2—figure supplement 1—source data 1. [file elife-98523-fig2-figsupp1-data1.zip › Figure suppl 2/MEF WT KO + EGF ENDOSOME EEA1.tif]

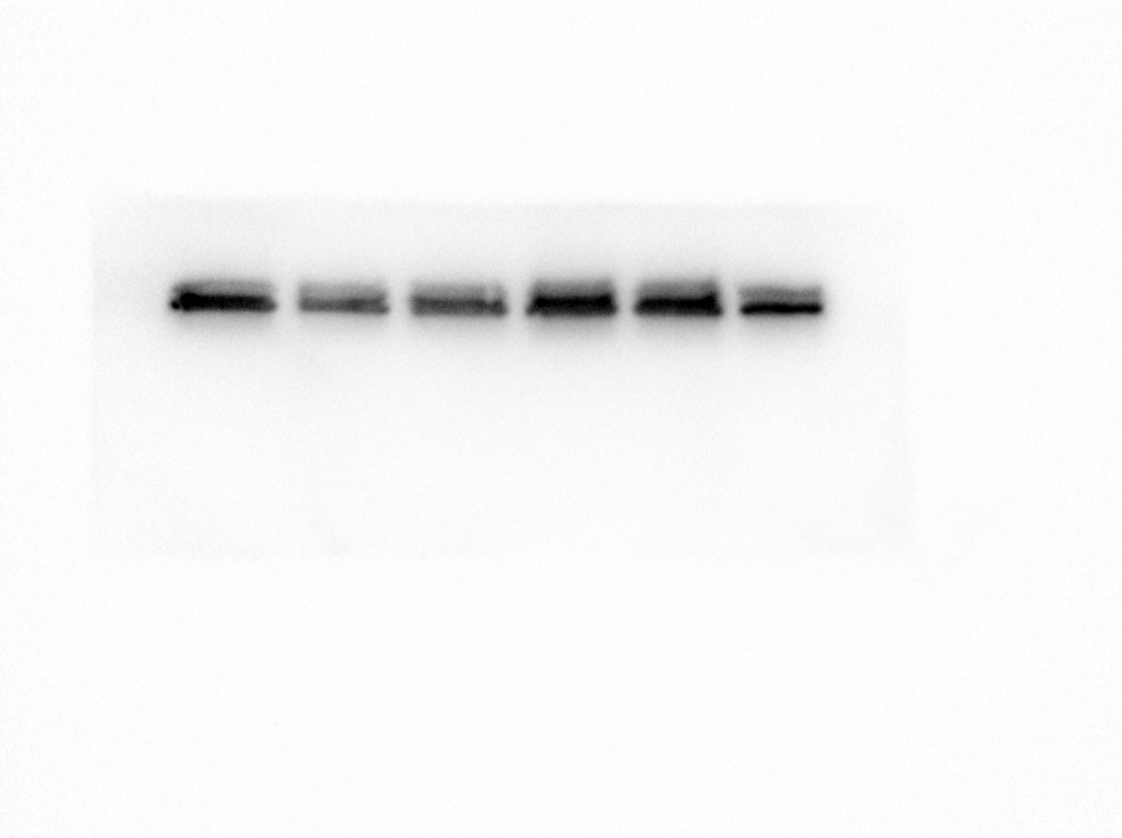

Supplement: Figure 2—figure supplement 1—source data 1. [file elife-98523-fig2-figsupp1-data1.zip › Figure suppl 2/MEF WT KO P44:42.tif]

Figure Supplementary 2 f

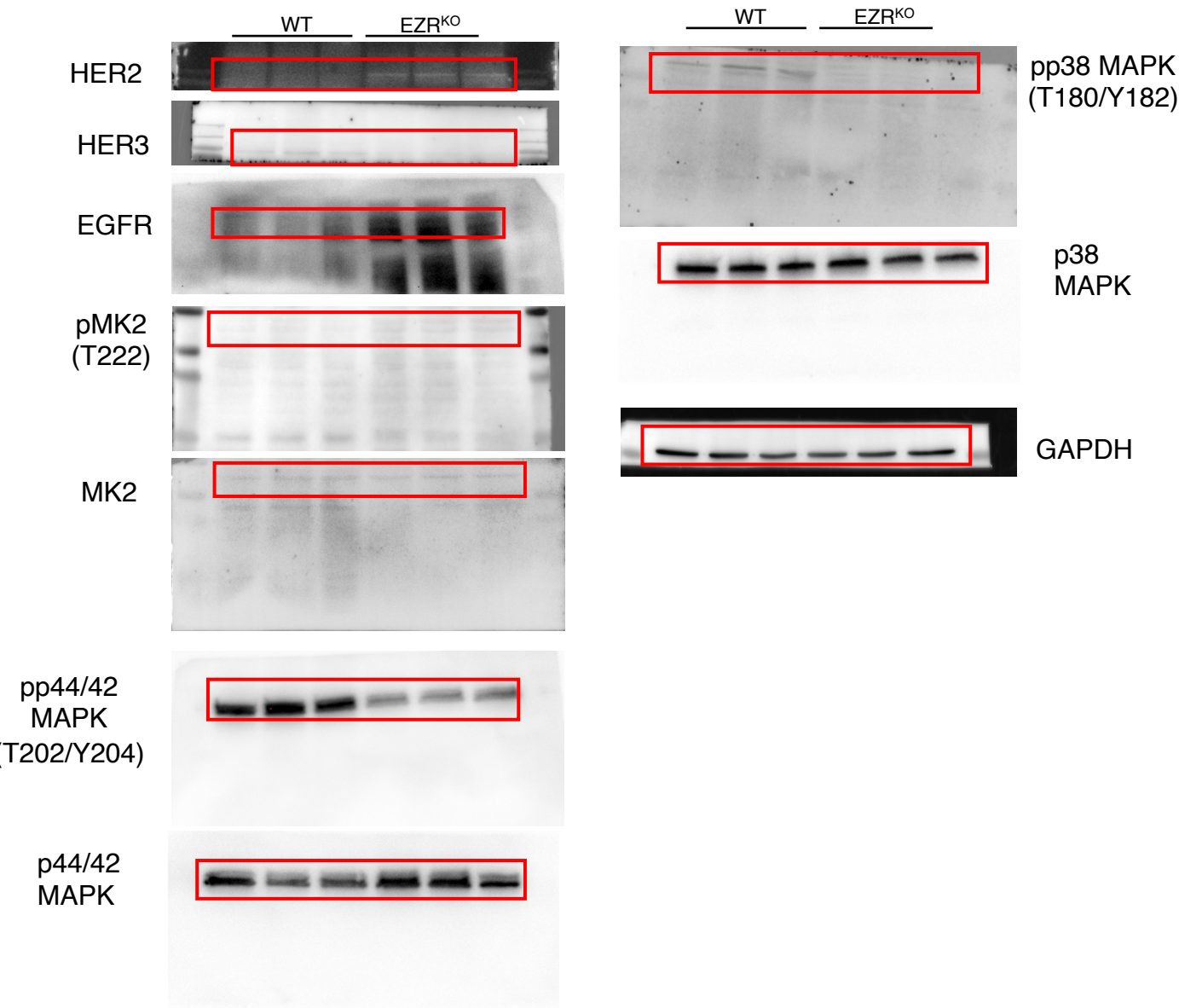

Figure Supplementary 2 g

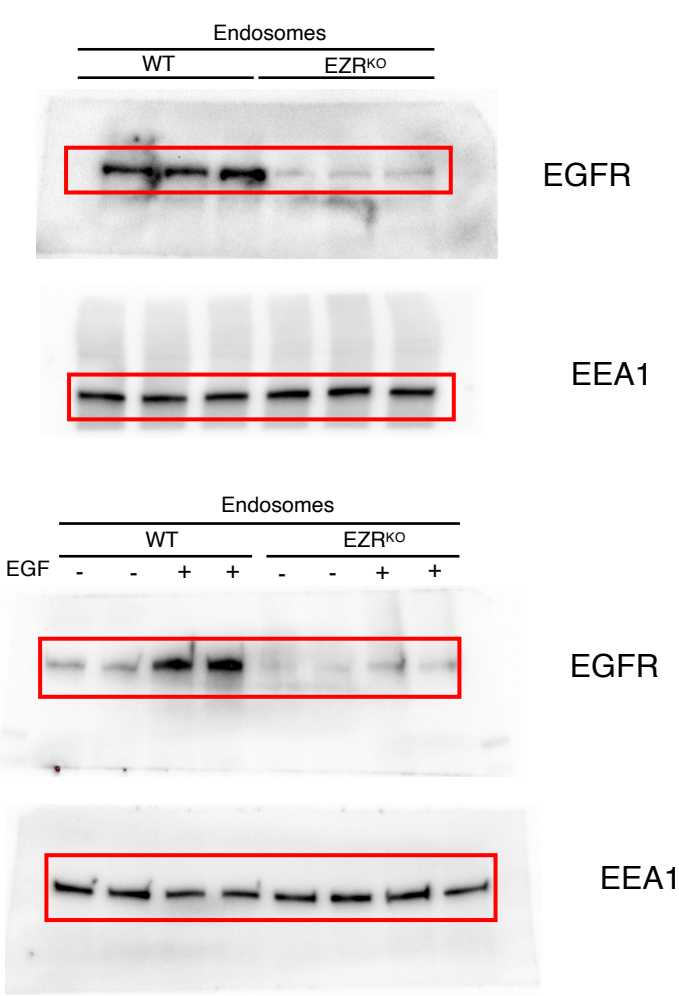

Supplement: Figure 2—figure supplement 1—source data 1. [file elife-98523-fig2-figsupp1-data1.zip › Figure suppl 2/Figure suppl. 2.pdf]

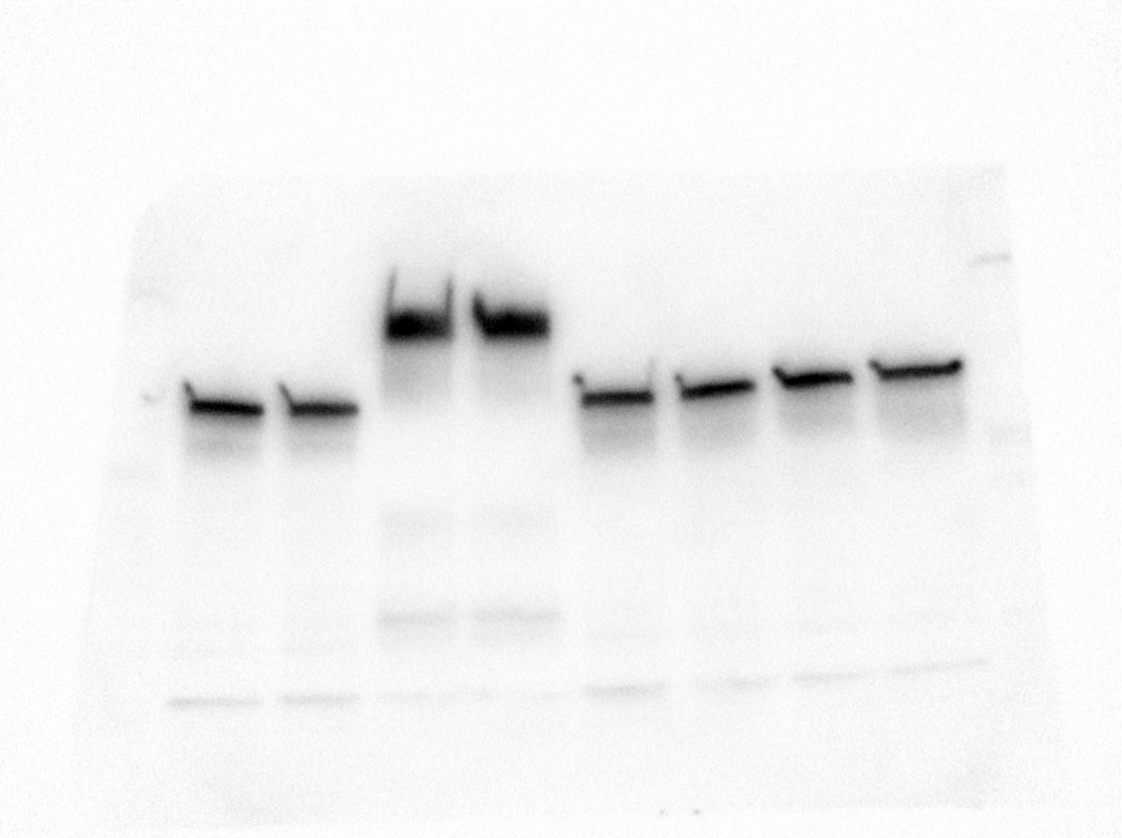

Supplement: Figure 3—source data 1. [file elife-98523-fig3-data1.zip › Figure 3/CROSSLINKING EGFR.tif]

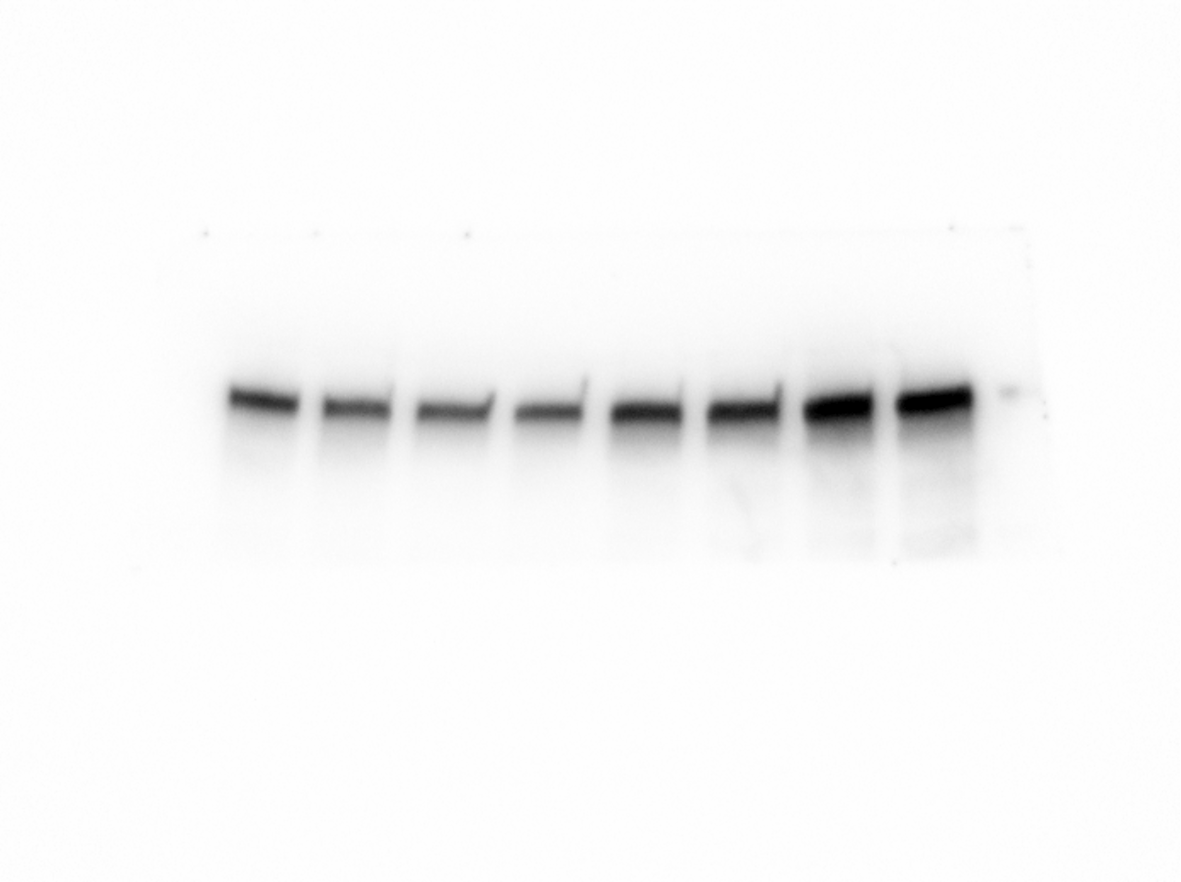

Supplement: Figure 3—source data 1. [file elife-98523-fig3-data1.zip › Figure 3/MEMBRANE EGFR.tif]

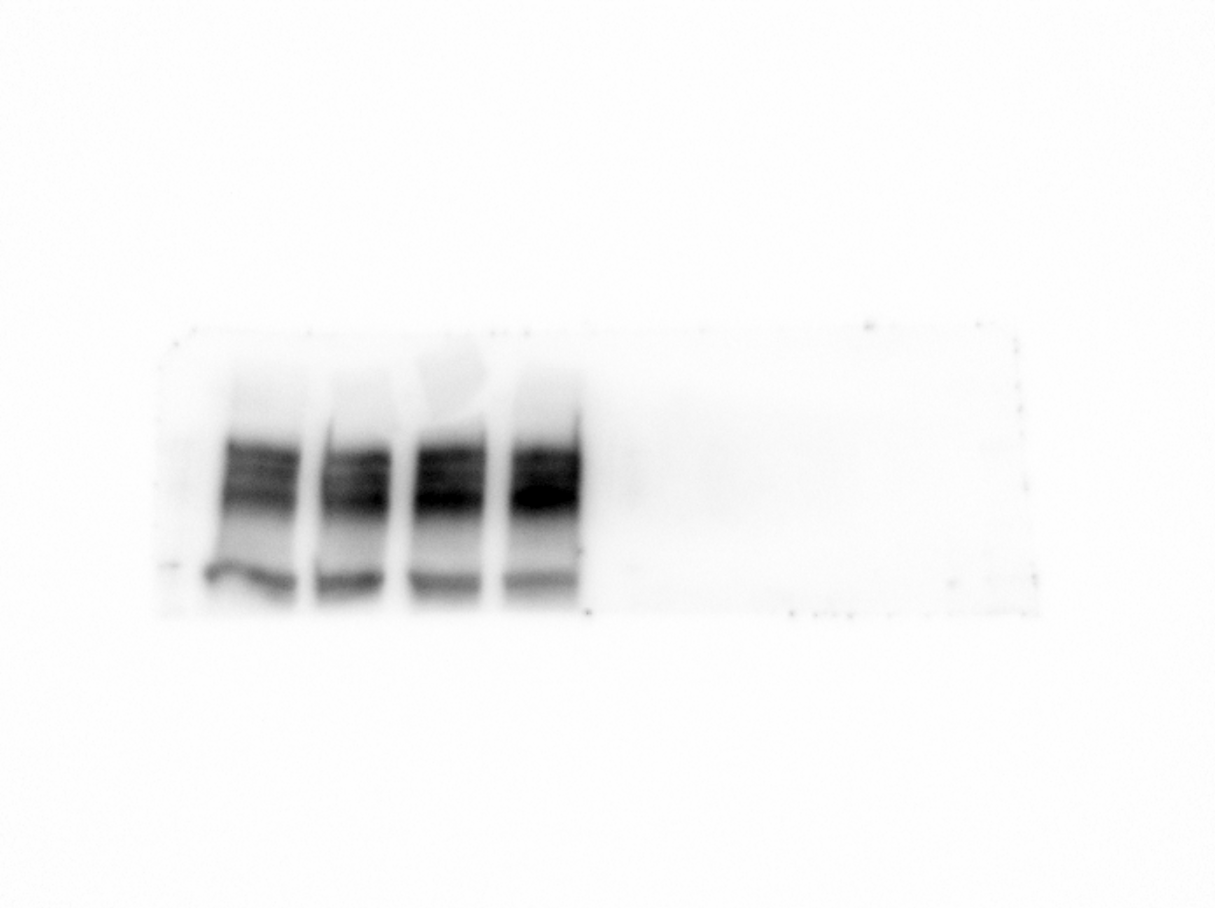

Supplement: Figure 3—source data 1. [file elife-98523-fig3-data1.zip › Figure 3/MEMBRANE ZO1.tif]

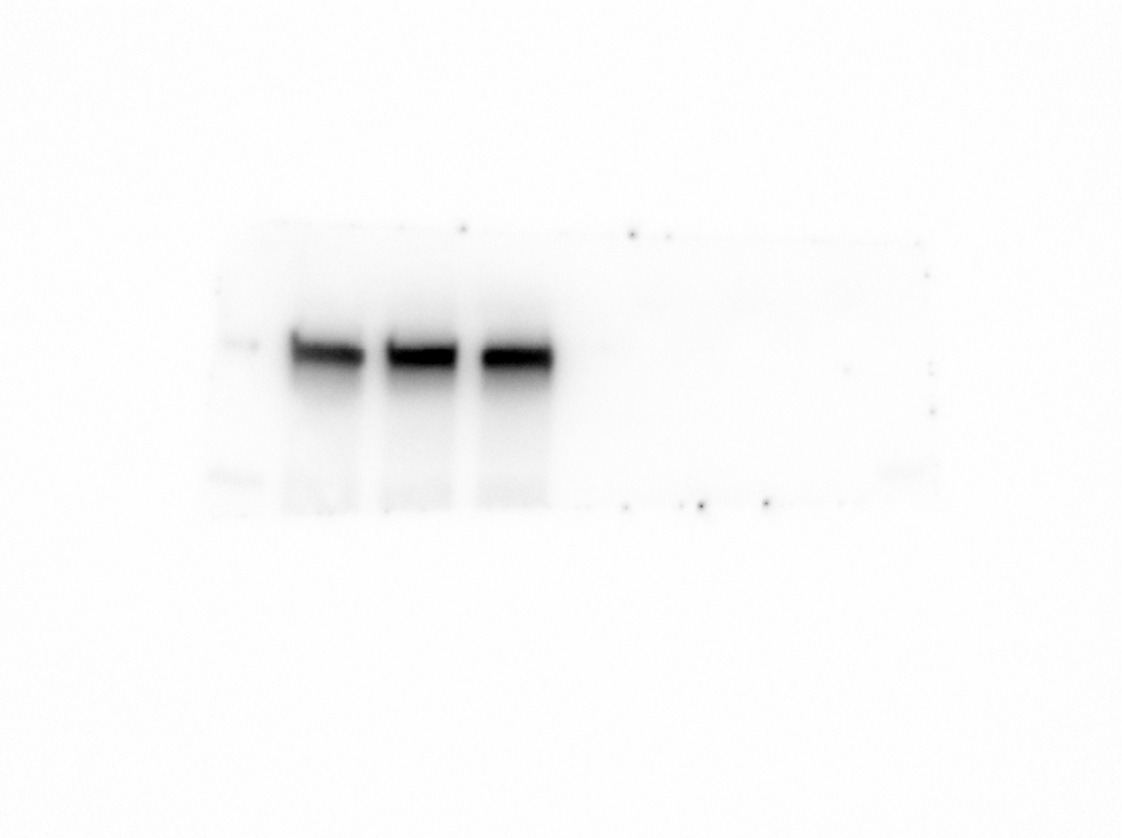

Supplement: Figure 3—source data 1. [file elife-98523-fig3-data1.zip › Figure 3/ENDOSOME EGFR.tif]

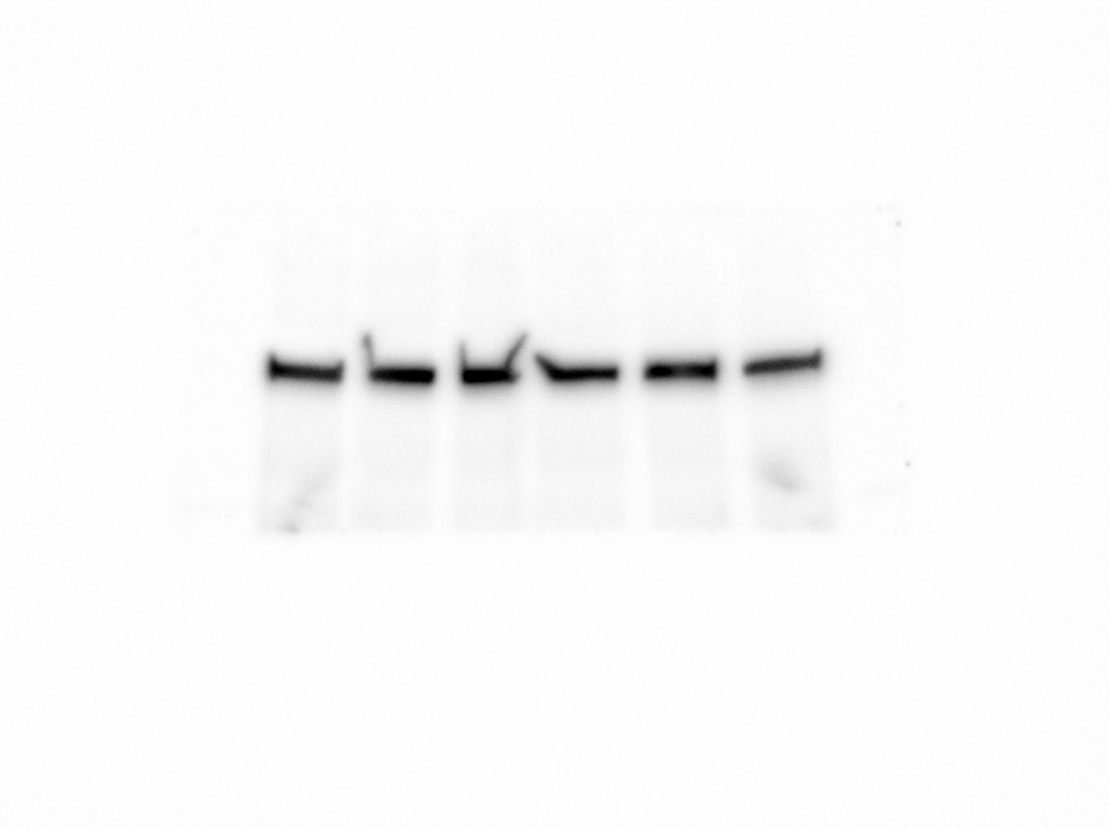

Supplement: Figure 3—source data 1. [file elife-98523-fig3-data1.zip › Figure 3/ENDOSOME EEA1.tif]

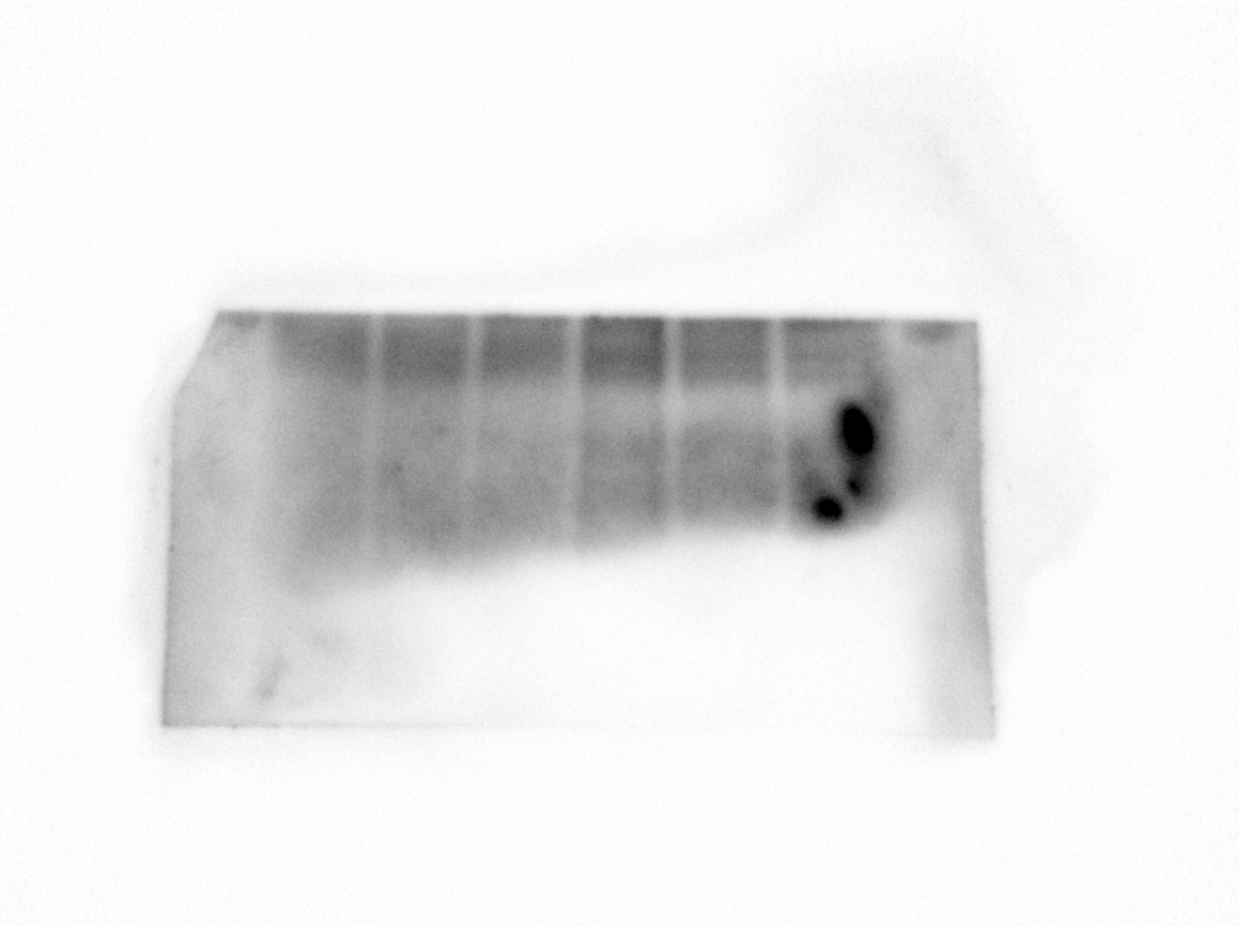

Supplement: Figure 3—source data 1. [file elife-98523-fig3-data1.zip › Figure 3/ENDOSOME GAPDH.tif]

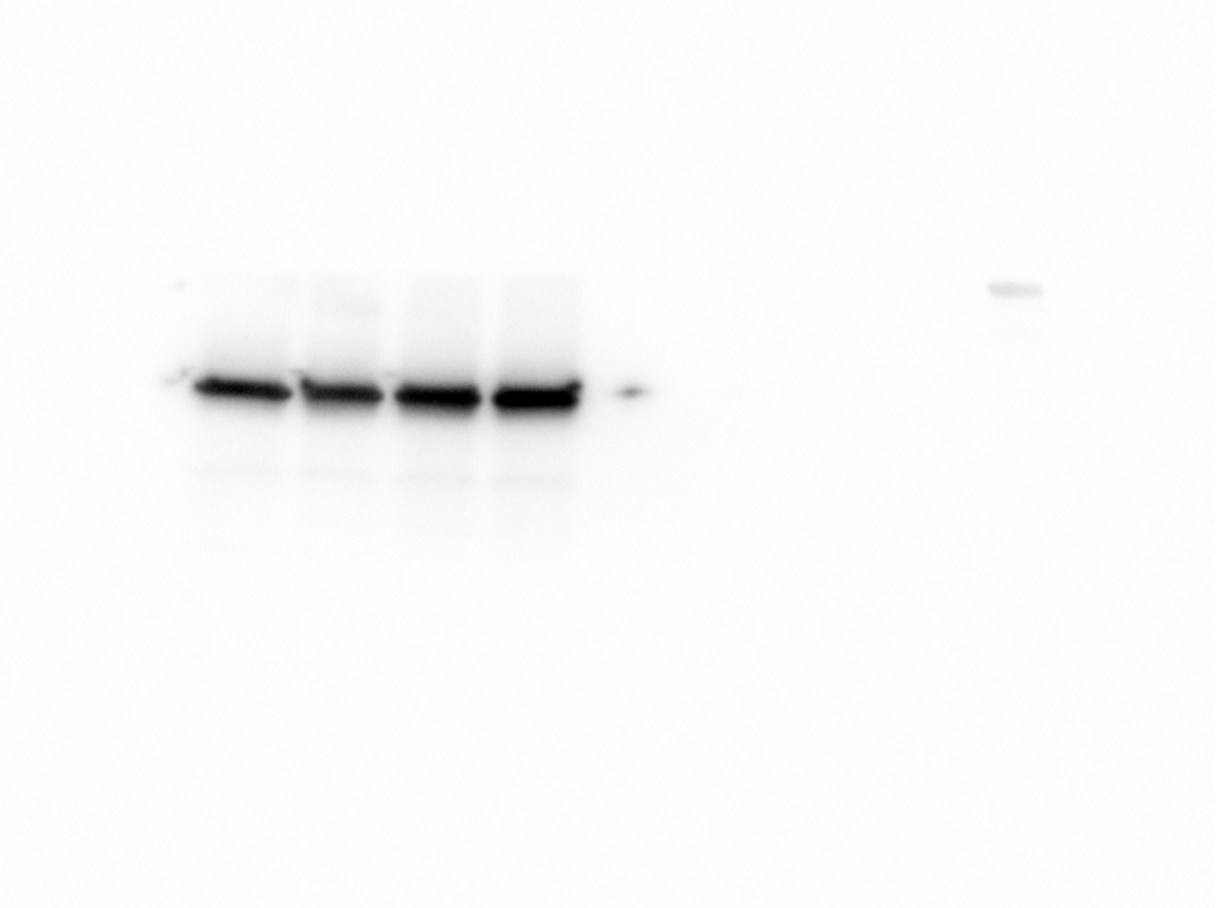

Supplement: Figure 3—source data 1. [file elife-98523-fig3-data1.zip › Figure 3/MEMBRANE GAPDH.tif]

Figure 3 a

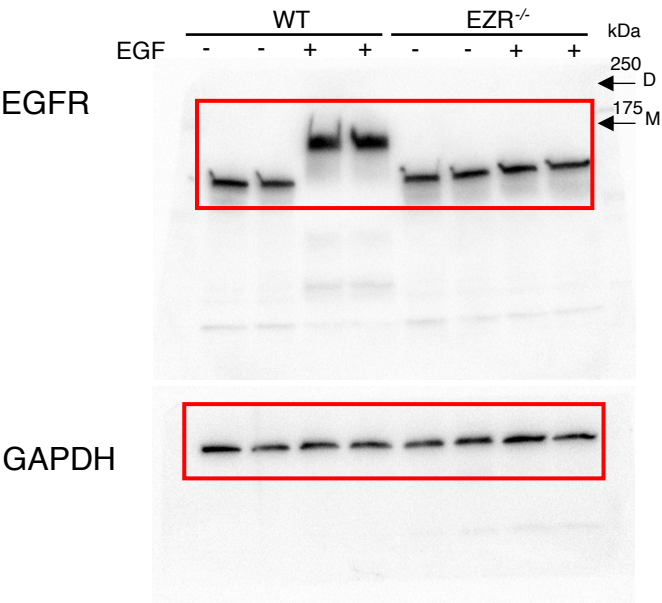

Figure 3 e

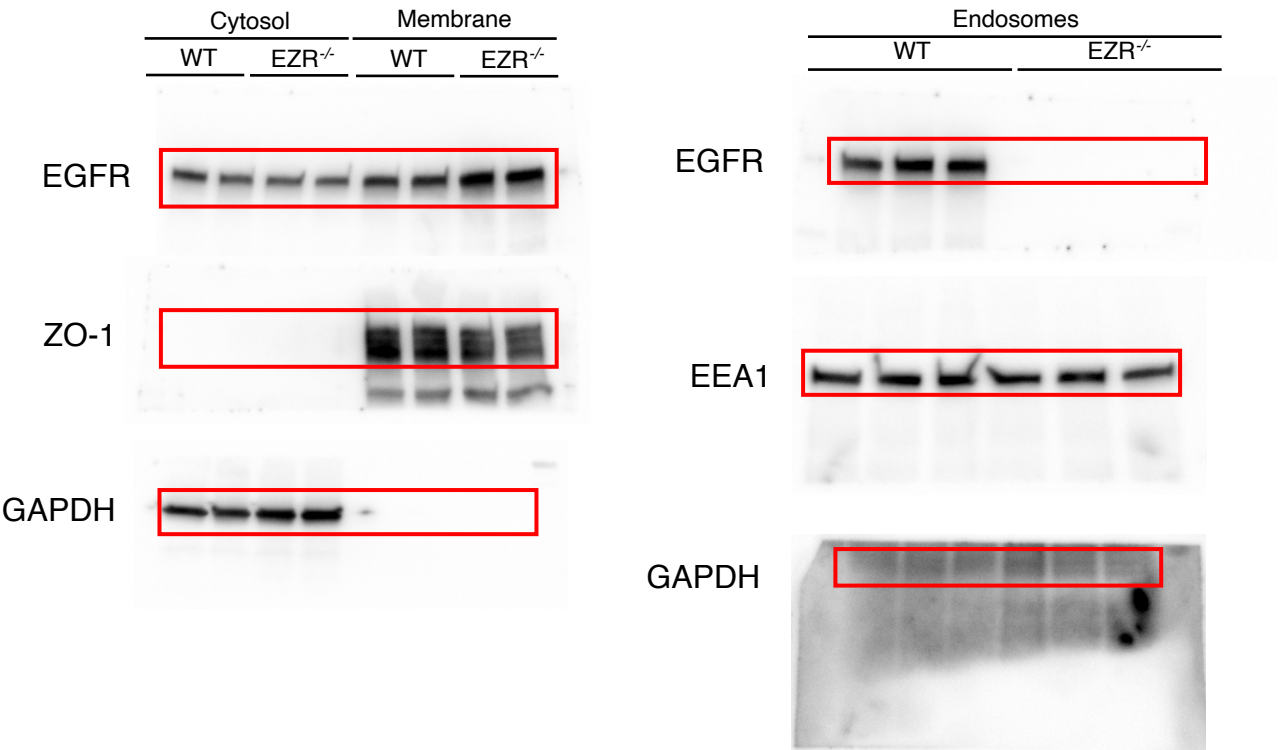

Supplement: Figure 3—source data 1. [file elife-98523-fig3-data1.zip › Figure 3/Figure 3.pdf]

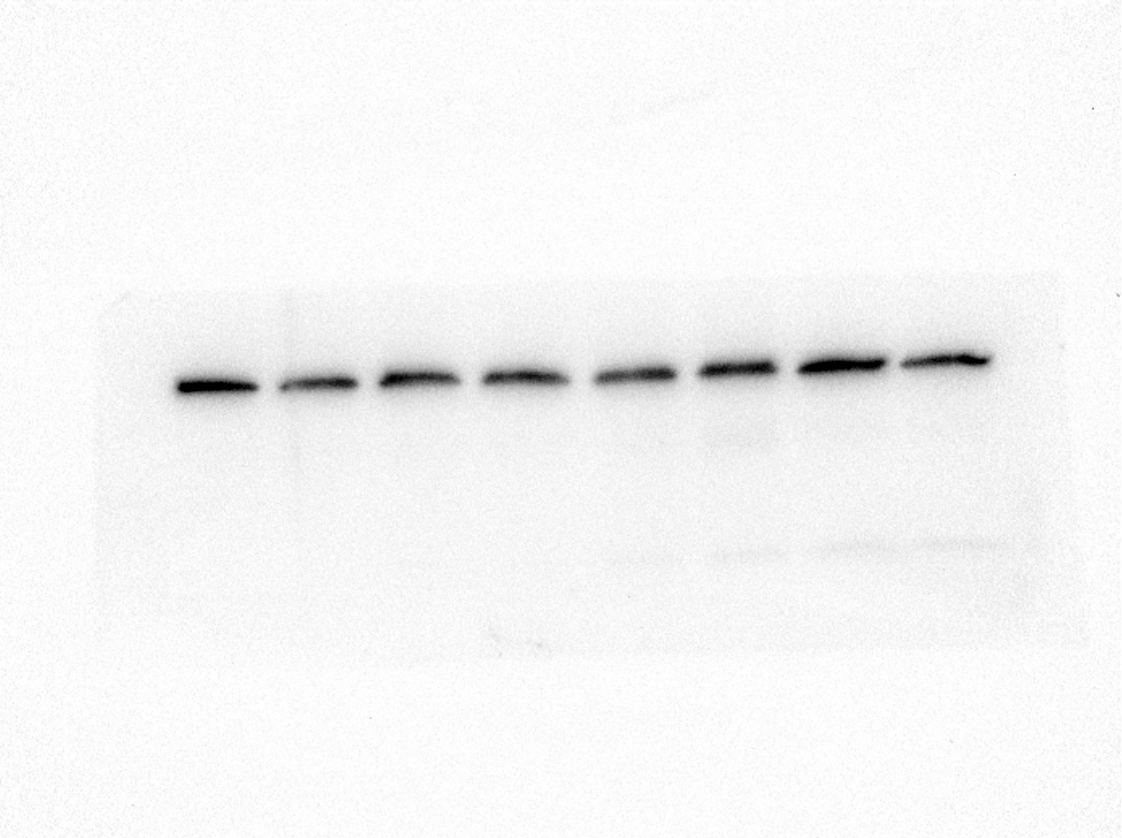

Supplement: Figure 3—source data 1. [file elife-98523-fig3-data1.zip › Figure 3/CROSSLINKING GAPDH .tif]

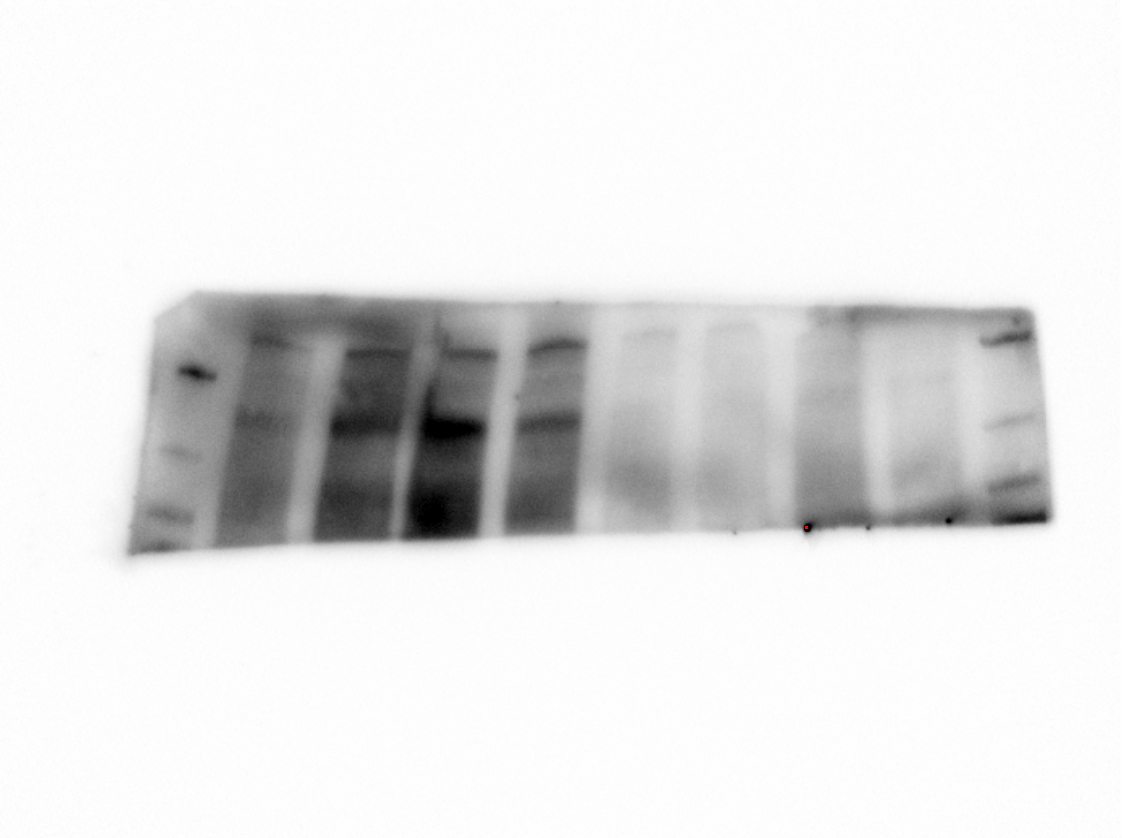

Supplement: Figure 5—source data 1. [file elife-98523-fig5-data1.zip › Figure 5/HELA WT KO + EGFR pEGFR Y1068.tif]

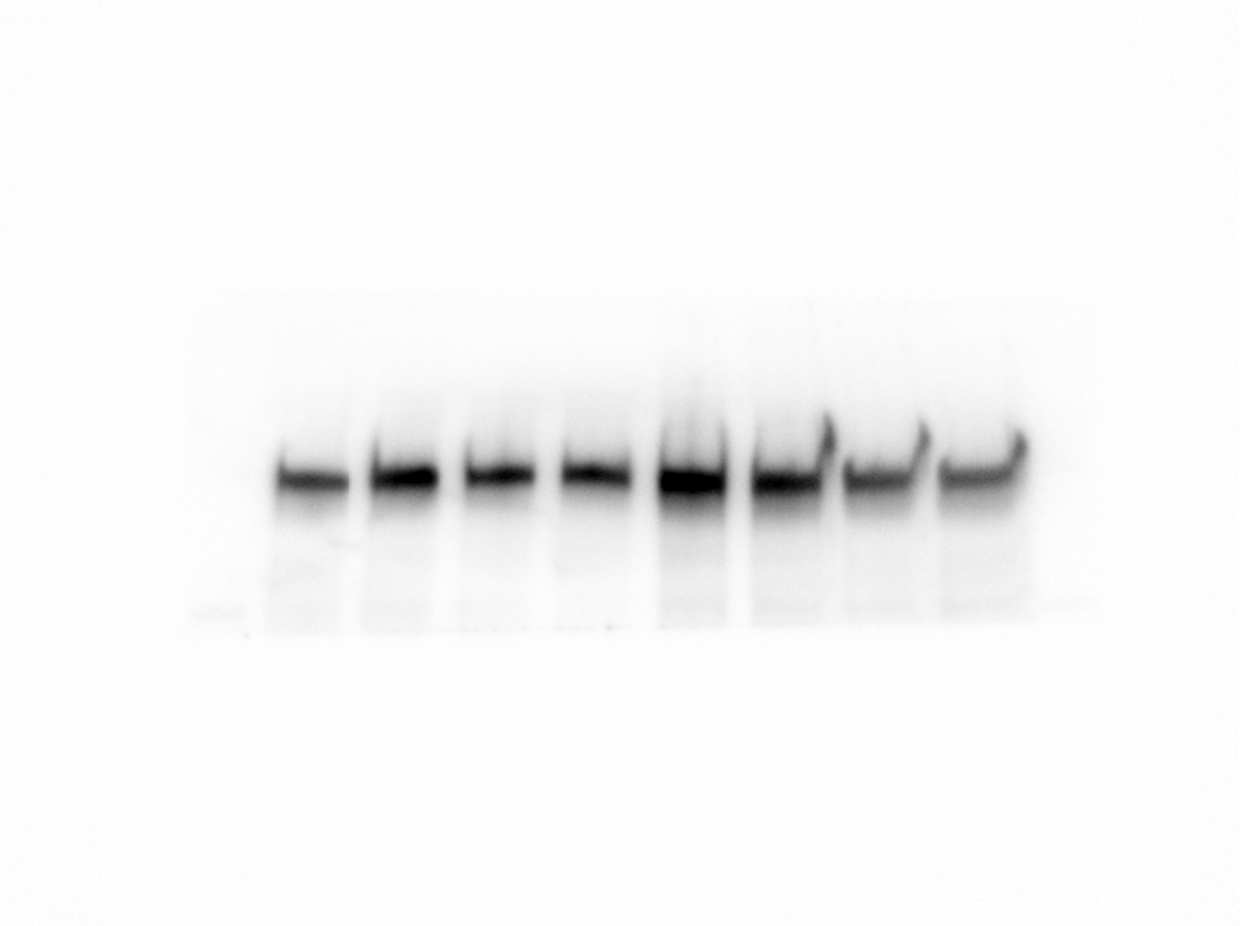

Supplement: Figure 5—source data 1. [file elife-98523-fig5-data1.zip › Figure 5/HELA WT KO + EGF EGFR.tif]

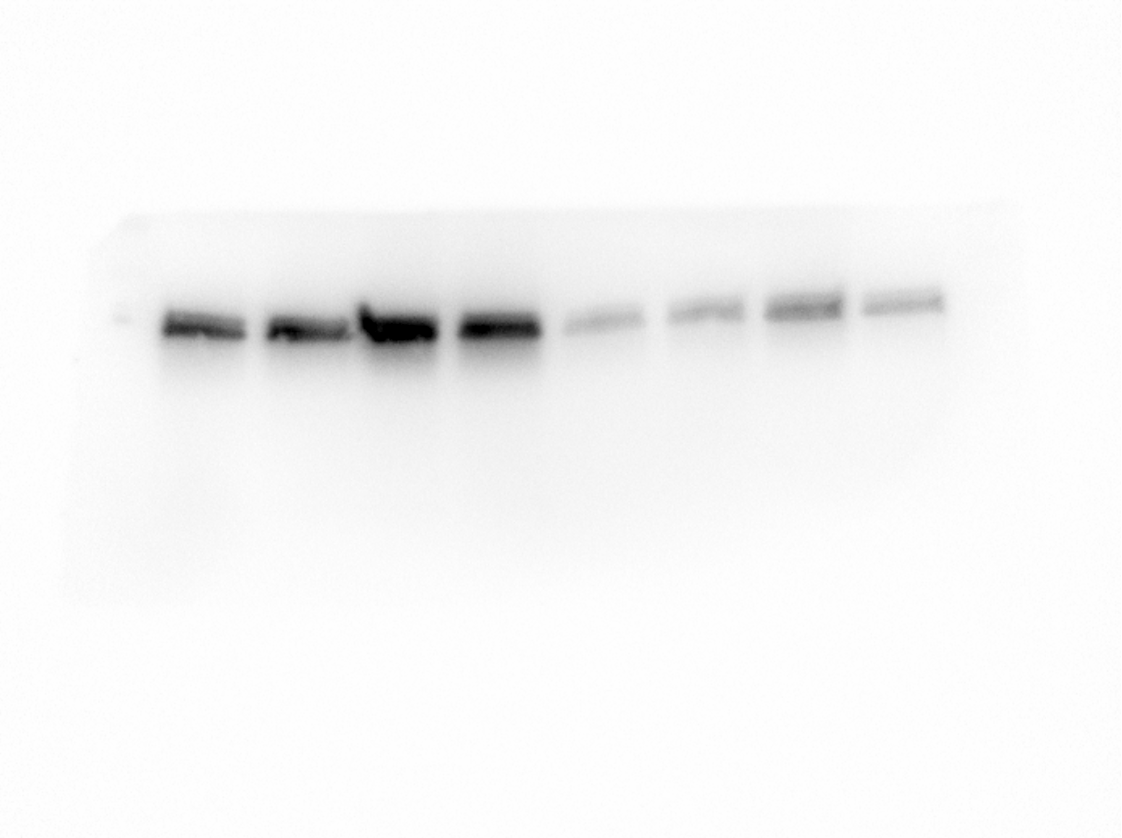

Supplement: Figure 5—source data 1. [file elife-98523-fig5-data1.zip › Figure 5/HELA WT KO + EGFR pP44:42 .tif]

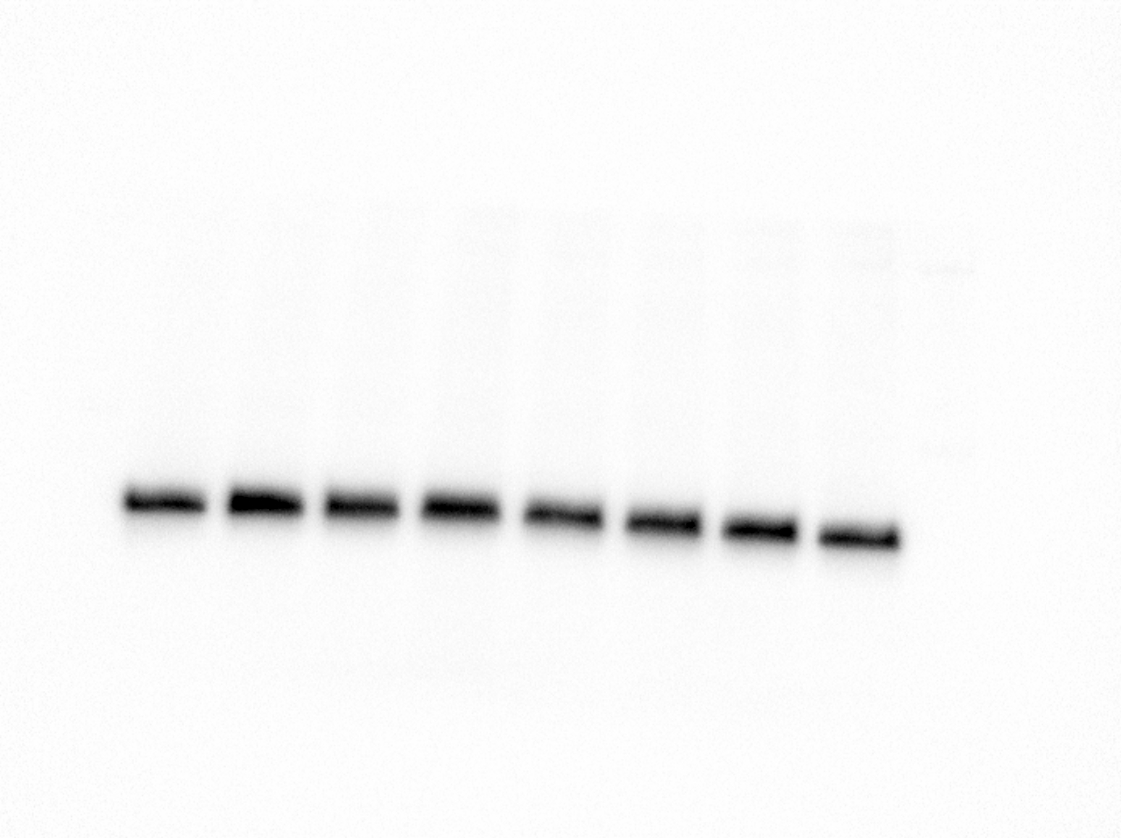

Supplement: Figure 5—source data 1. [file elife-98523-fig5-data1.zip › Figure 5/HELA WT KO + EGF P44:42.tif]

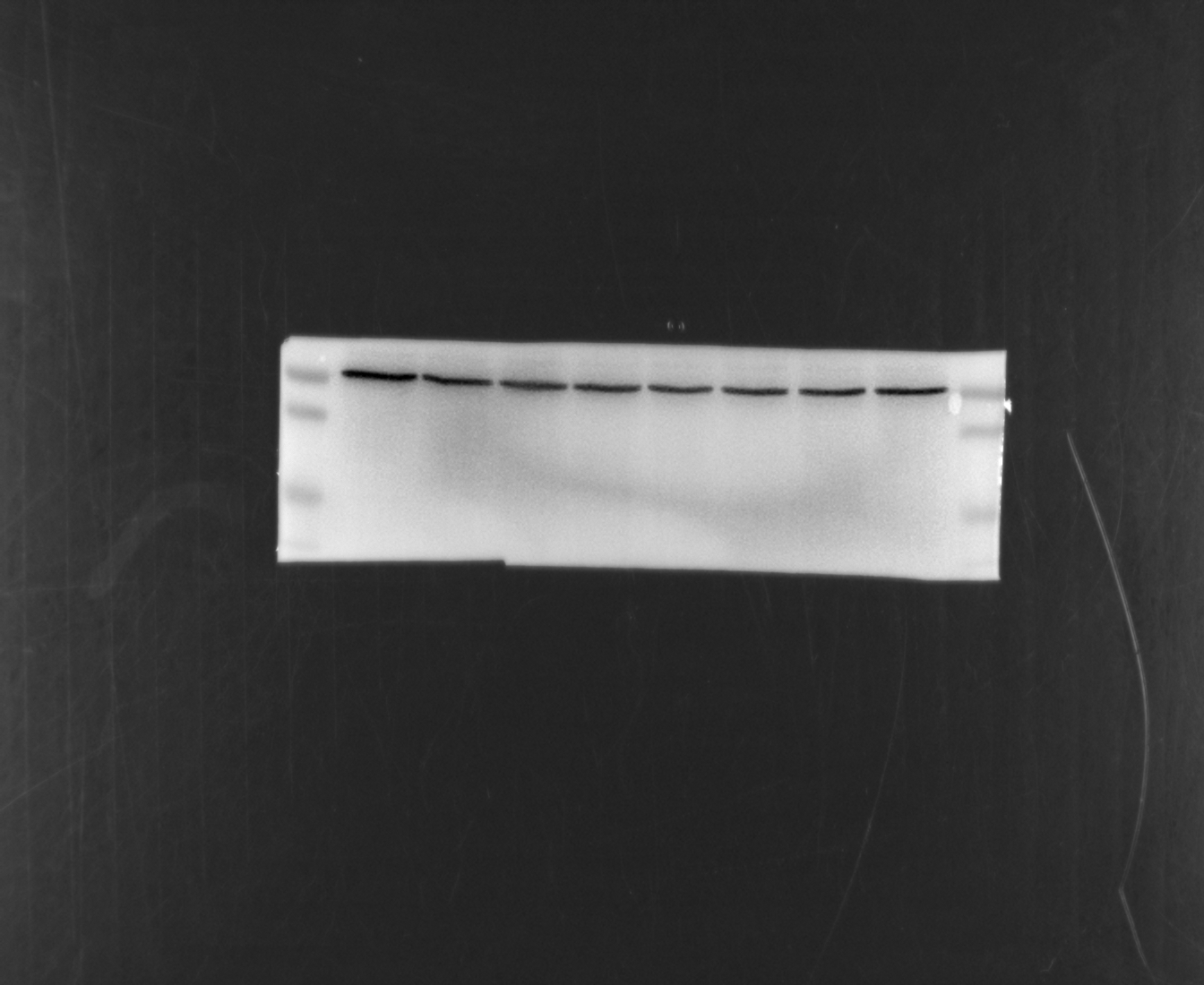

Supplement: Figure 5—source data 1. [file elife-98523-fig5-data1.zip › Figure 5/HELA WT KO + EGF GAPDH.Tif]

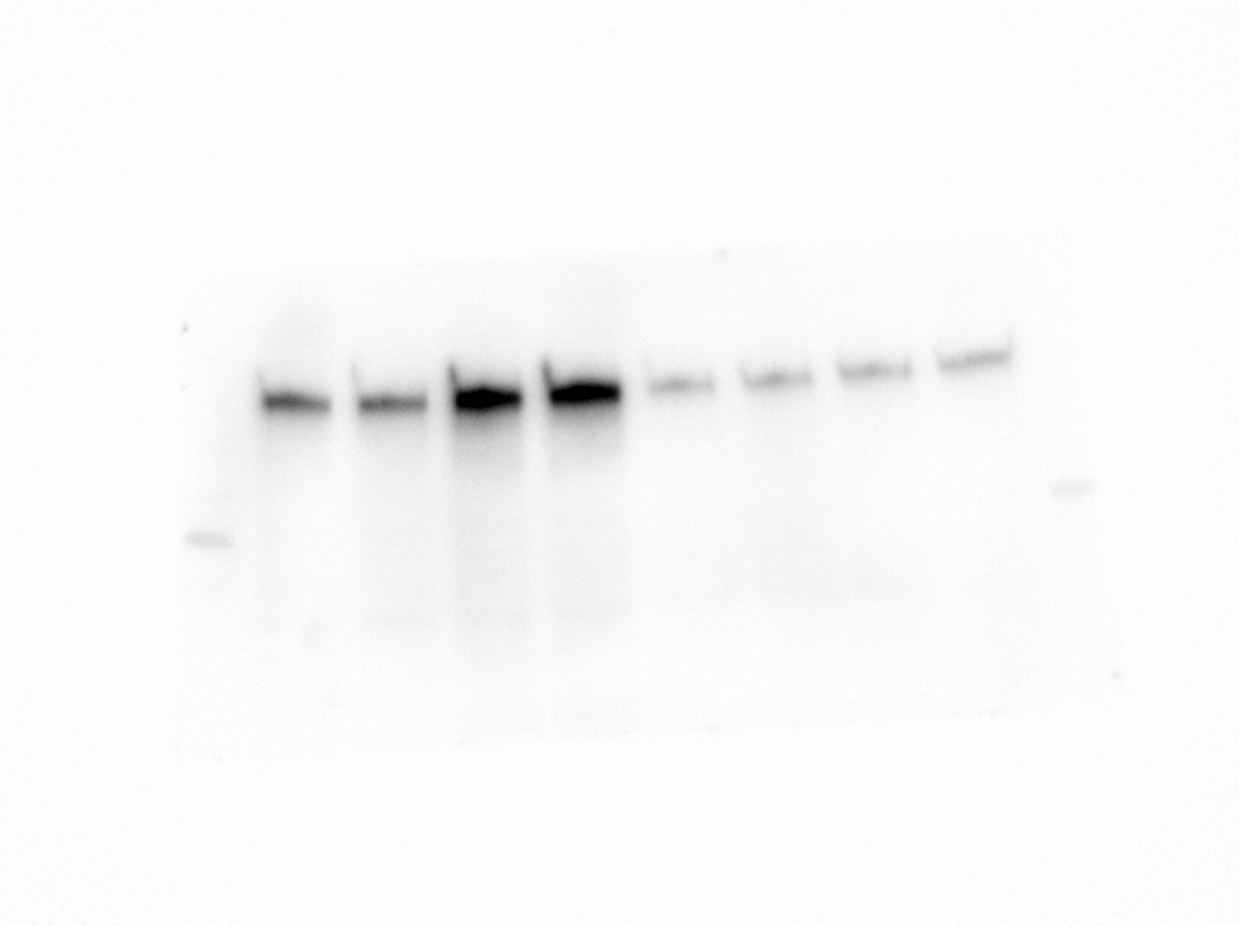

Supplement: Figure 5—source data 1. [file elife-98523-fig5-data1.zip › Figure 5/HELA + EGF ENDOSOME EGFR.tif]

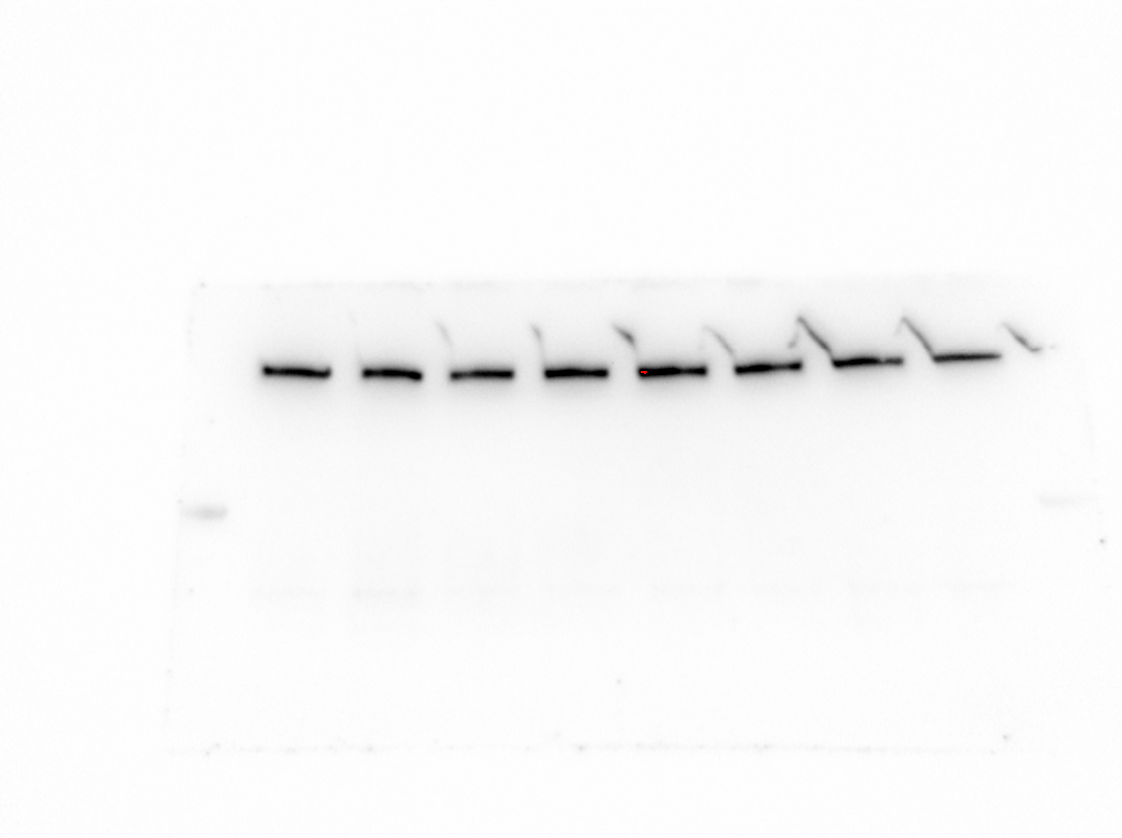

Supplement: Figure 5—source data 1. [file elife-98523-fig5-data1.zip › Figure 5/HELA + EGF ENDOSOME EEA1.tif]

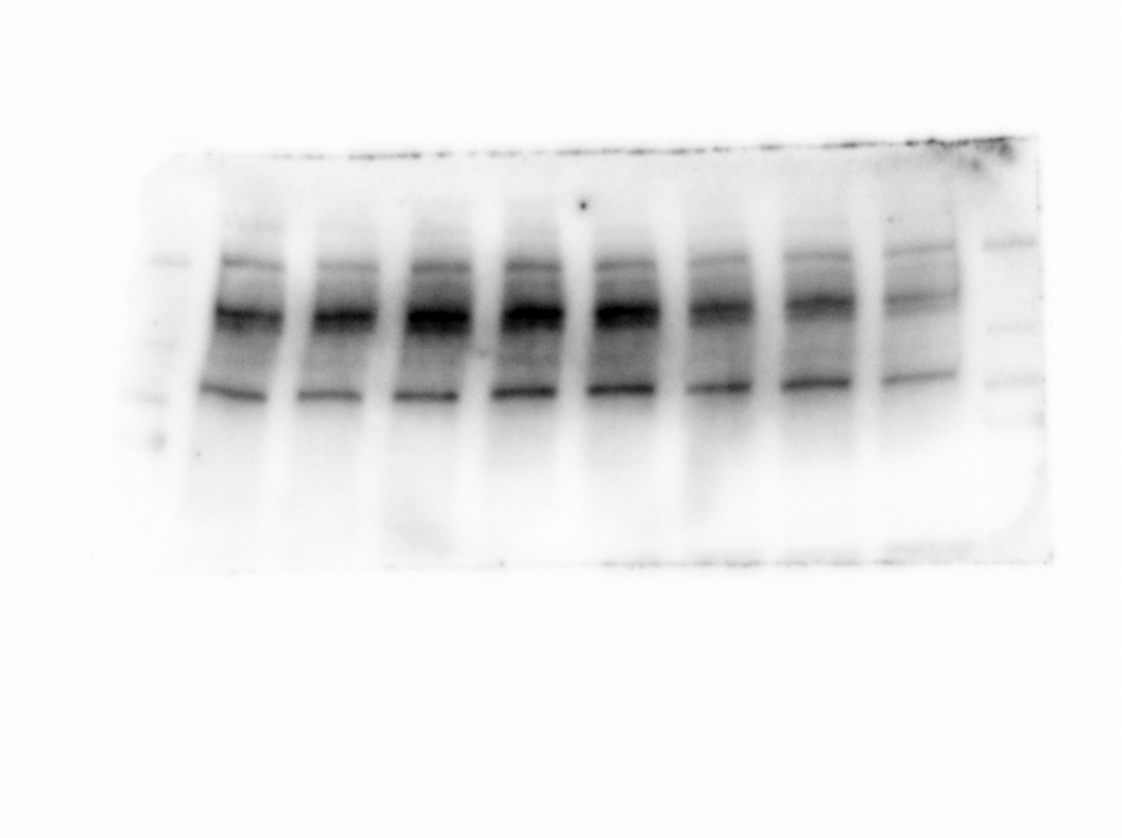

Supplement: Figure 5—source data 1. [file elife-98523-fig5-data1.zip › Figure 5/HELA + EGF MEMBRANE ZO1.tif]

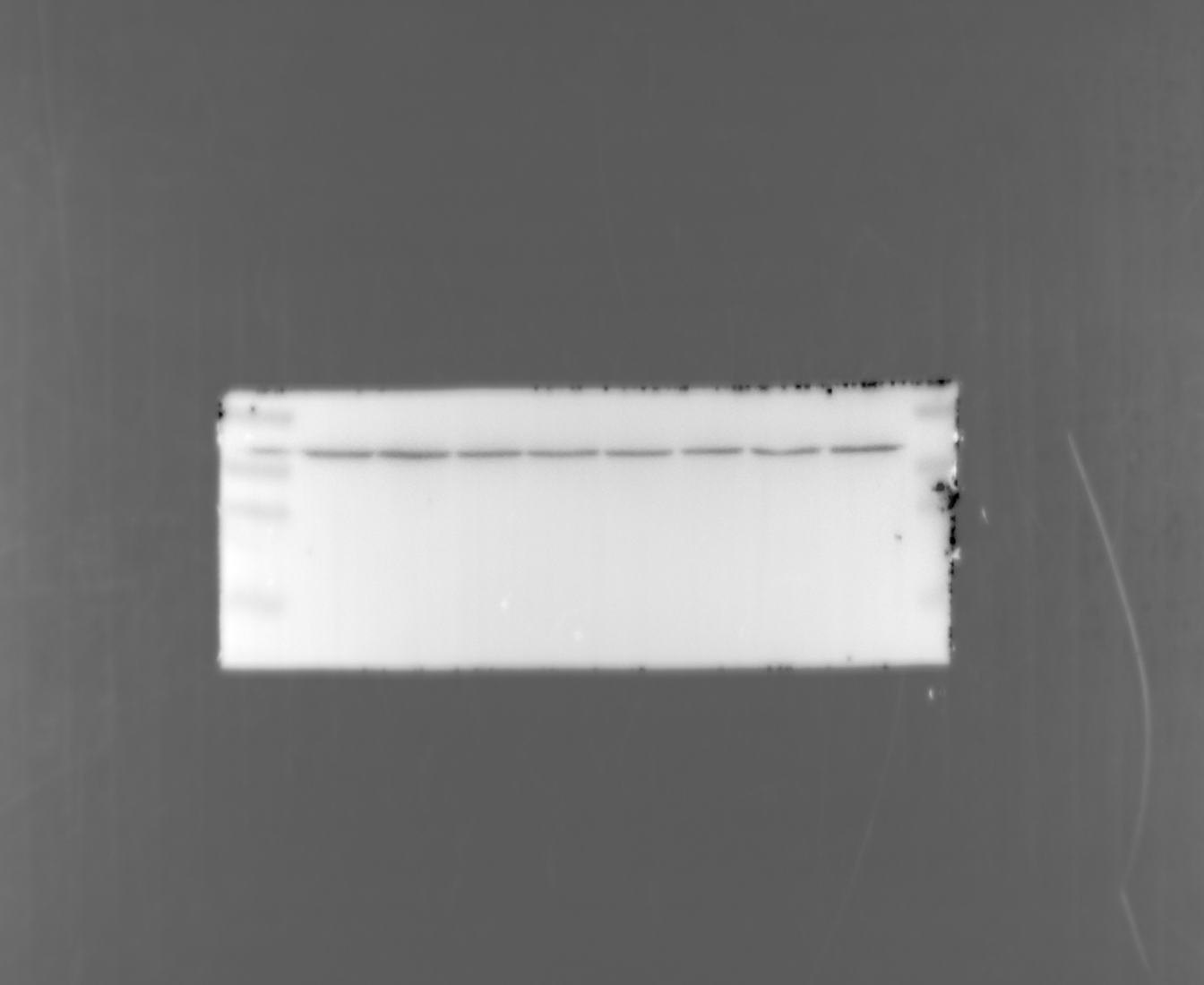

Supplement: Figure 5—source data 1. [file elife-98523-fig5-data1.zip › Figure 5/HELA WT KO + EGF P38.Tif]

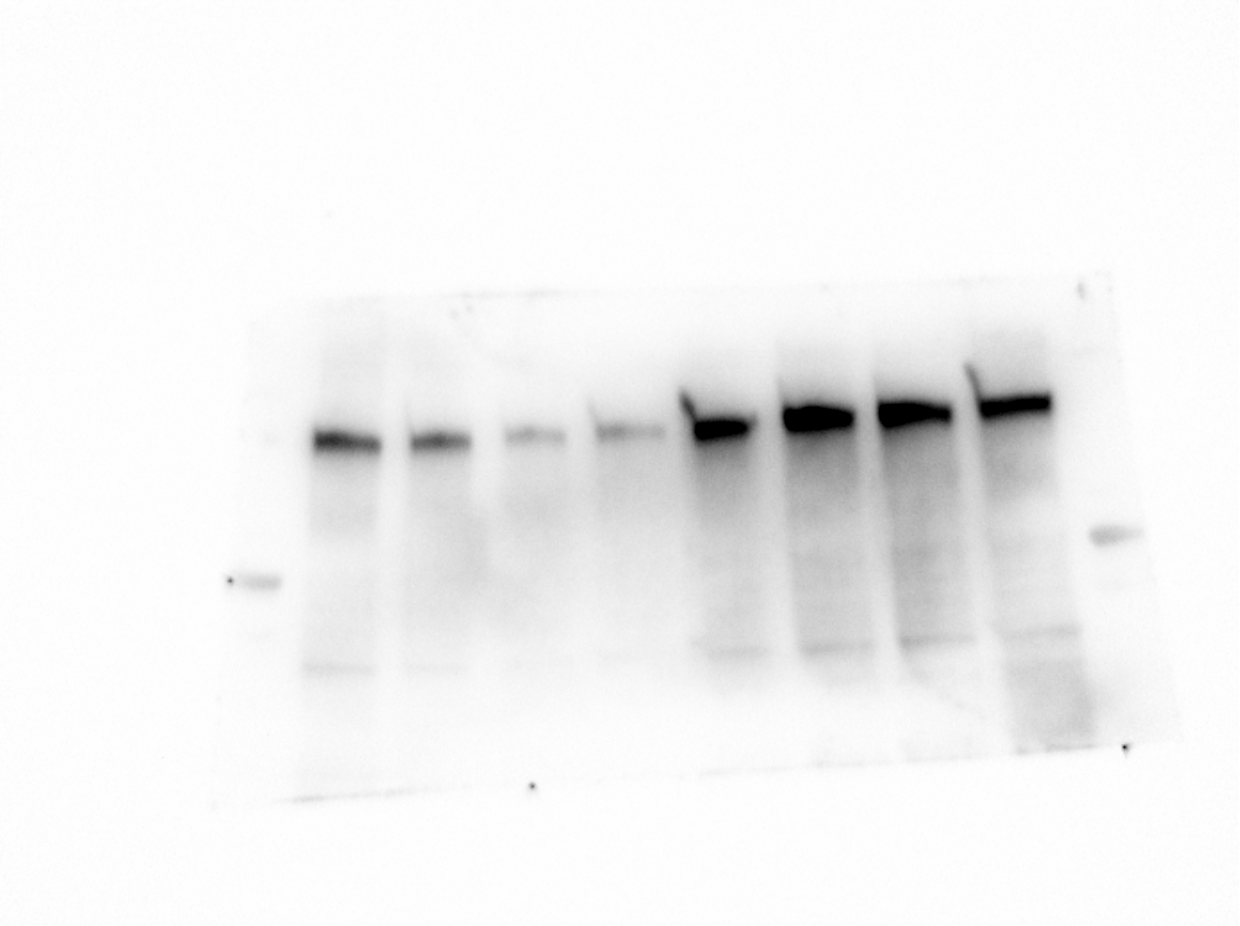

Supplement: Figure 5—source data 1. [file elife-98523-fig5-data1.zip › Figure 5/HELA + EGF MEMBRANE EGFR.tif]

Figure 5 c

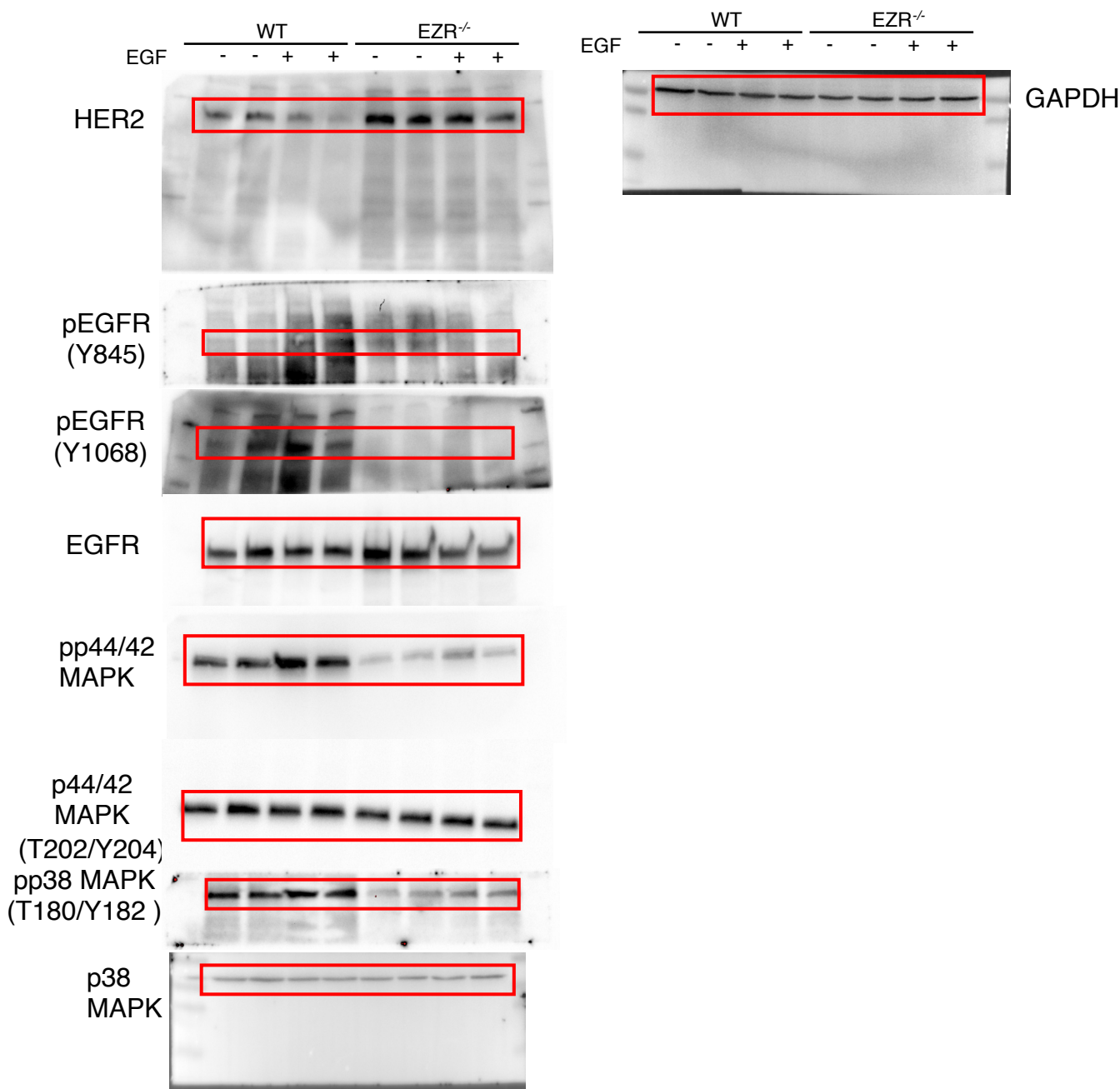

Figure 5 d

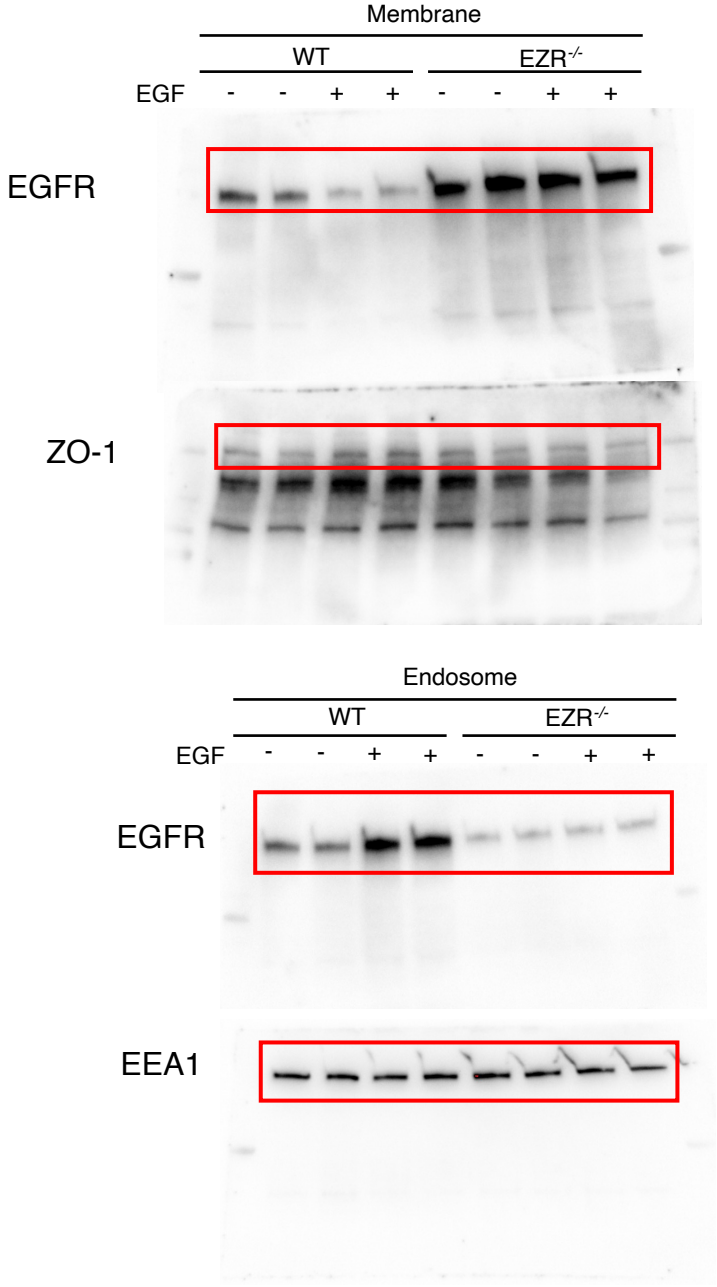

Supplement: Figure 5—source data 1. [file elife-98523-fig5-data1.zip › Figure 5/Figure 5.pdf]

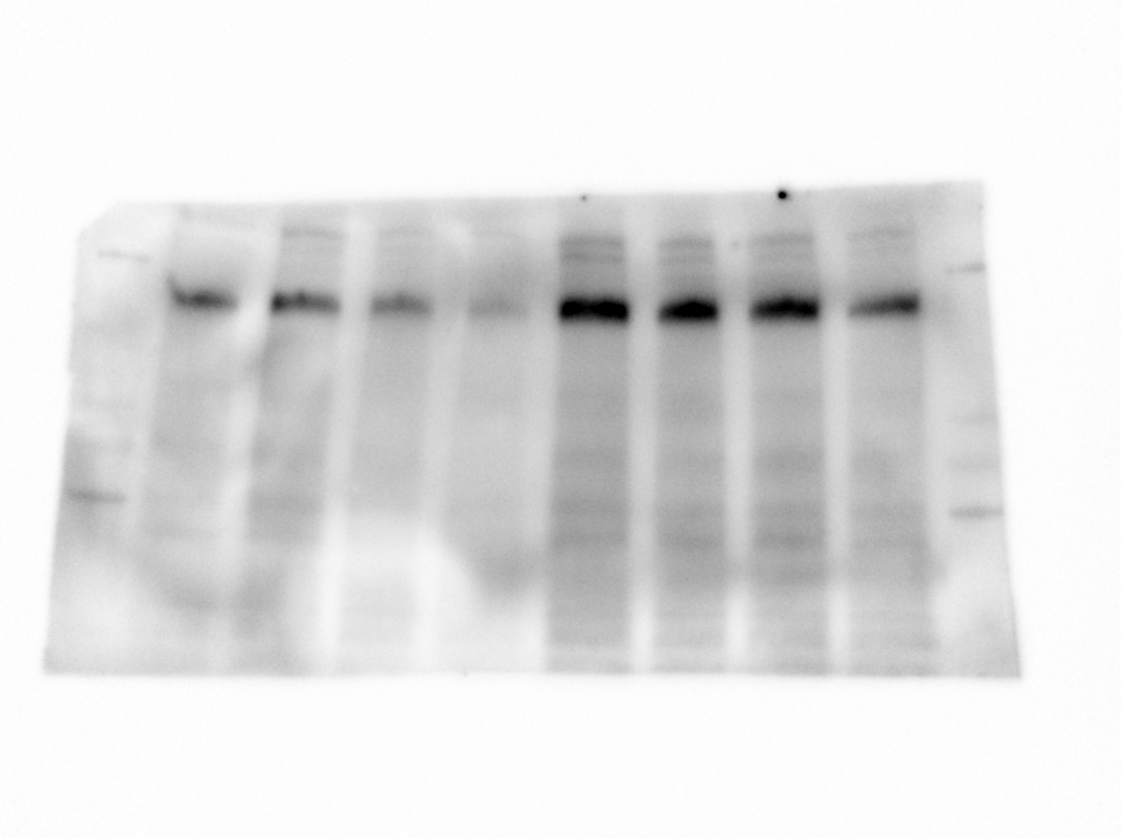

Supplement: Figure 5—source data 1. [file elife-98523-fig5-data1.zip › Figure 5/HELA WT KO + EGF HER2.tif]

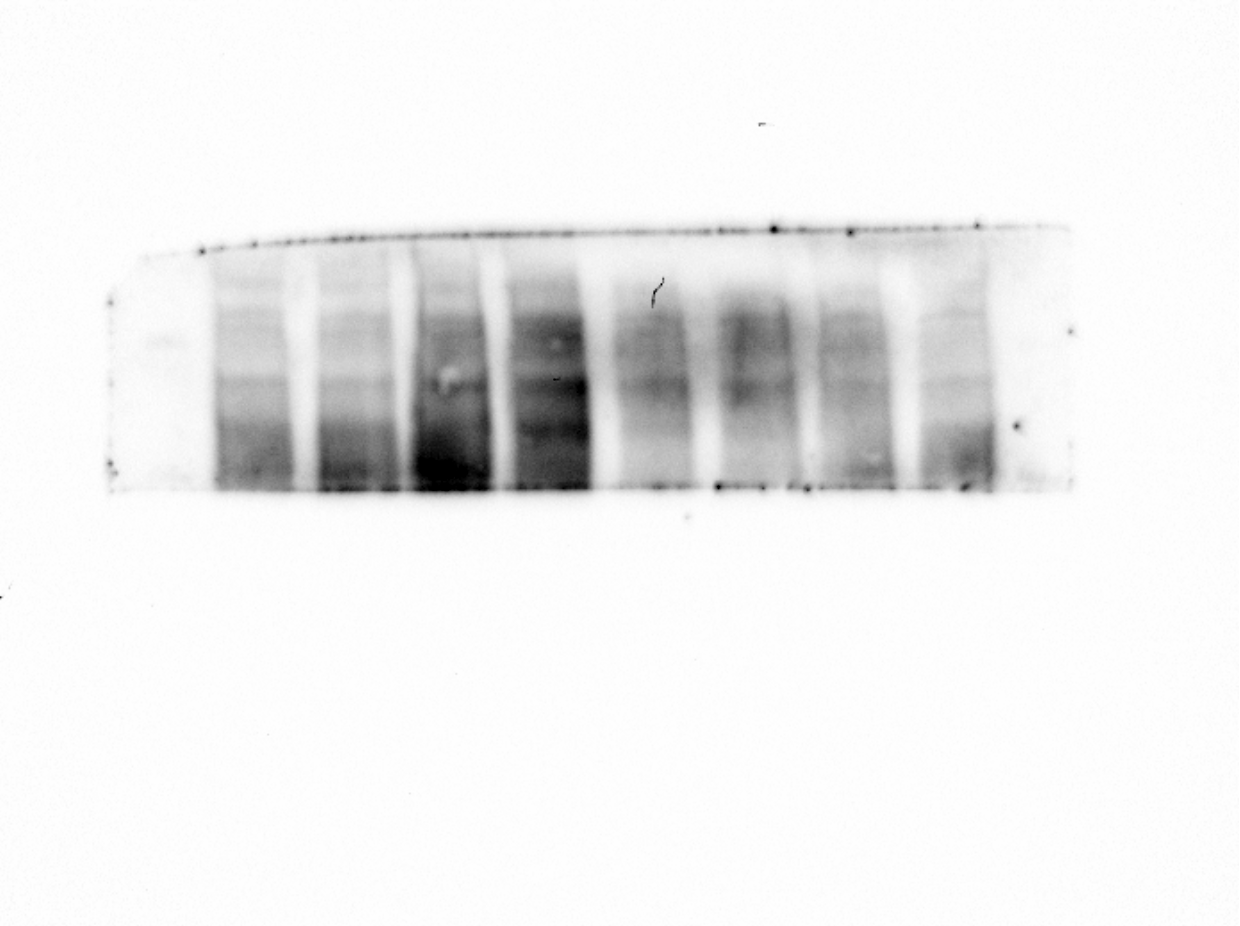

Supplement: Figure 5—source data 1. [file elife-98523-fig5-data1.zip › Figure 5/HELA WT KO + EGF pEGFR Y845.tif]

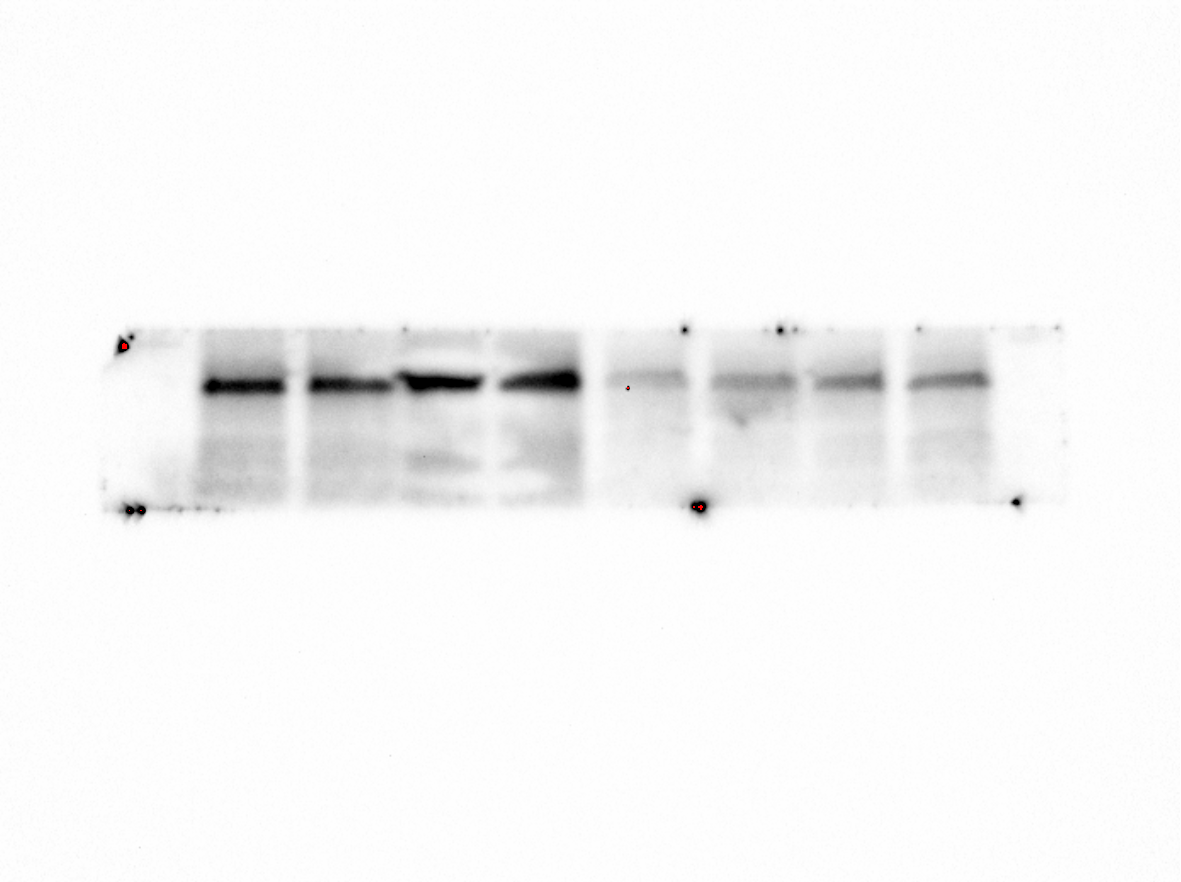

Supplement: Figure 5—source data 1. [file elife-98523-fig5-data1.zip › Figure 5/HELA WT KO + EGF pP38.tif]

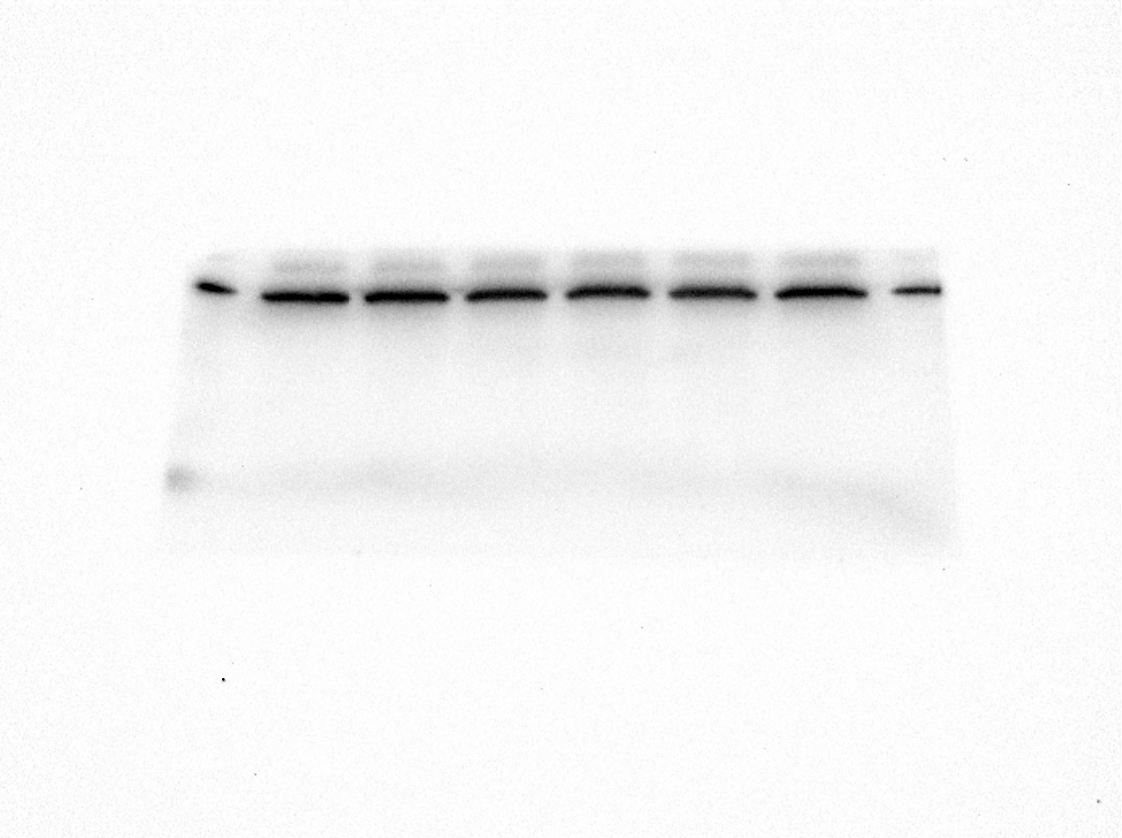

Supplement: Figure 5—figure supplement 1—source data 1. [file elife-98523-fig5-figsupp1-data1.zip › Figure suppl 3/HELA WT DM DR GAPDH.tif]

Figure Supplementary 3 a

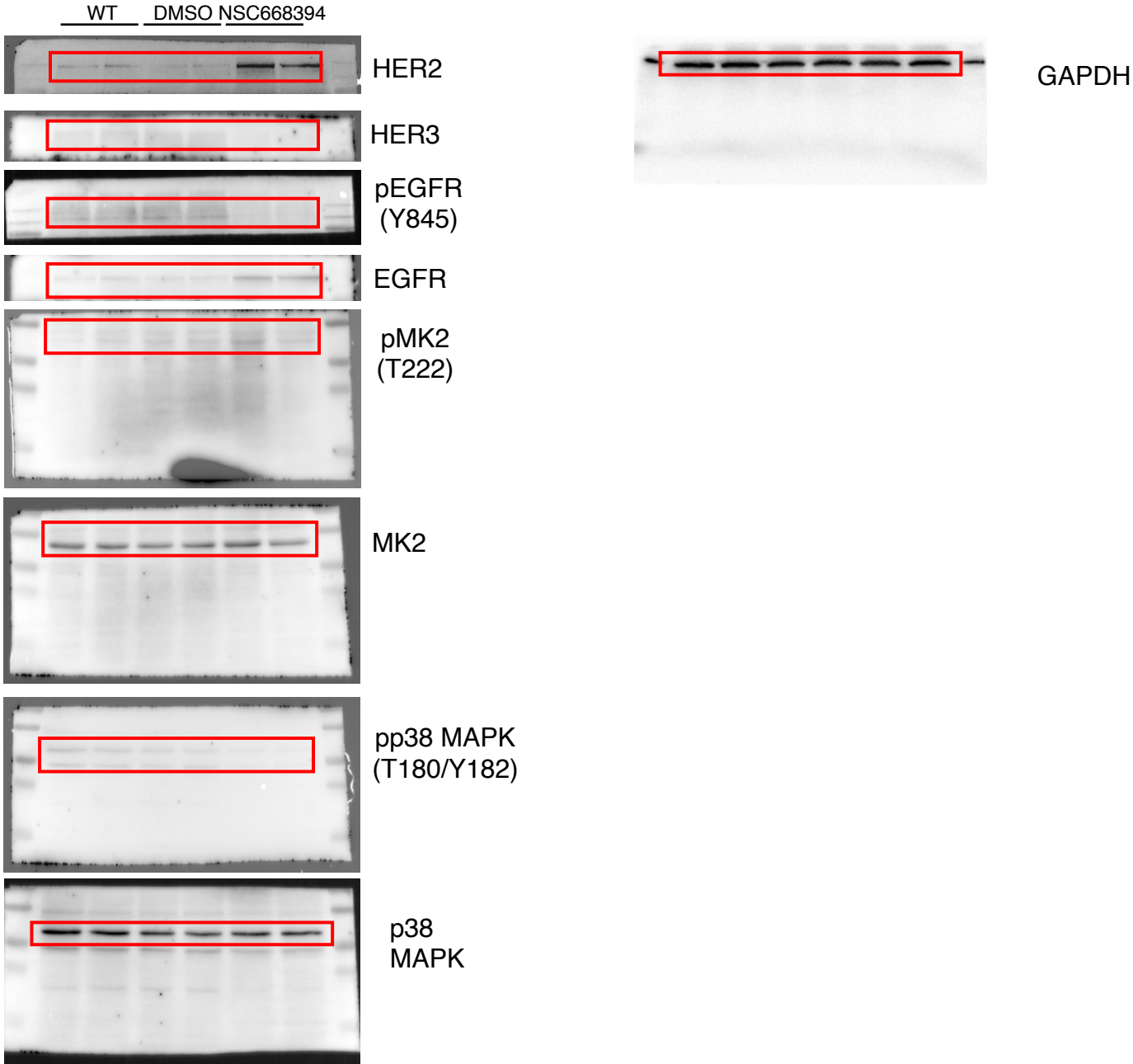

Supplement: Figure 5—figure supplement 1—source data 1. [file elife-98523-fig5-figsupp1-data1.zip › Figure suppl 3/Figure suppl. 3.pdf]

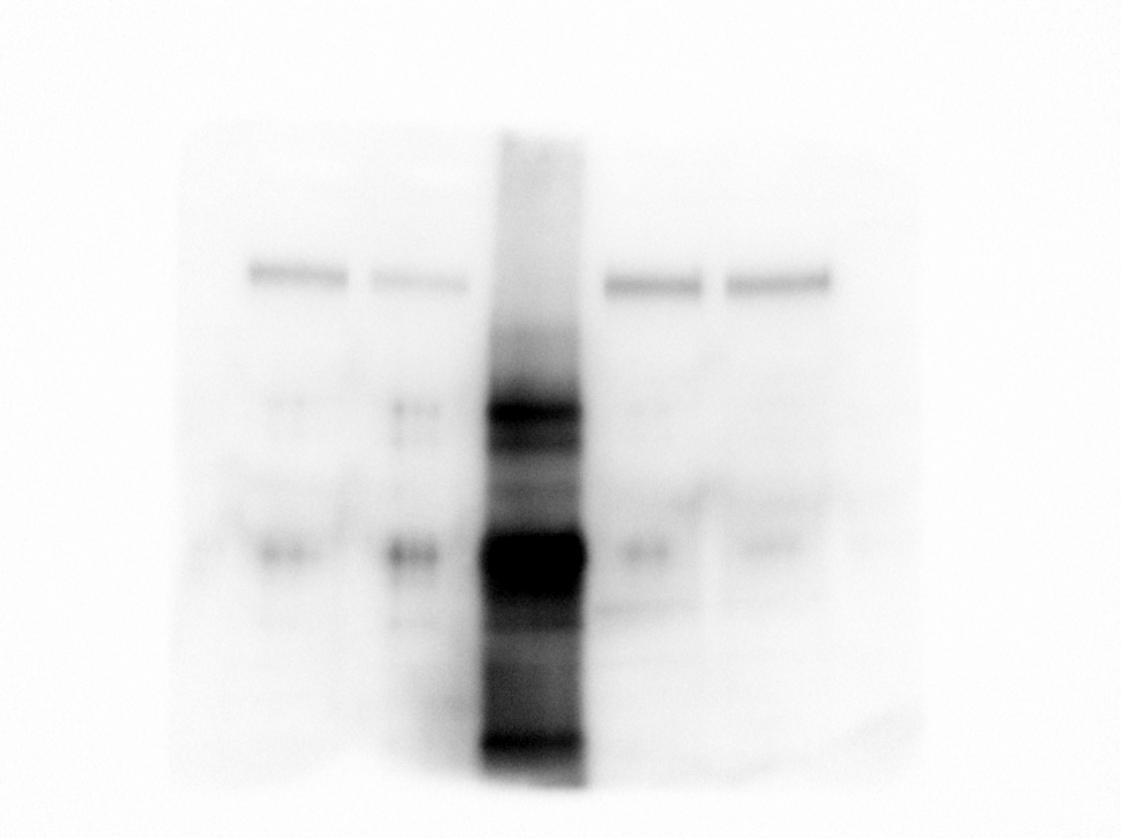

Supplement: Figure 6—source data 1. [file elife-98523-fig6-data1.zip › Figure 6/IP X EGFR-WB X EGFR.tif]

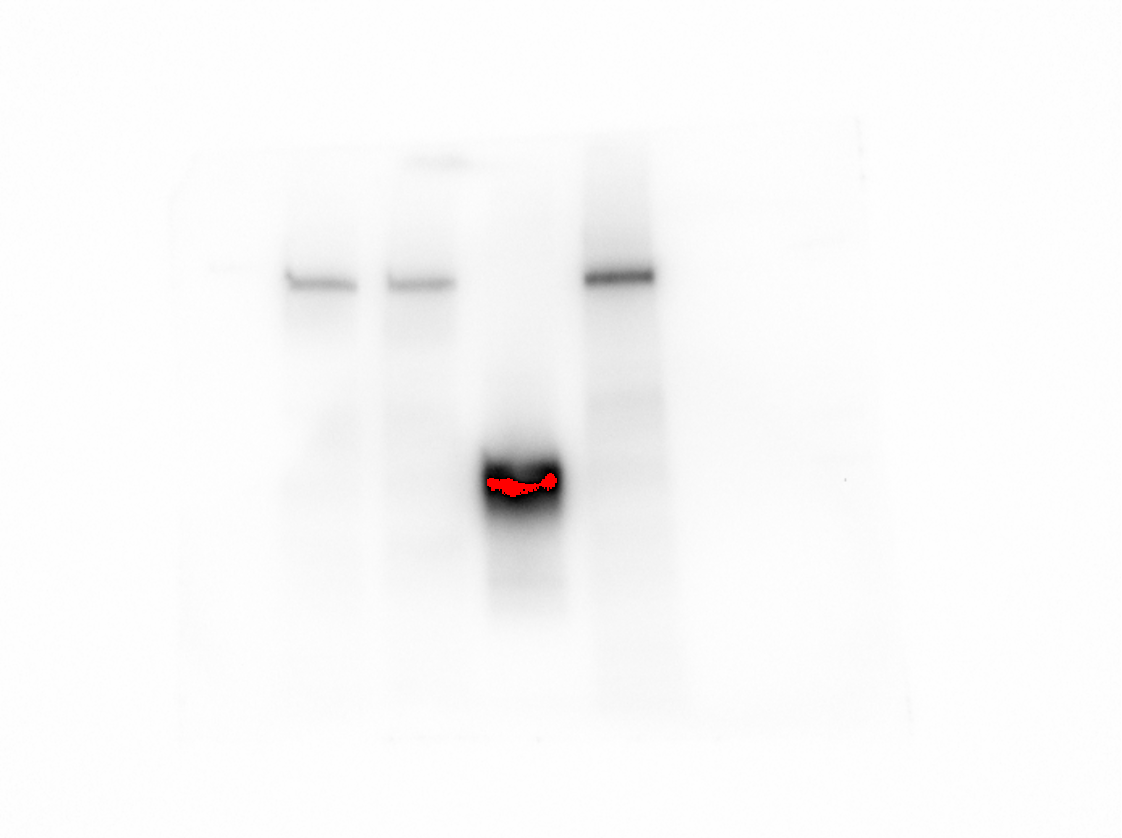

Supplement: Figure 6—source data 1. [file elife-98523-fig6-data1.zip › Figure 6/IP X EZR- WB X TSC2 1.tif]

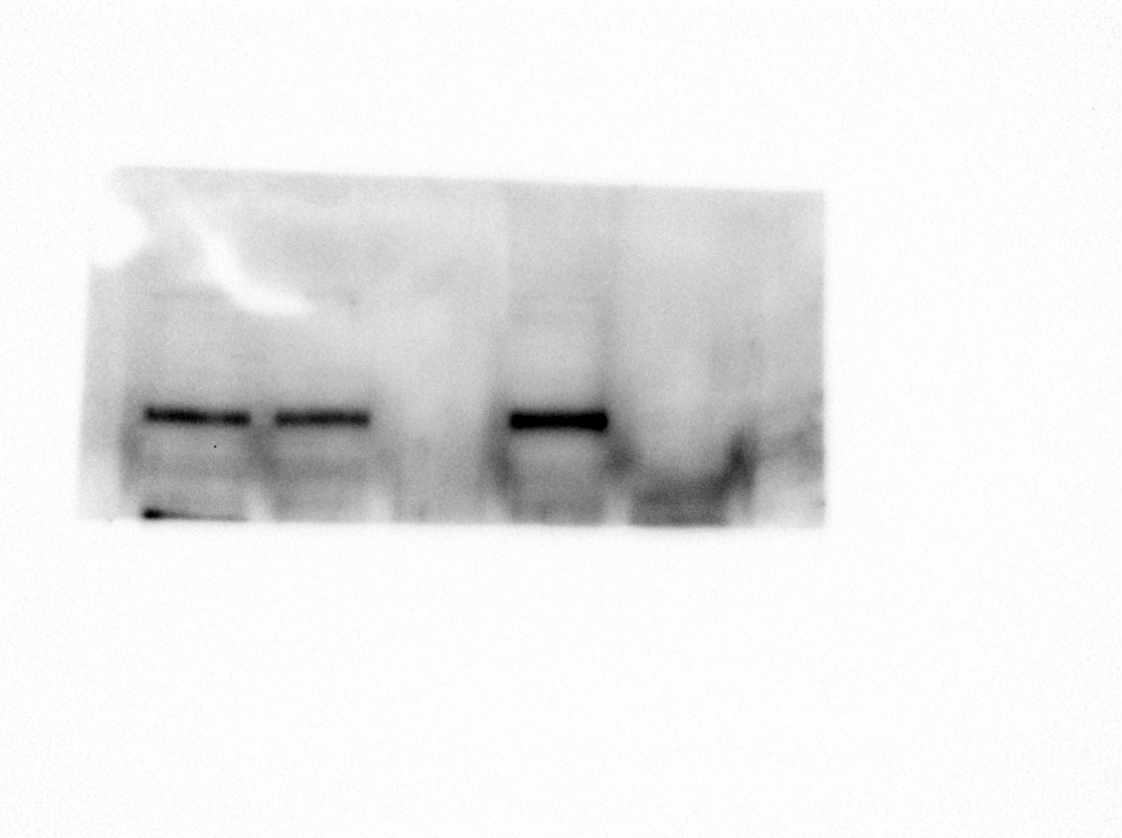

Supplement: Figure 6—source data 1. [file elife-98523-fig6-data1.zip › Figure 6/IP X EGFR-WB X TSC1.tif]

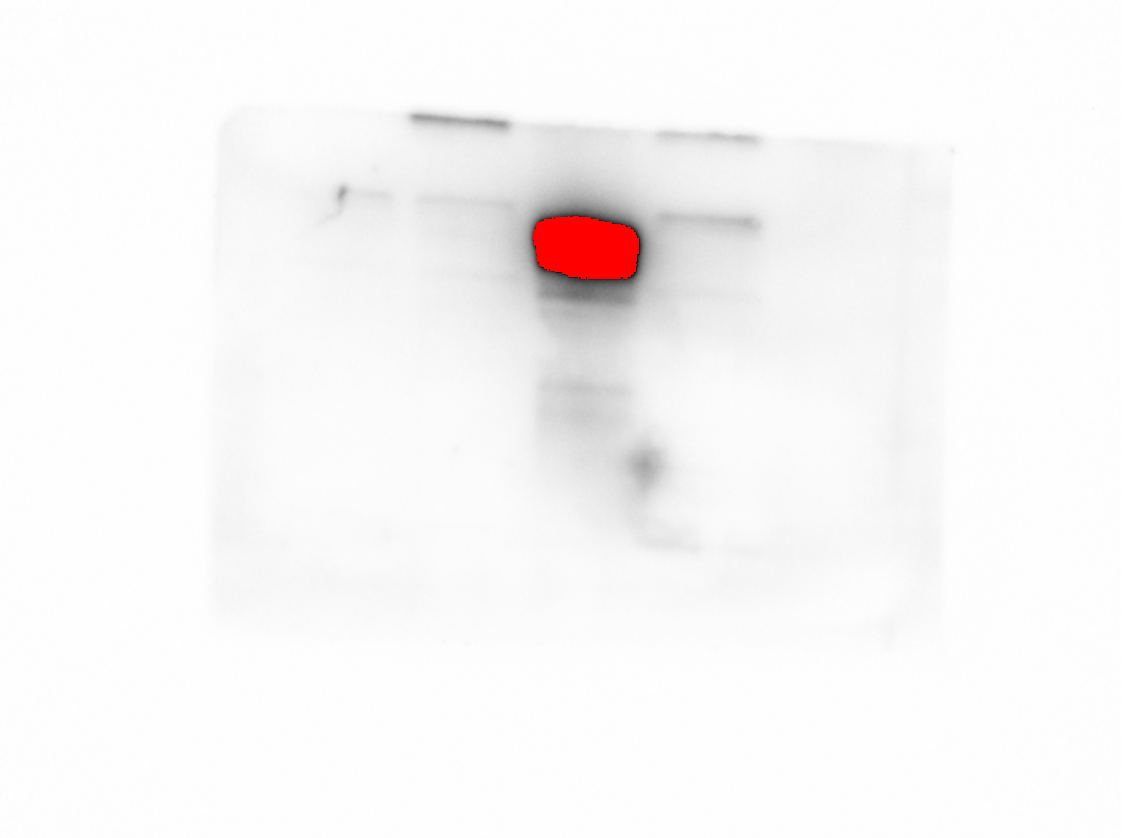

Supplement: Figure 6—source data 1. [file elife-98523-fig6-data1.zip › Figure 6/IP X EZR- WB X AKT.tif]

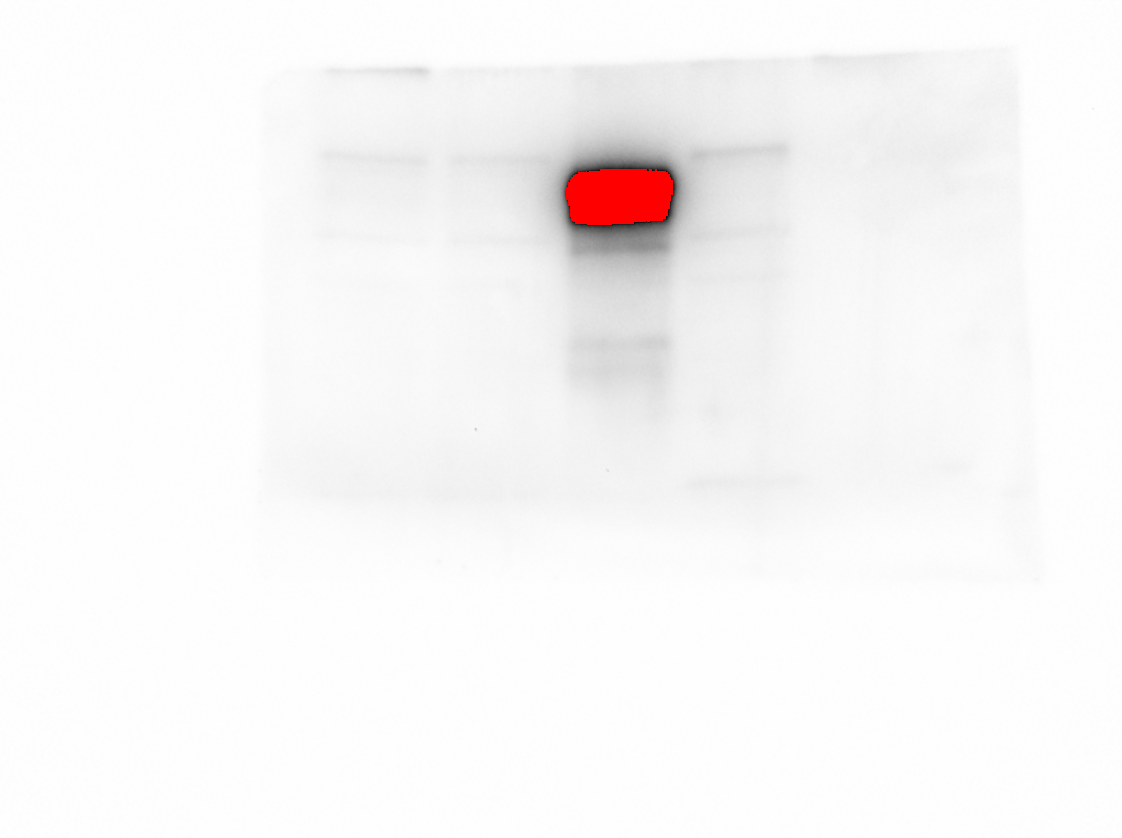

Supplement: Figure 6—source data 1. [file elife-98523-fig6-data1.zip › Figure 6/IP X EGFR- WB X AKT.tif]

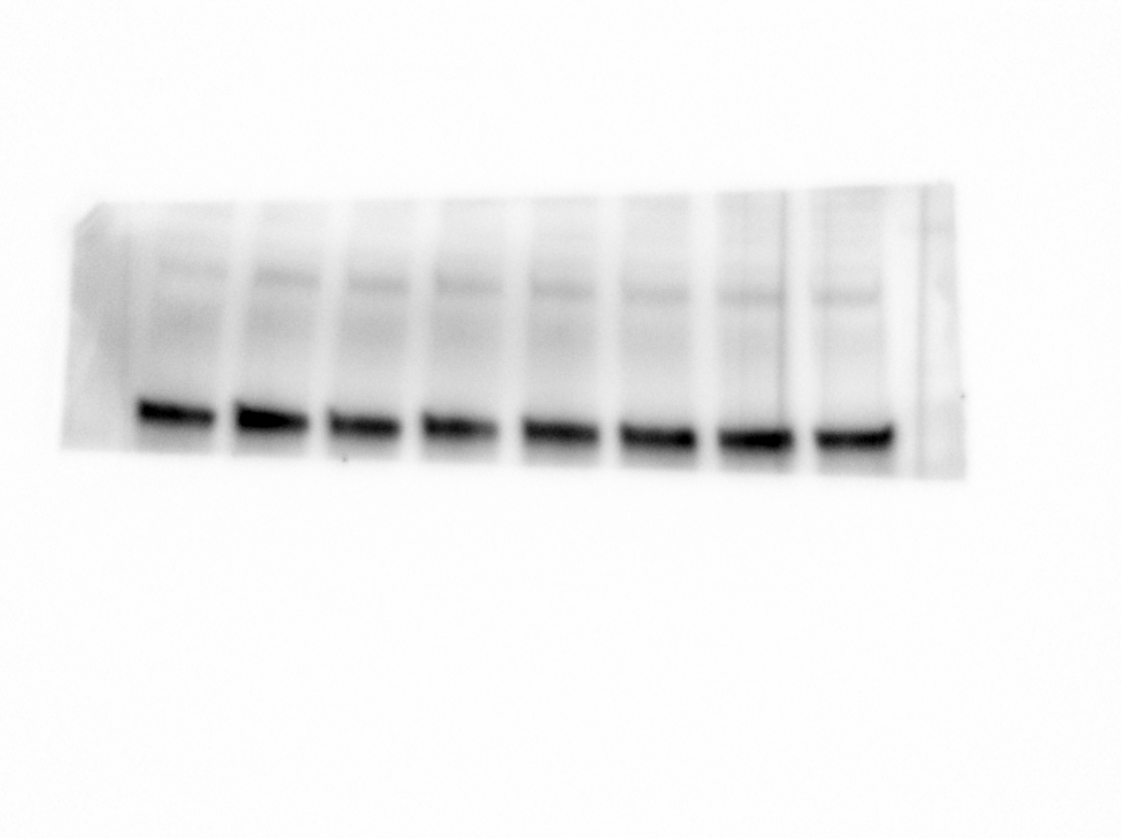

Supplement: Figure 6—source data 1. [file elife-98523-fig6-data1.zip › Figure 6/WT INSULIN P70.tif]

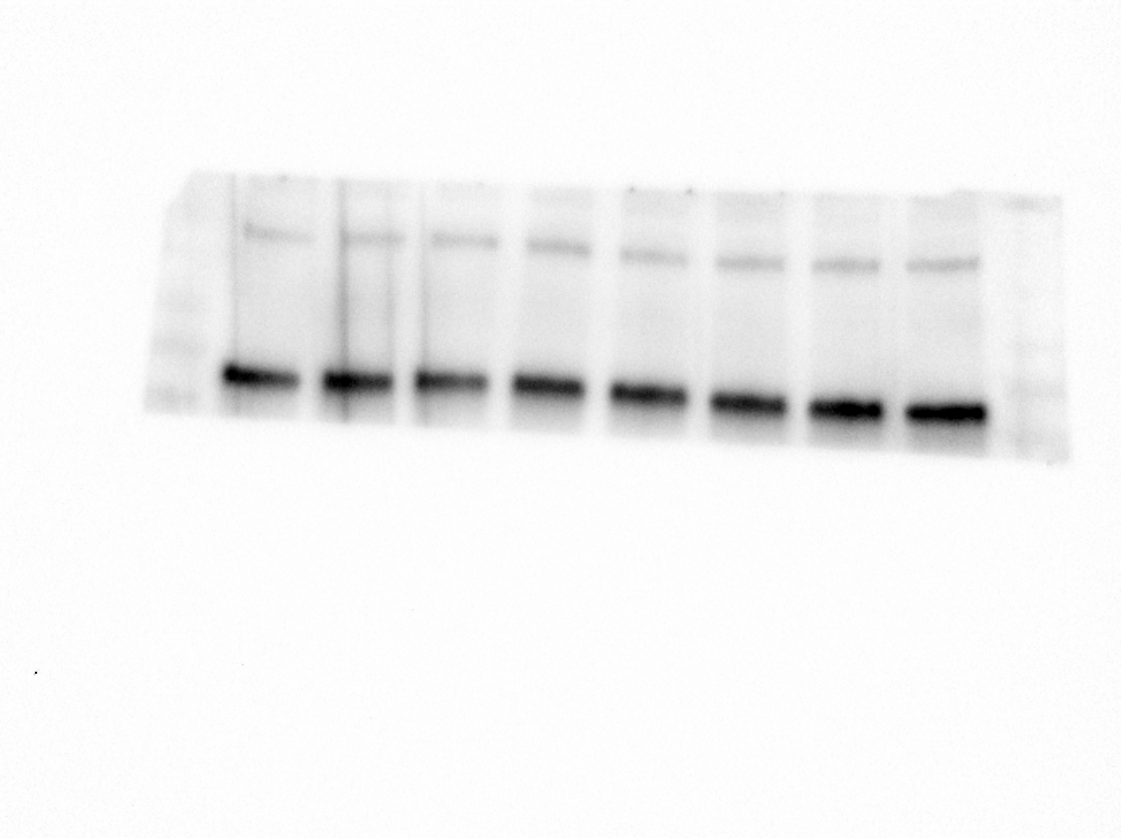

Supplement: Figure 6—source data 1. [file elife-98523-fig6-data1.zip › Figure 6/KO INSULIN P70.tif]

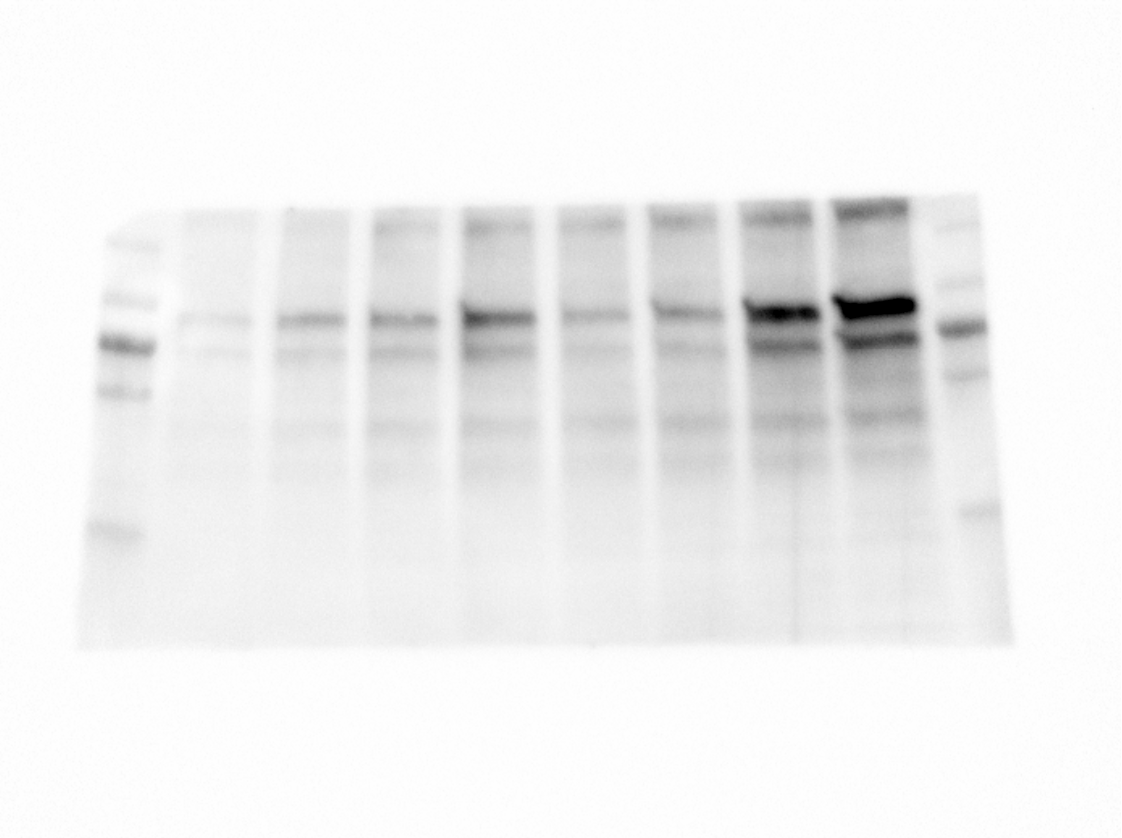

Supplement: Figure 6—source data 1. [file elife-98523-fig6-data1.zip › Figure 6/WT INSULIN pP70.tif]

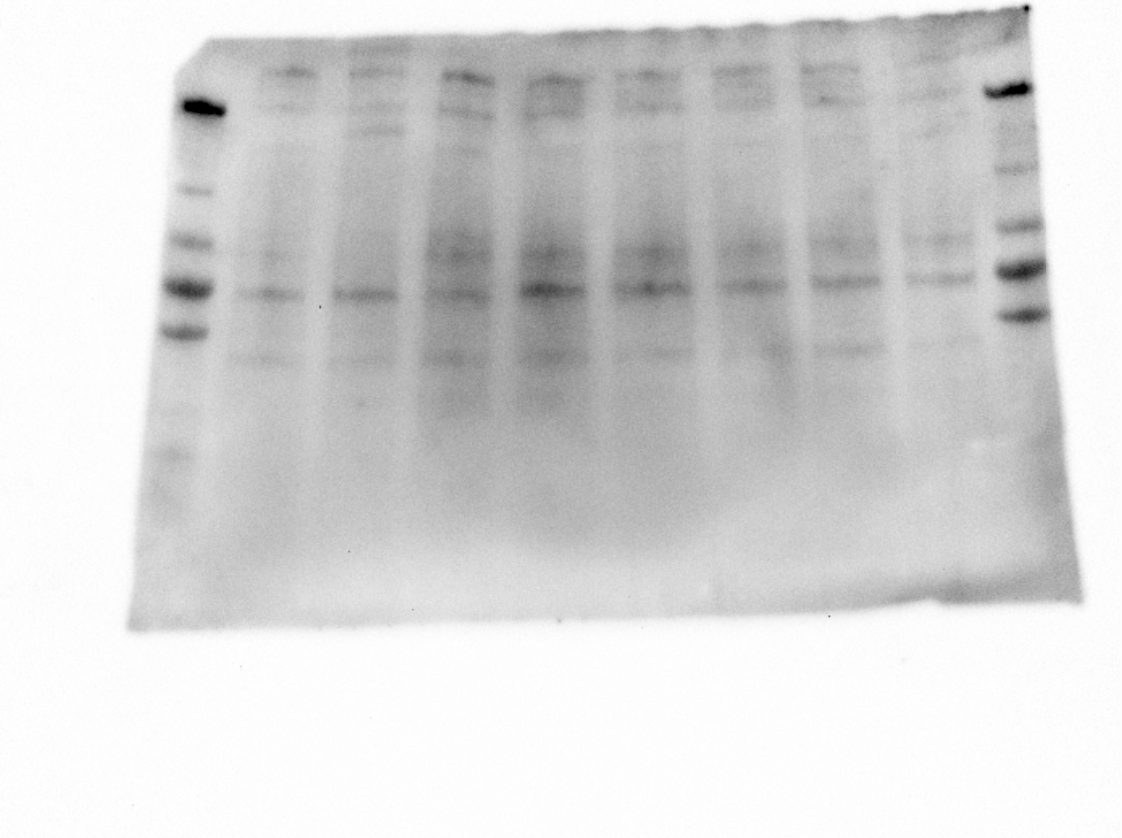

Supplement: Figure 6—source data 1. [file elife-98523-fig6-data1.zip › Figure 6/KO INSULIN pP70.tif]

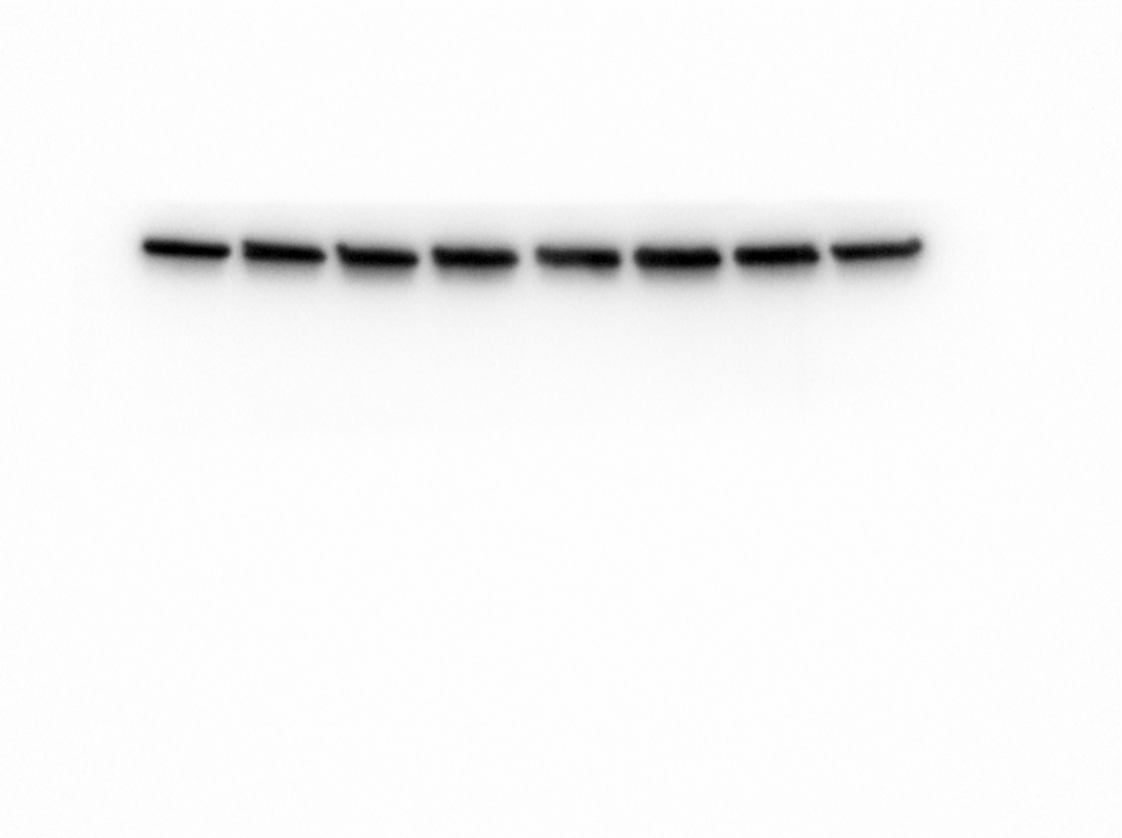

Supplement: Figure 6—source data 1. [file elife-98523-fig6-data1.zip › Figure 6/WT INSULIN GAPDH.tif]

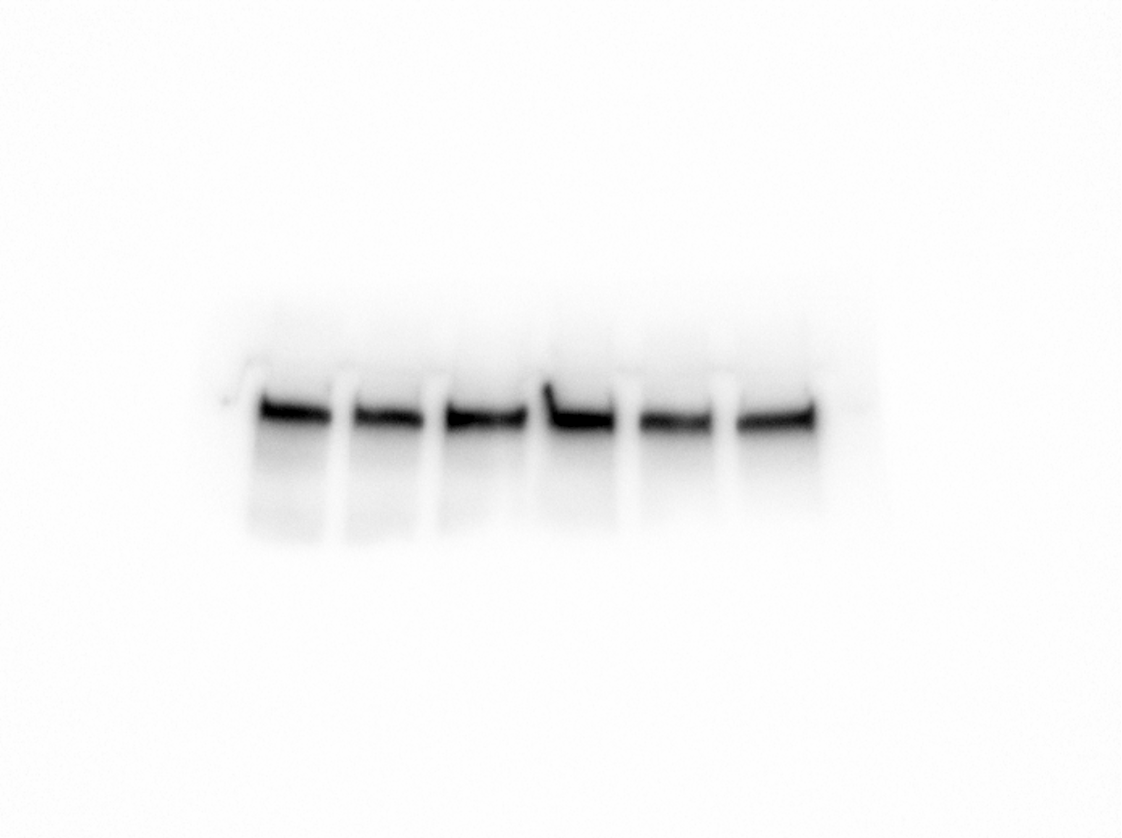

Supplement: Figure 6—source data 1. [file elife-98523-fig6-data1.zip › Figure 6/HELA WT KO TSC2.tif]

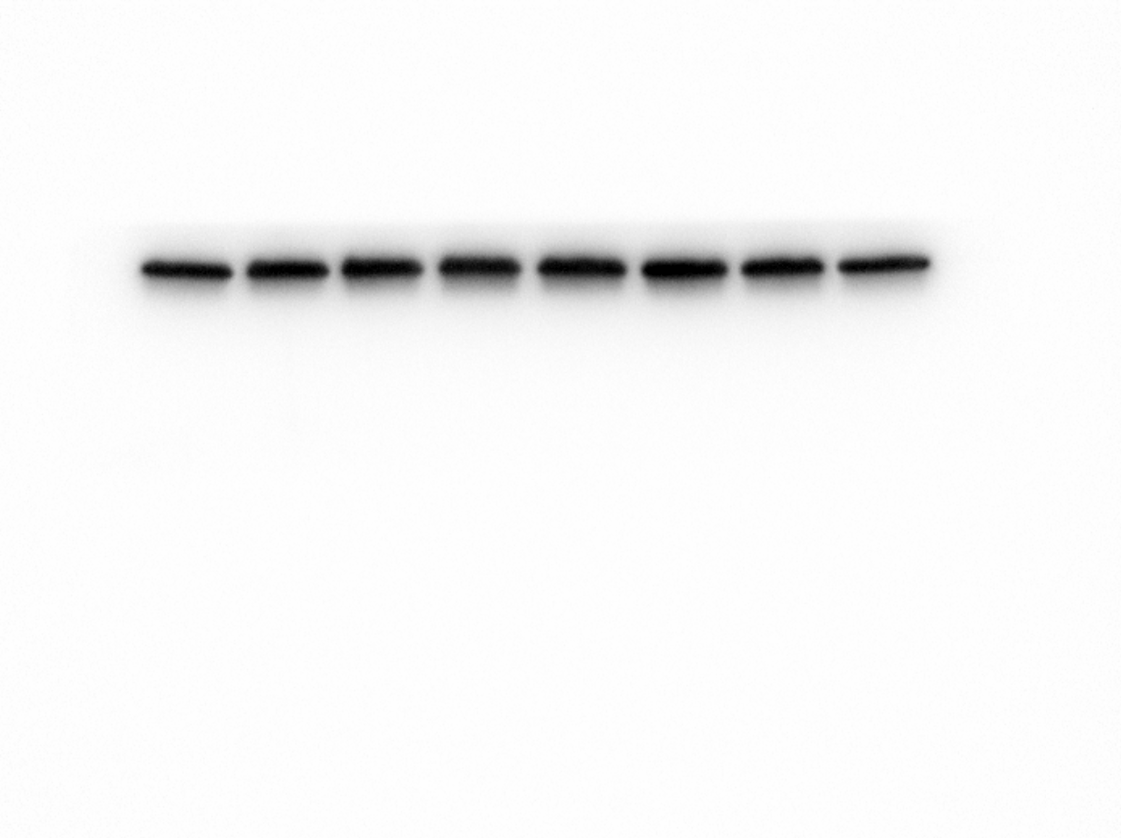

Supplement: Figure 6—source data 1. [file elife-98523-fig6-data1.zip › Figure 6/KO INSULIN GAPDH.tif]

### Figure 6 a

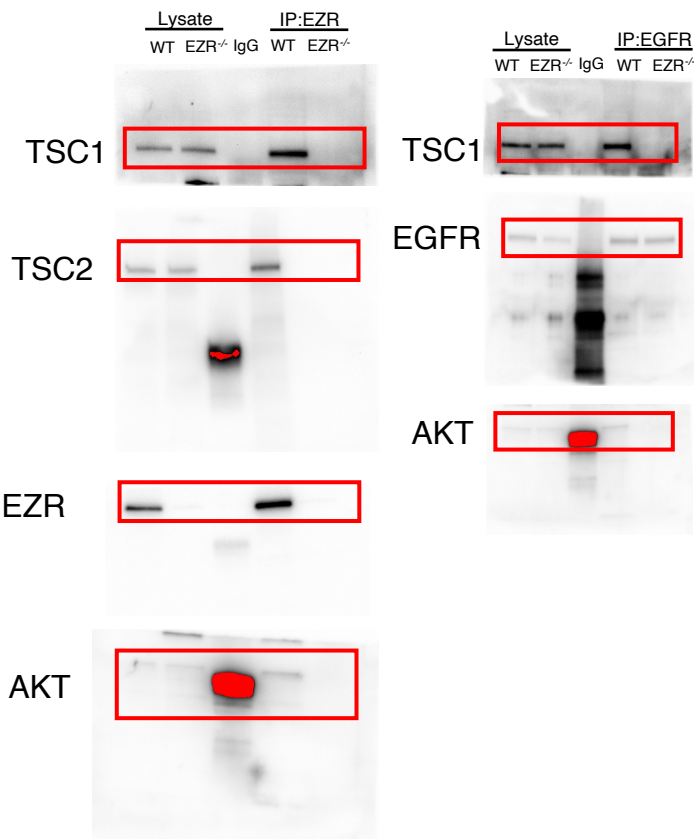

### Figure 6 b

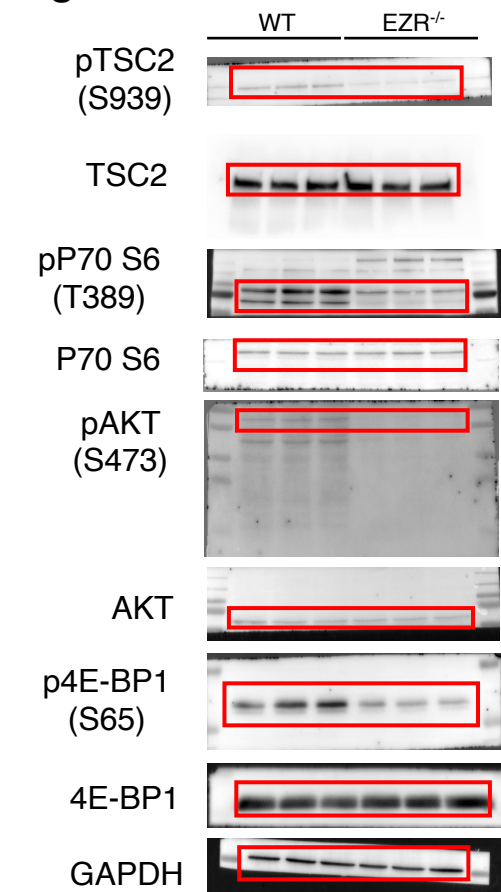

### Figure 6 c

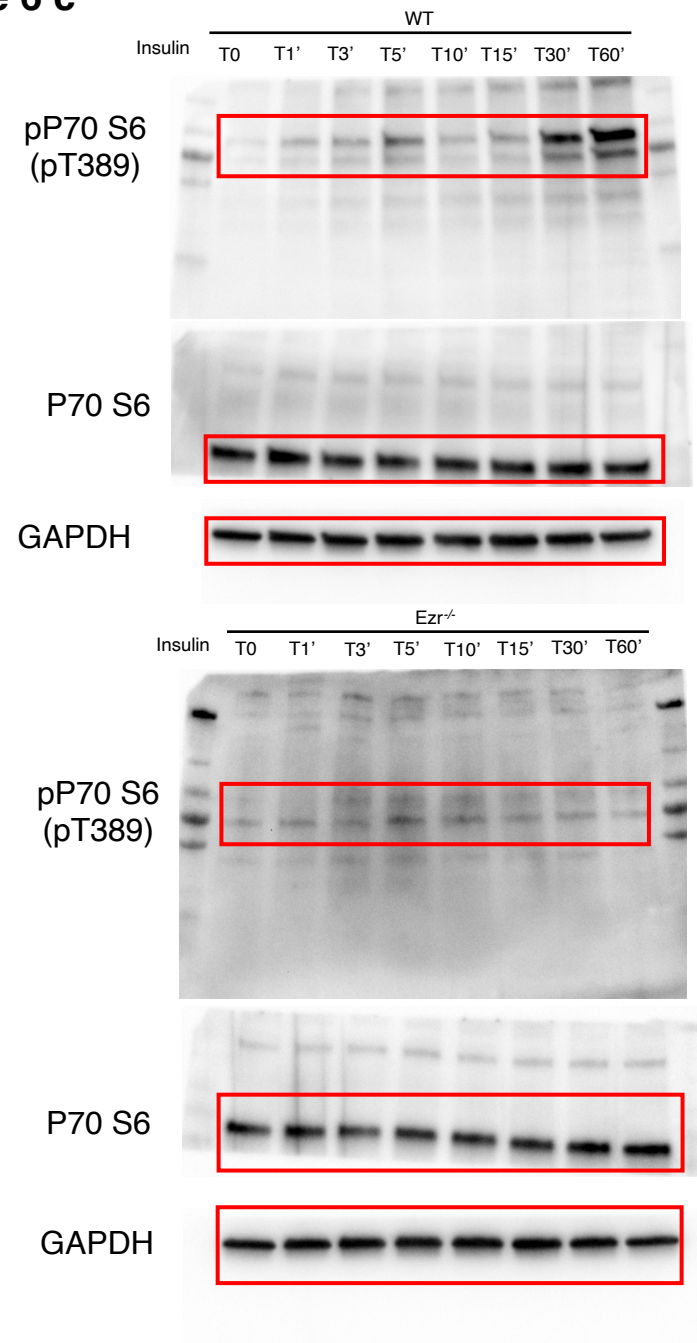

### Figure 6 f

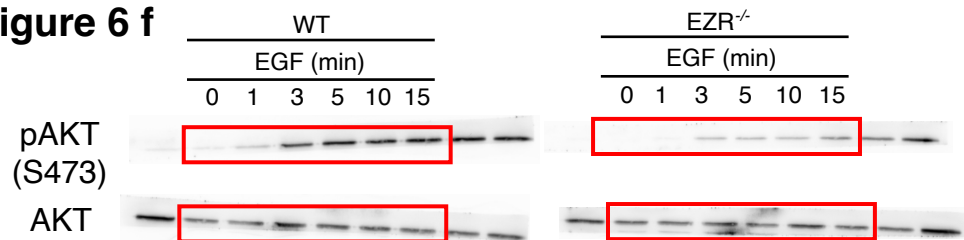

Supplement: Figure 6—source data 1. [file elife-98523-fig6-data1.zip › Figure 6/Figure 6.pdf]

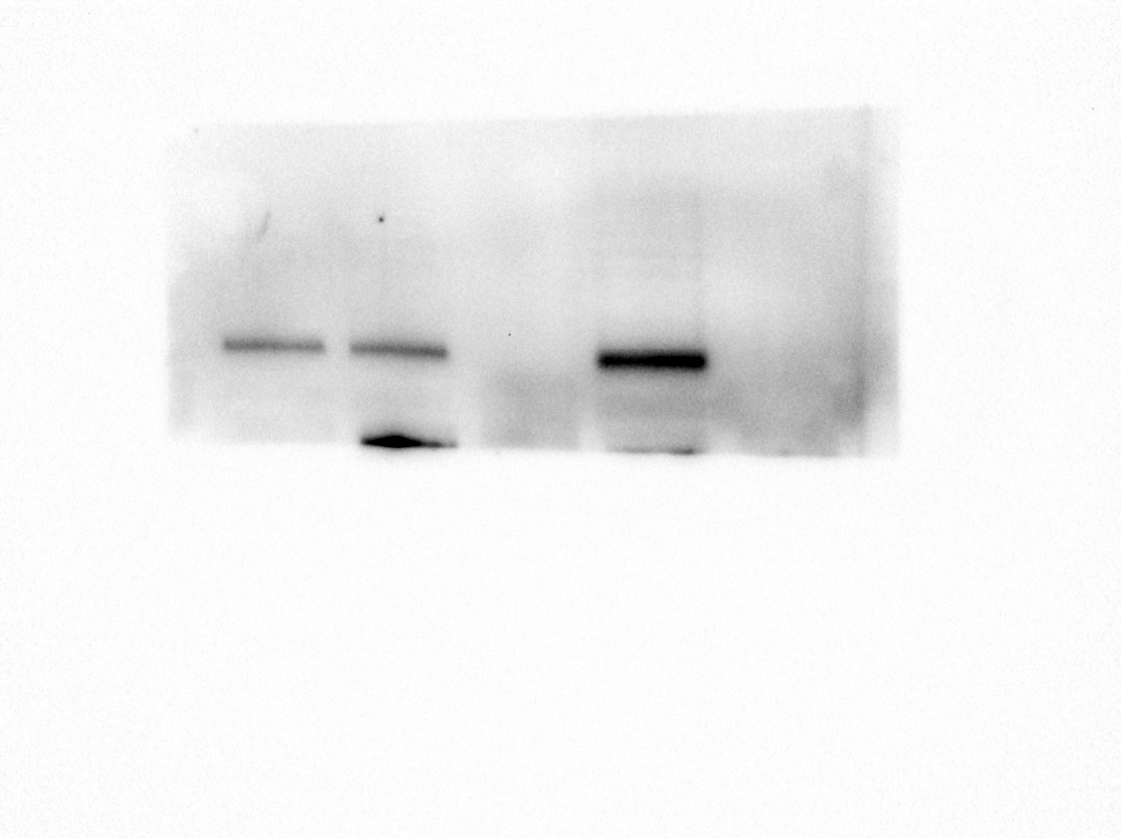

Supplement: Figure 6—source data 1. [file elife-98523-fig6-data1.zip › Figure 6/IP X EZR- WB X TSC1 1.tif]

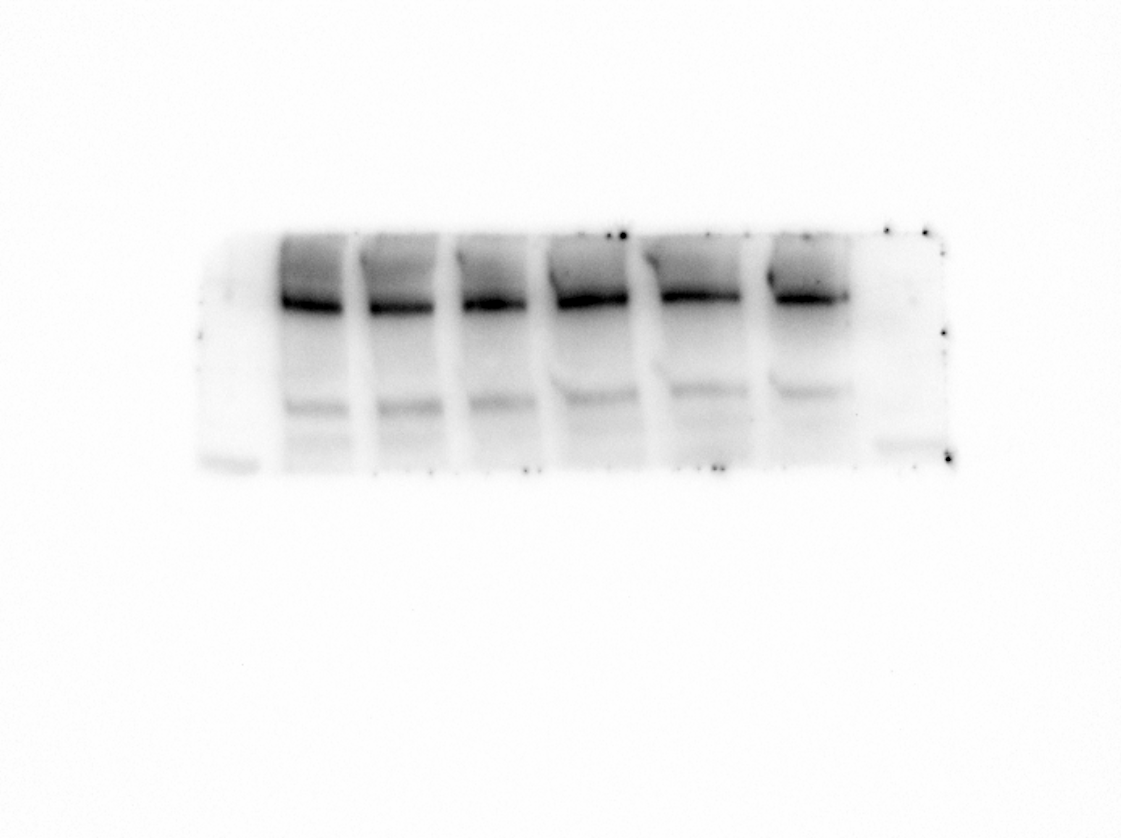

Supplement: Figure 6—figure supplement 1—source data 1. [file elife-98523-fig6-figsupp1-data1.zip › Figure suppl 4/MEF WT KO TSC2.tif]

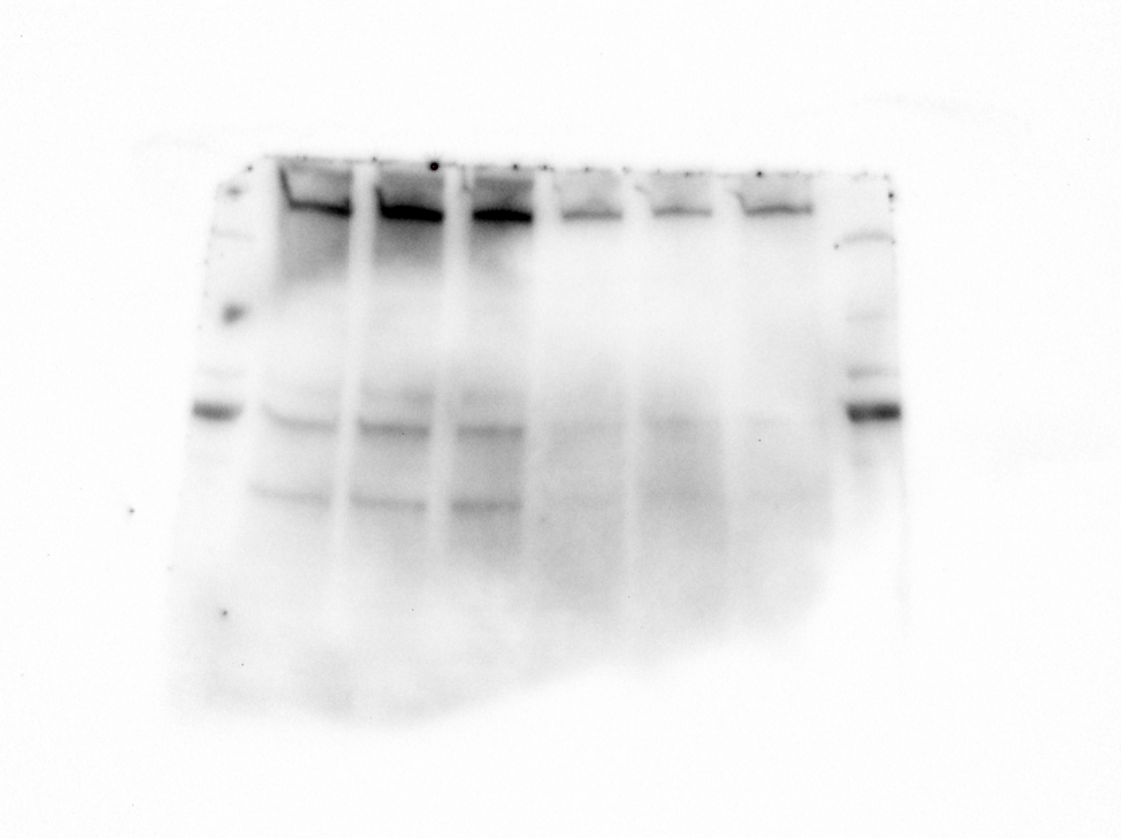

Supplement: Figure 6—figure supplement 1—source data 1. [file elife-98523-fig6-figsupp1-data1.zip › Figure suppl 4/MEF WT KO pP70.tif]

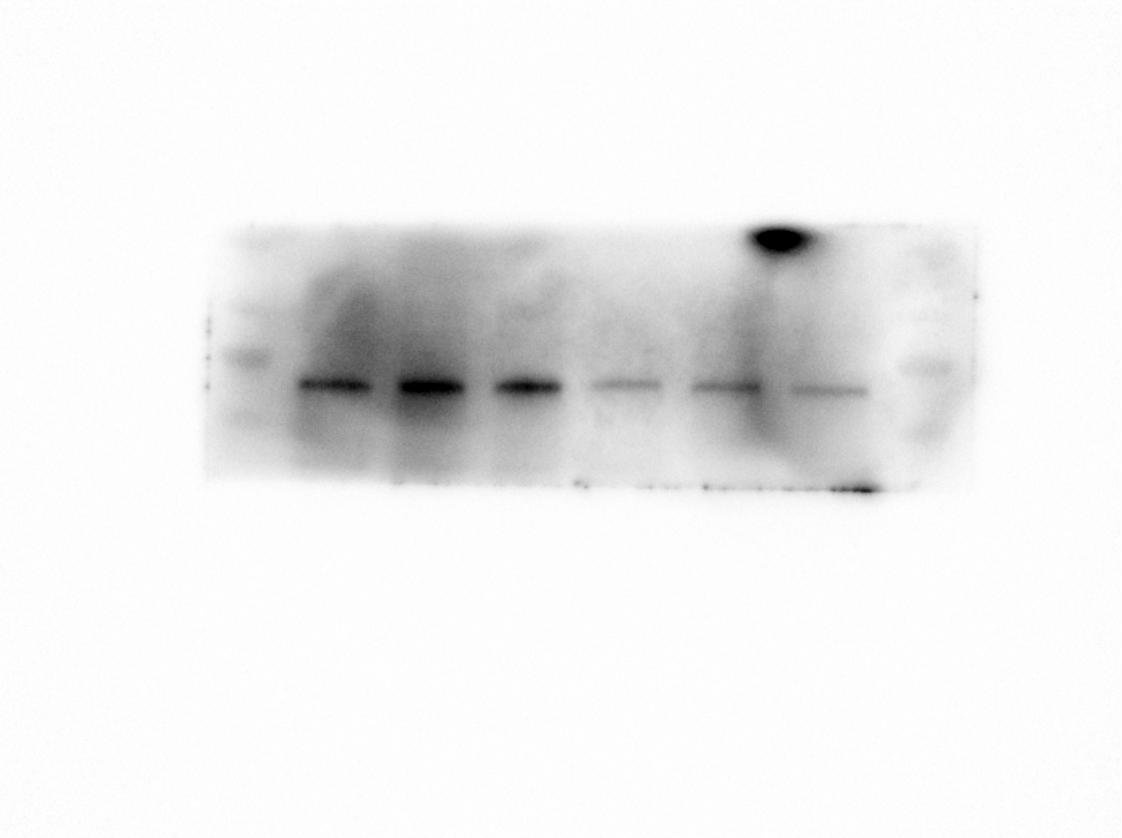

Supplement: Figure 6—figure supplement 1—source data 1. [file elife-98523-fig6-figsupp1-data1.zip › Figure suppl 4/MEF WT KO pAKT.tif]

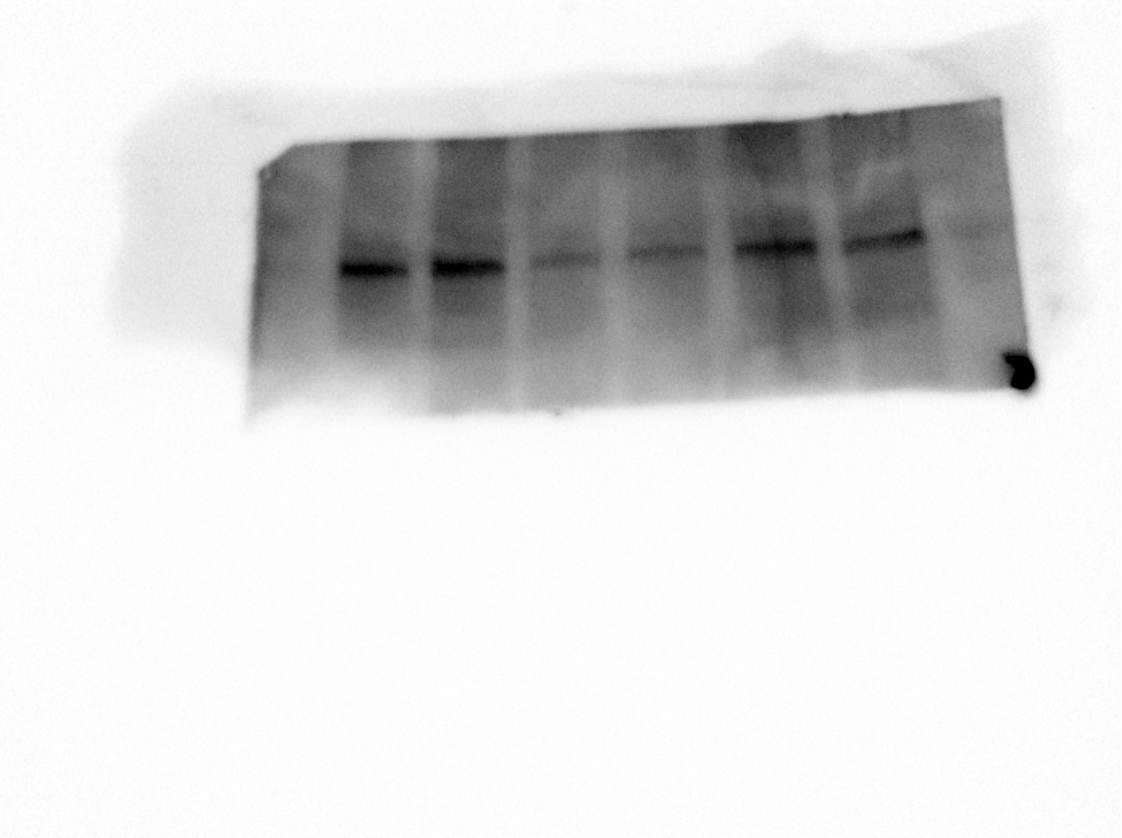

Supplement: Figure 6—figure supplement 1—source data 1. [file elife-98523-fig6-figsupp1-data1.zip › Figure suppl 4/MEF WT DR TSC2 pP70.tif]

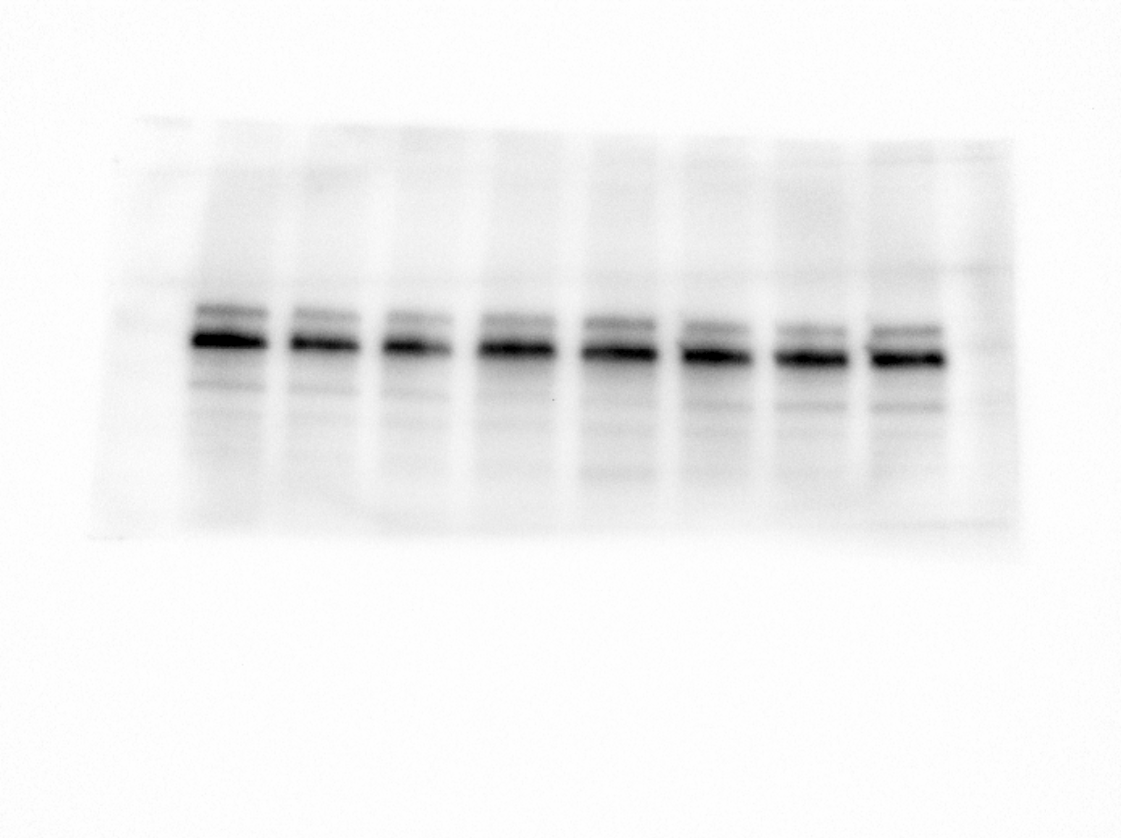

Supplement: Figure 6—figure supplement 1—source data 1. [file elife-98523-fig6-figsupp1-data1.zip › Figure suppl 4/MEF WT KO TSC2 P70.tif]

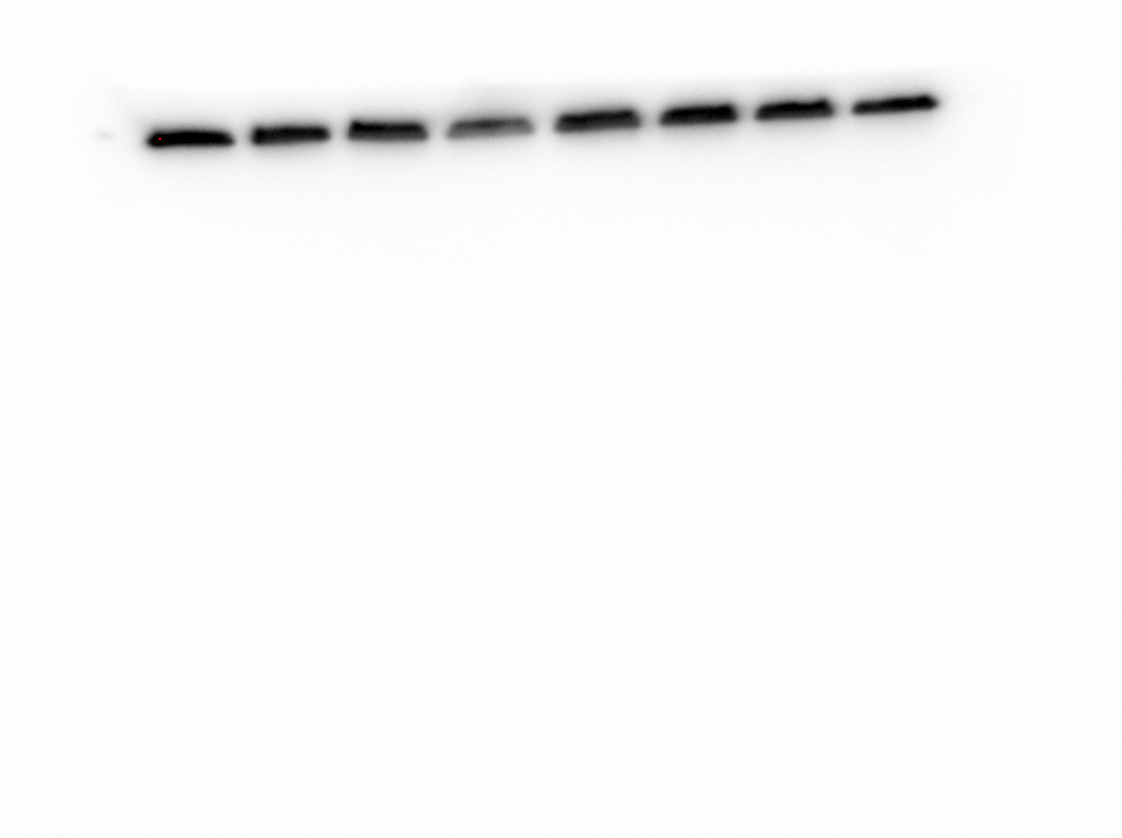

Supplement: Figure 6—figure supplement 1—source data 1. [file elife-98523-fig6-figsupp1-data1.zip › Figure suppl 4/MEF WT DR TSC2 GAPDH.tif]

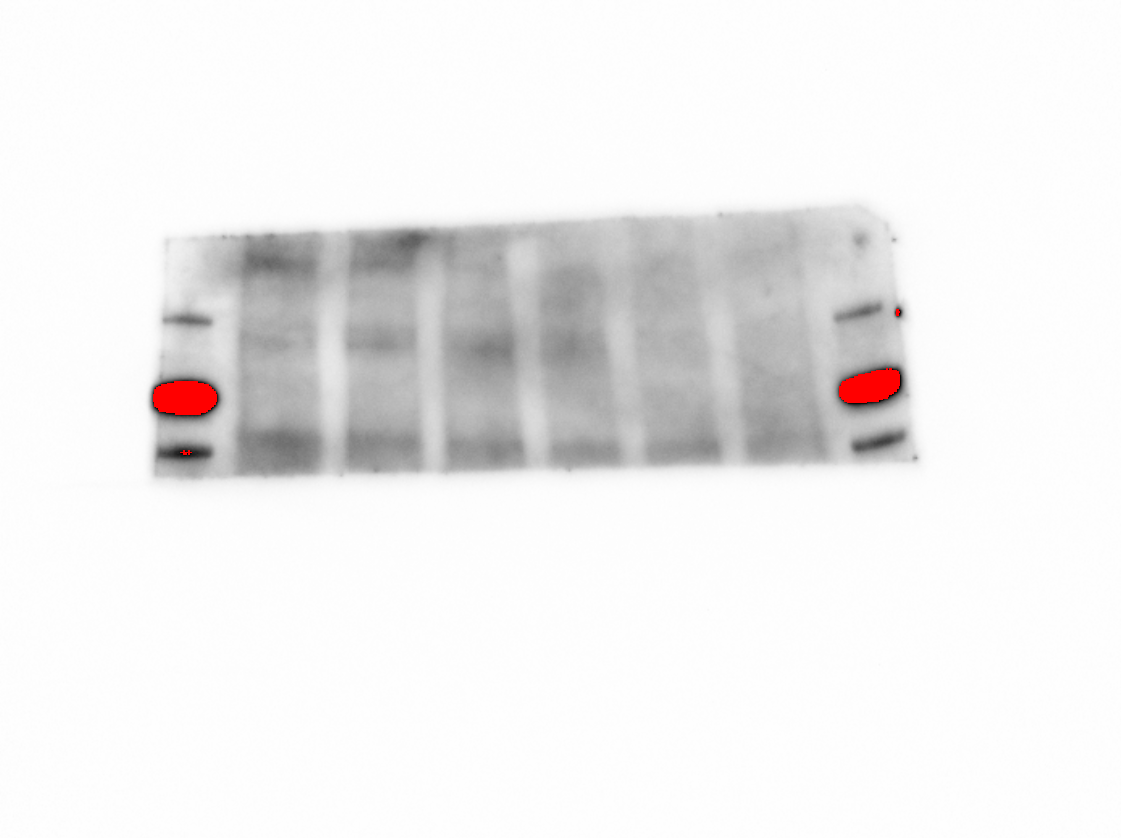

Supplement: Figure 7—source data 1. [file elife-98523-fig7-data1.zip › Figure 7/MEDAKA WT KO pTSC2.tif]

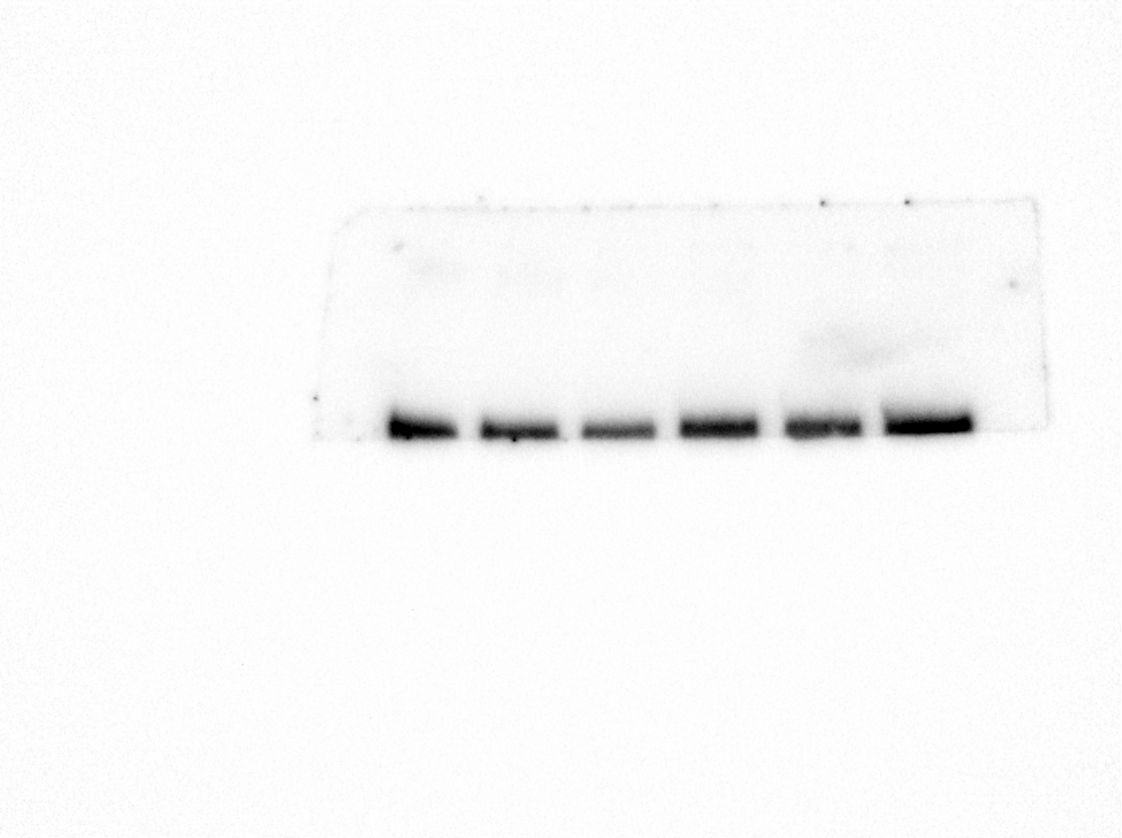

Supplement: Figure 7—source data 1. [file elife-98523-fig7-data1.zip › Figure 7/MEDAKA WT KO TSC2.tif]

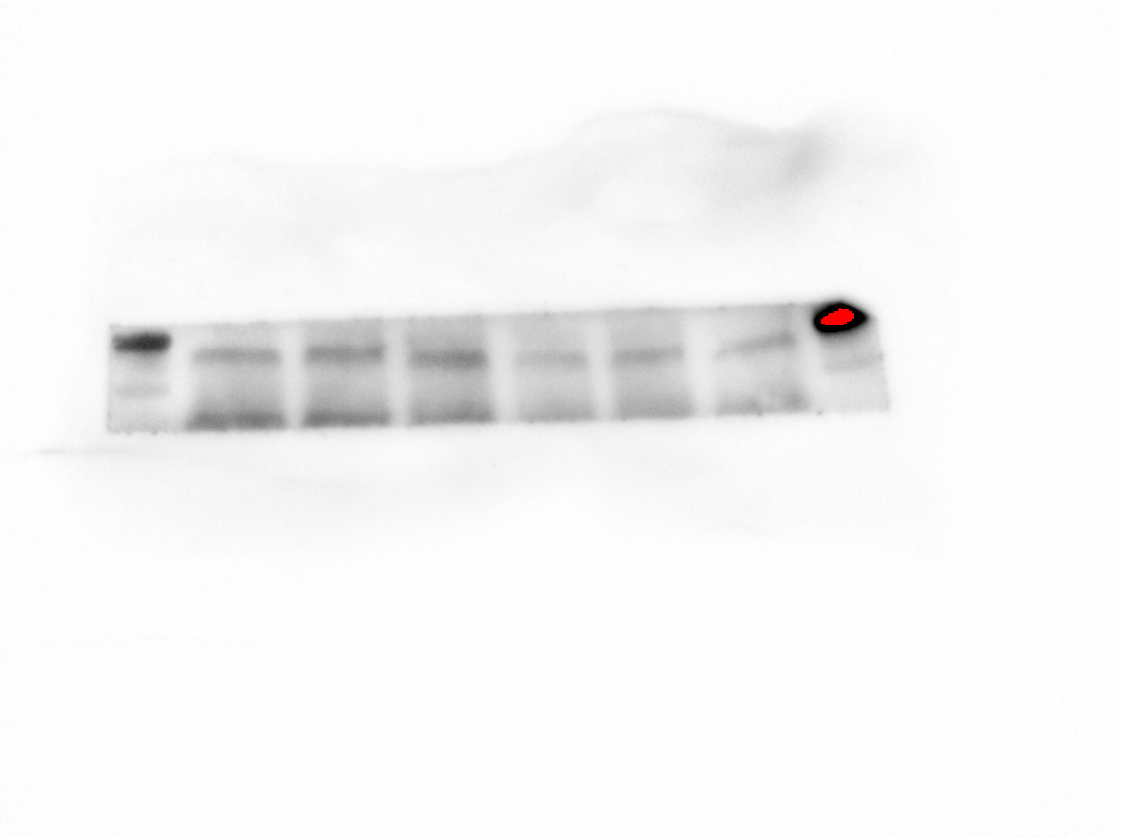

Supplement: Figure 7—source data 1. [file elife-98523-fig7-data1.zip › Figure 7/MEDAKA WT KO pAKT.tif]

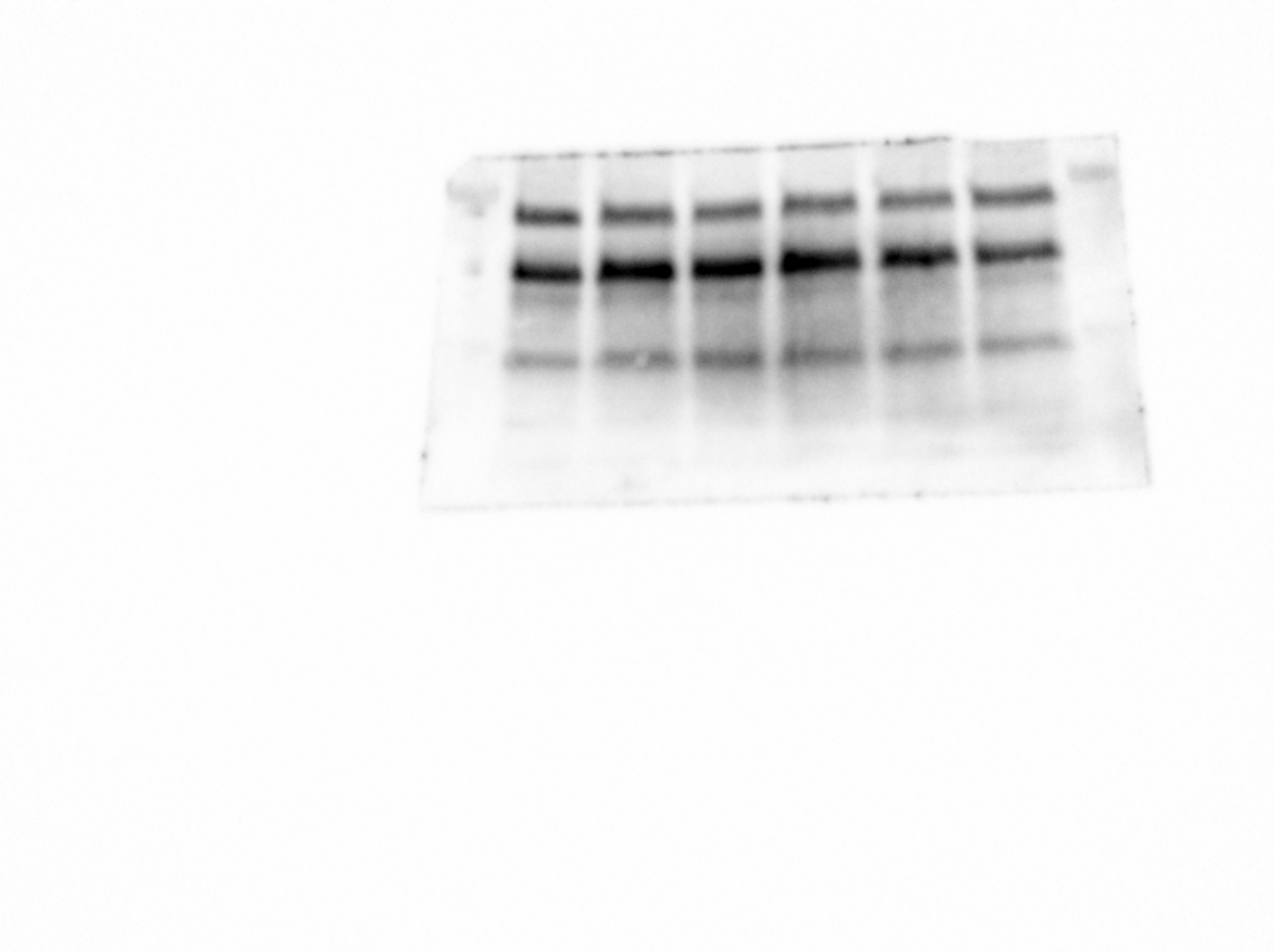

Supplement: Figure 7—source data 1. [file elife-98523-fig7-data1.zip › Figure 7/MEDAKA WT KO AKT.tif]

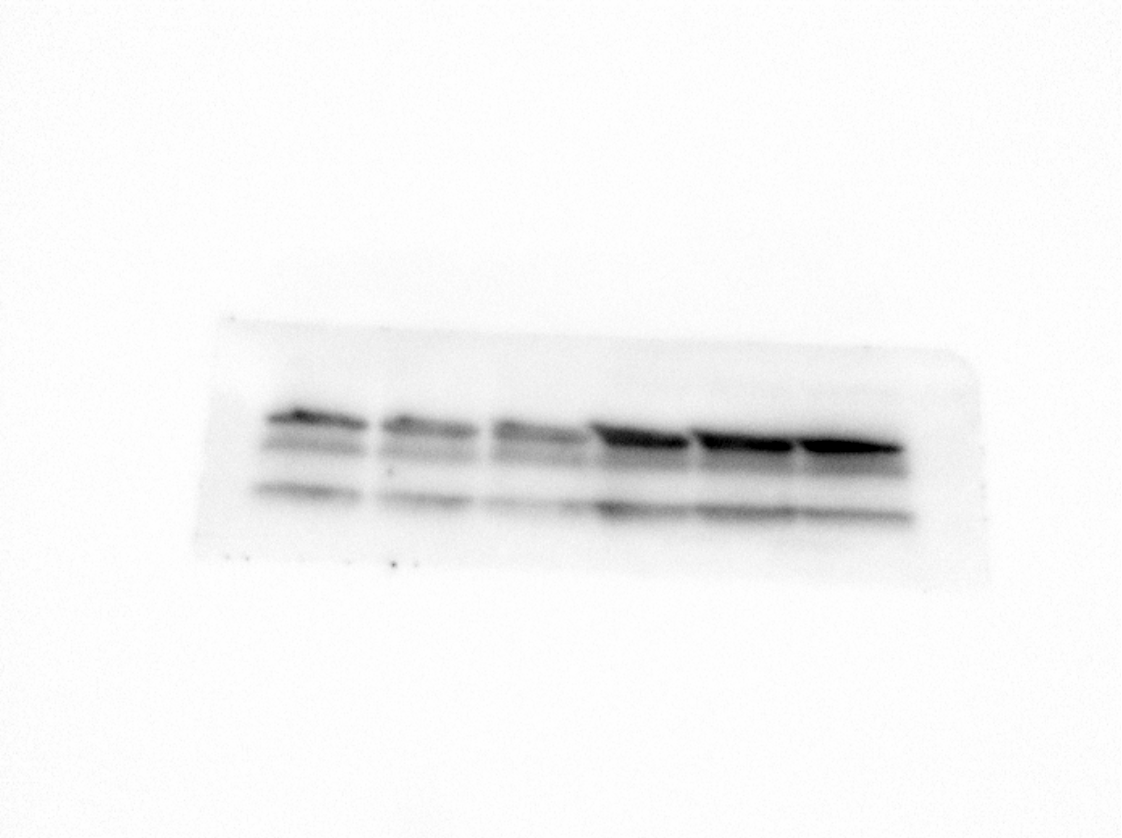

Supplement: Figure 7—source data 1. [file elife-98523-fig7-data1.zip › Figure 7/MEDAKA WT KO LC3.tif]

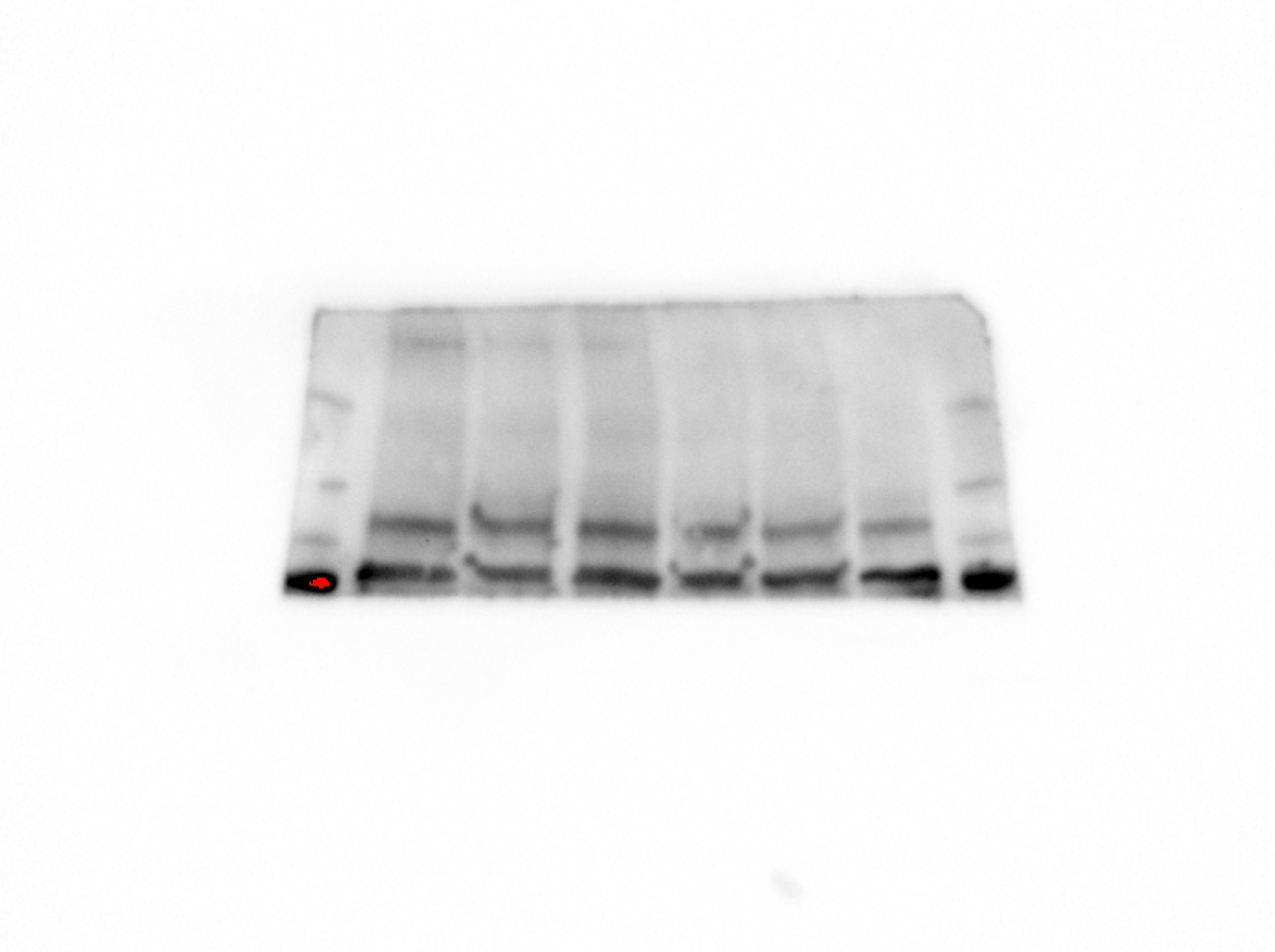

Supplement: Figure 7—source data 1. [file elife-98523-fig7-data1.zip › Figure 7/MEDAKA WT KO pEGFR.tif]

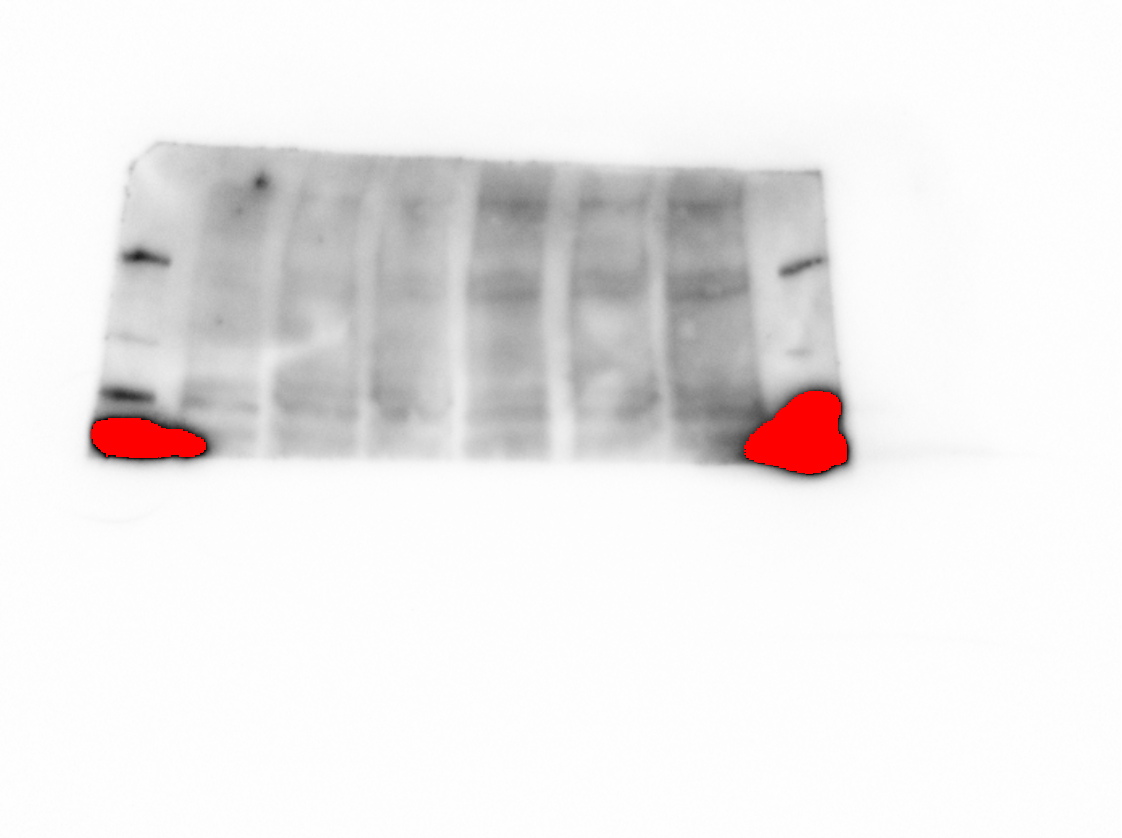

Supplement: Figure 7—source data 1. [file elife-98523-fig7-data1.zip › Figure 7/MEDAKA WT KO EGFR.tif]

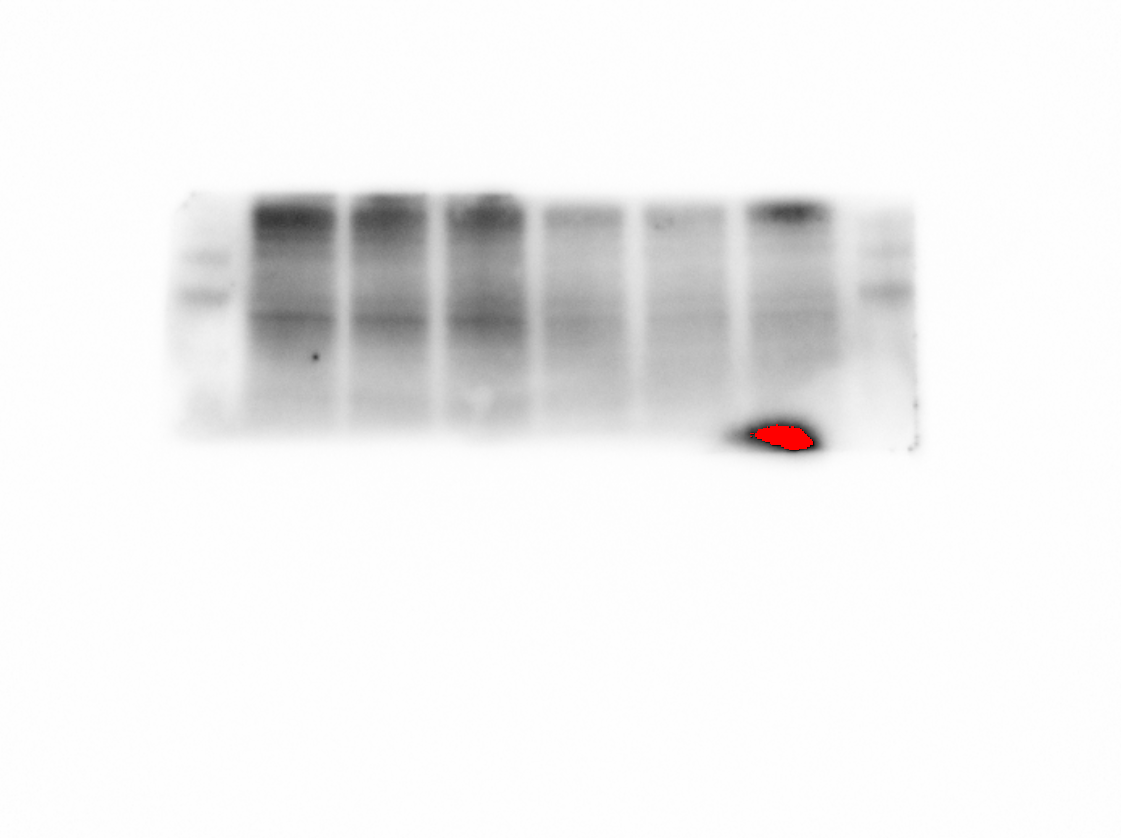

Supplement: Figure 7—source data 1. [file elife-98523-fig7-data1.zip › Figure 7/MEDAKA WT KO p4EBP.tif]

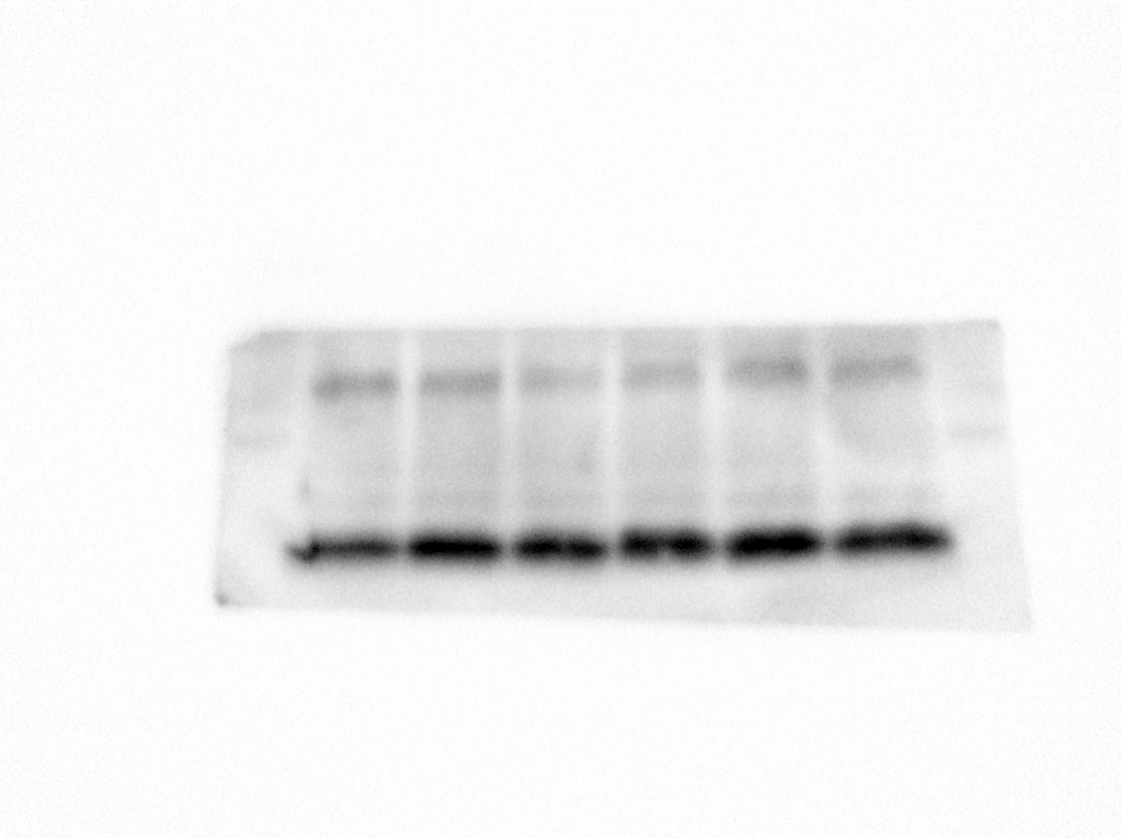

Supplement: Figure 7—source data 1. [file elife-98523-fig7-data1.zip › Figure 7/MEDAKA WT KO 4EBP.tif]

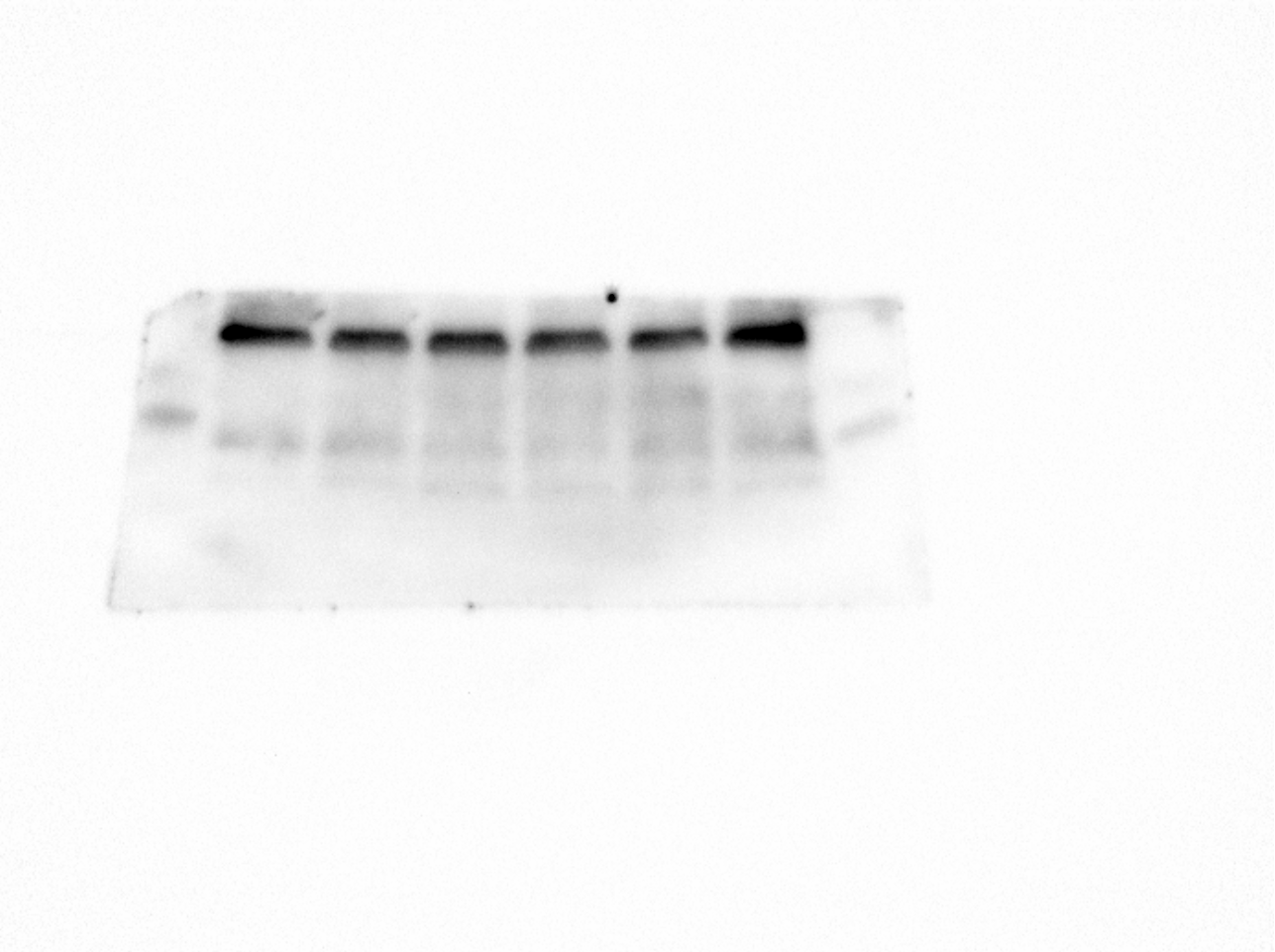

Supplement: Figure 7—source data 1. [file elife-98523-fig7-data1.zip › Figure 7/MEDAKA WT KO ACTIN.tif]

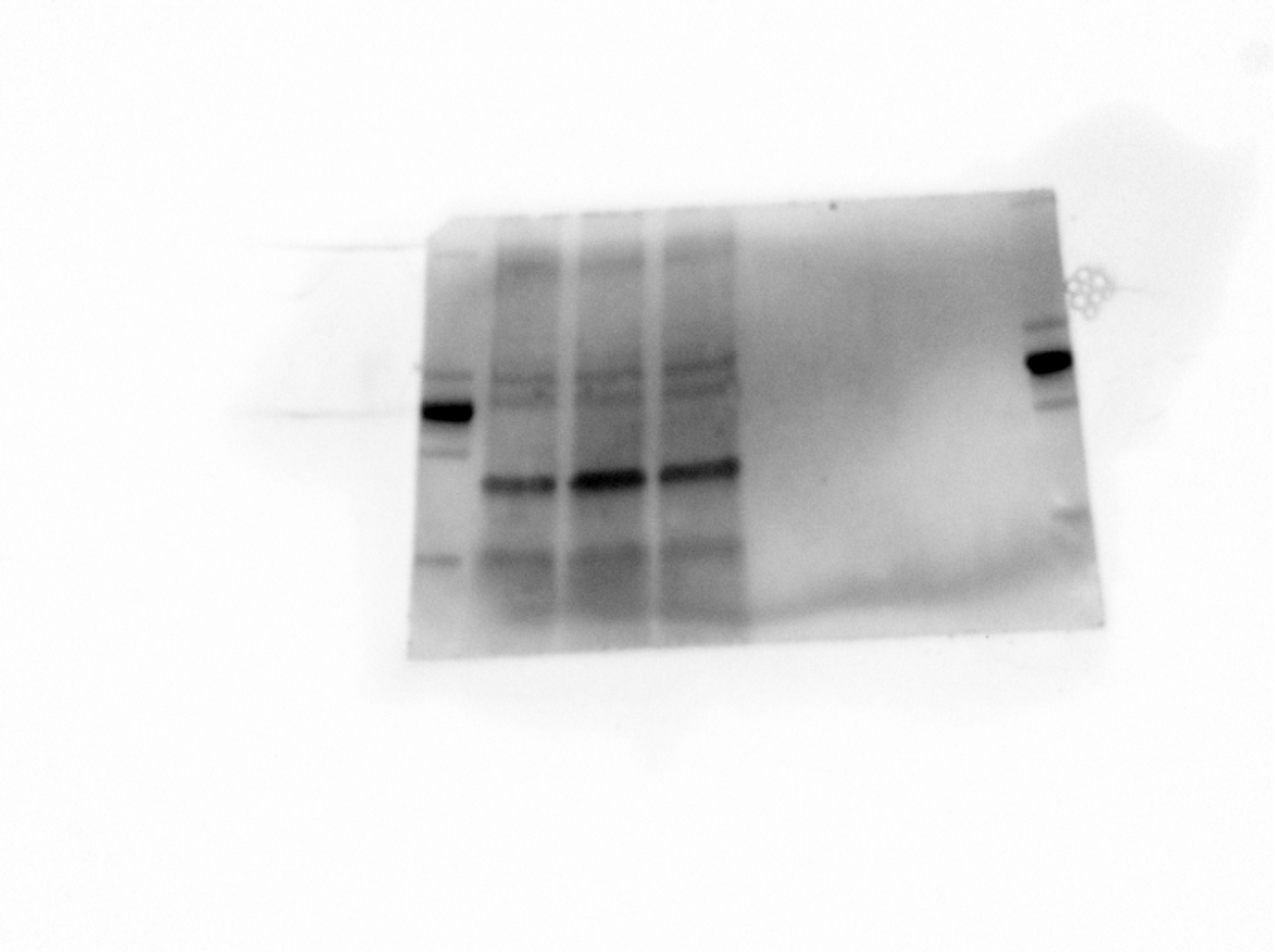

Supplement: Figure 7—source data 1. [file elife-98523-fig7-data1.zip › Figure 7/MEDAKA WT KO EZRIN.tif]

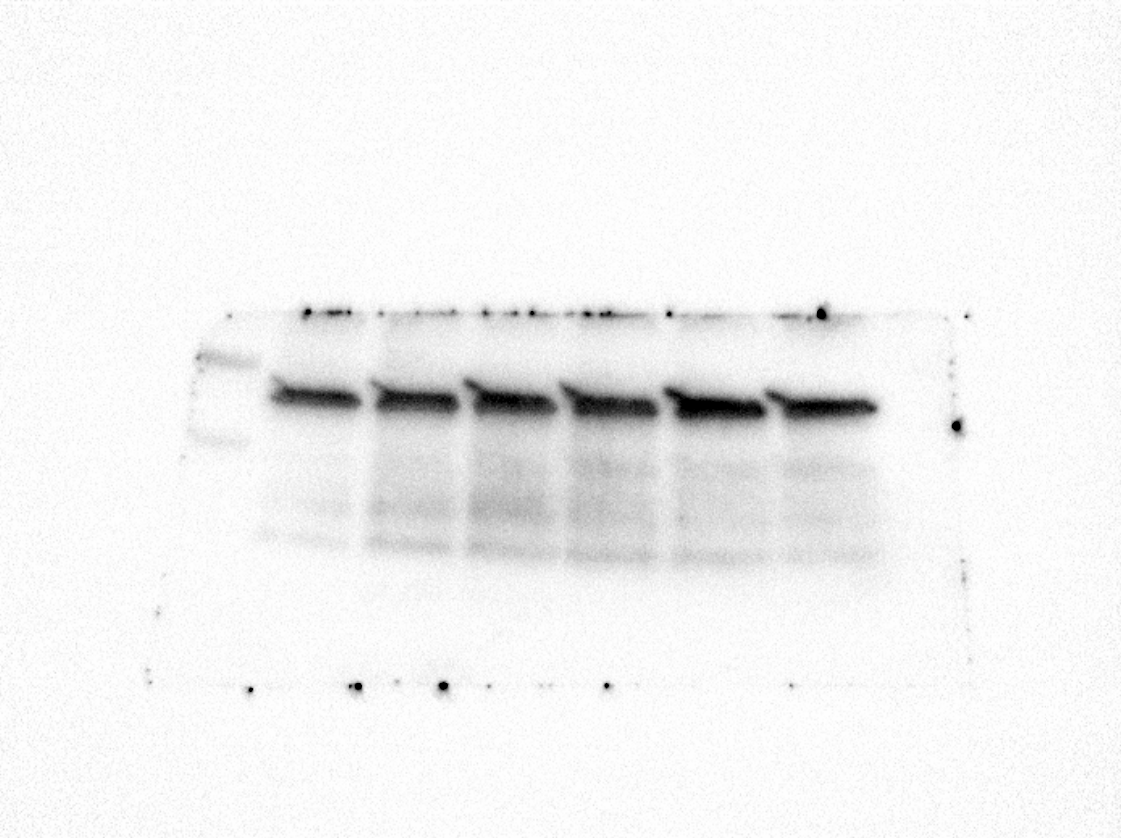

Supplement: Figure 7—source data 1. [file elife-98523-fig7-data1.zip › Figure 7/MEDAKA WT KO ACTIN (EZRIN).tif]

**Figure 7 b**

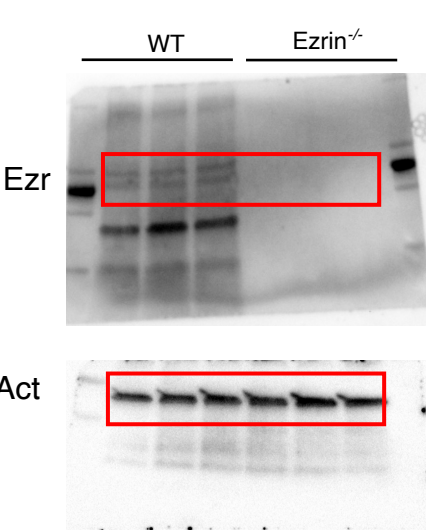

**Figure 7d**

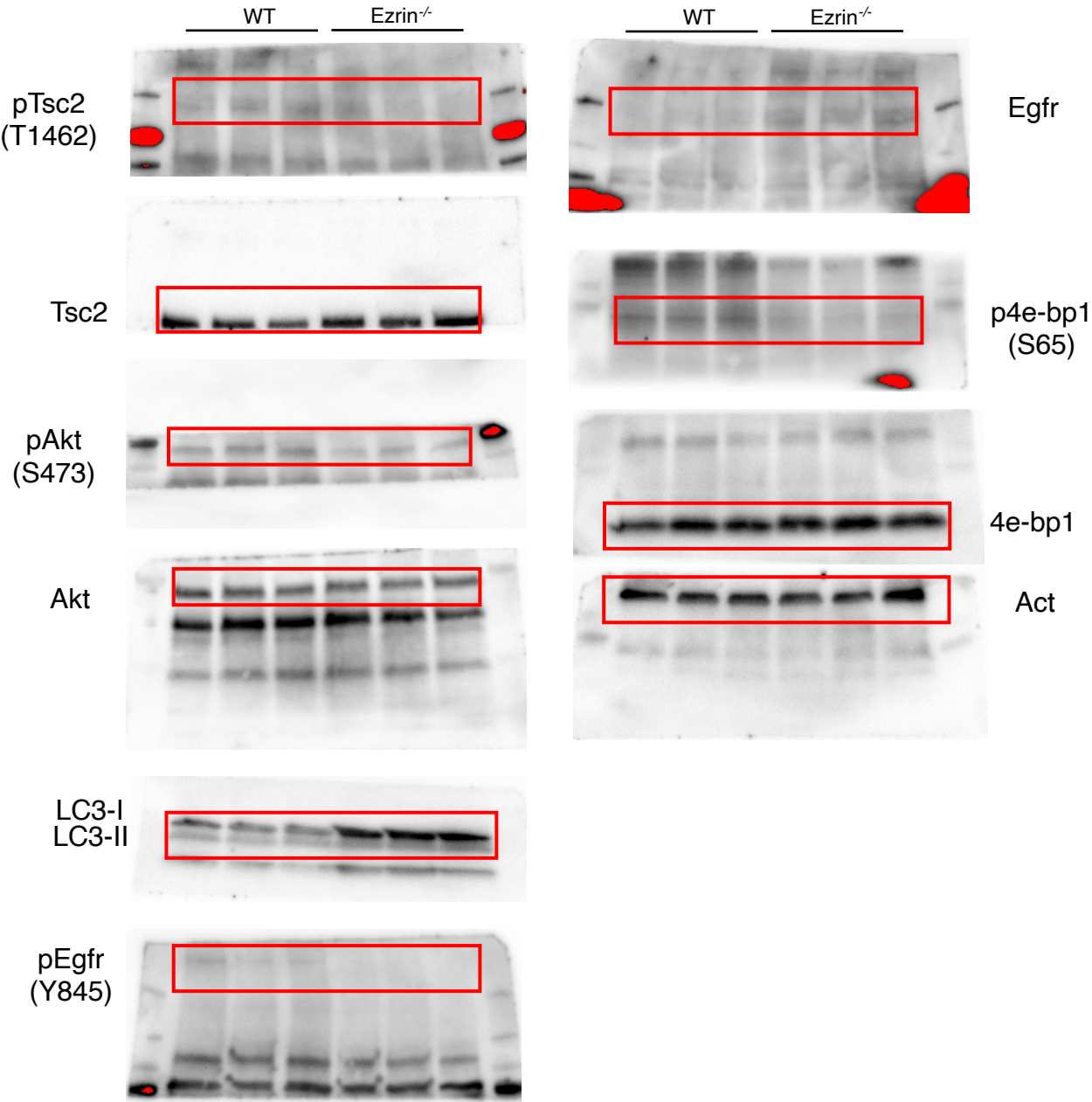

Supplement: Figure 7—source data 1. [file elife-98523-fig7-data1.zip › Figure 7/Figure 7.pdf]

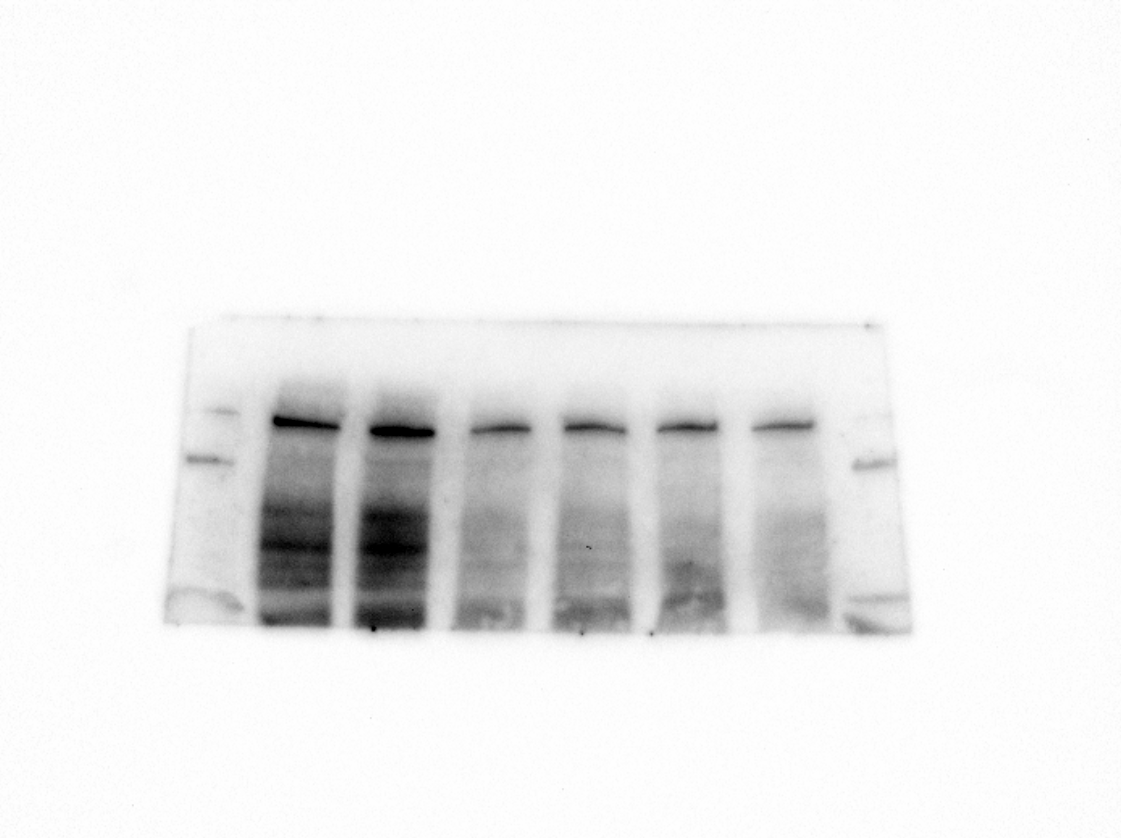

Supplement: Figure 7—figure supplement 1—source data 1. [file elife-98523-fig7-figsupp1-data1.zip › Figure suppl 6/MICE LIGHT DARK EGFR.tif]

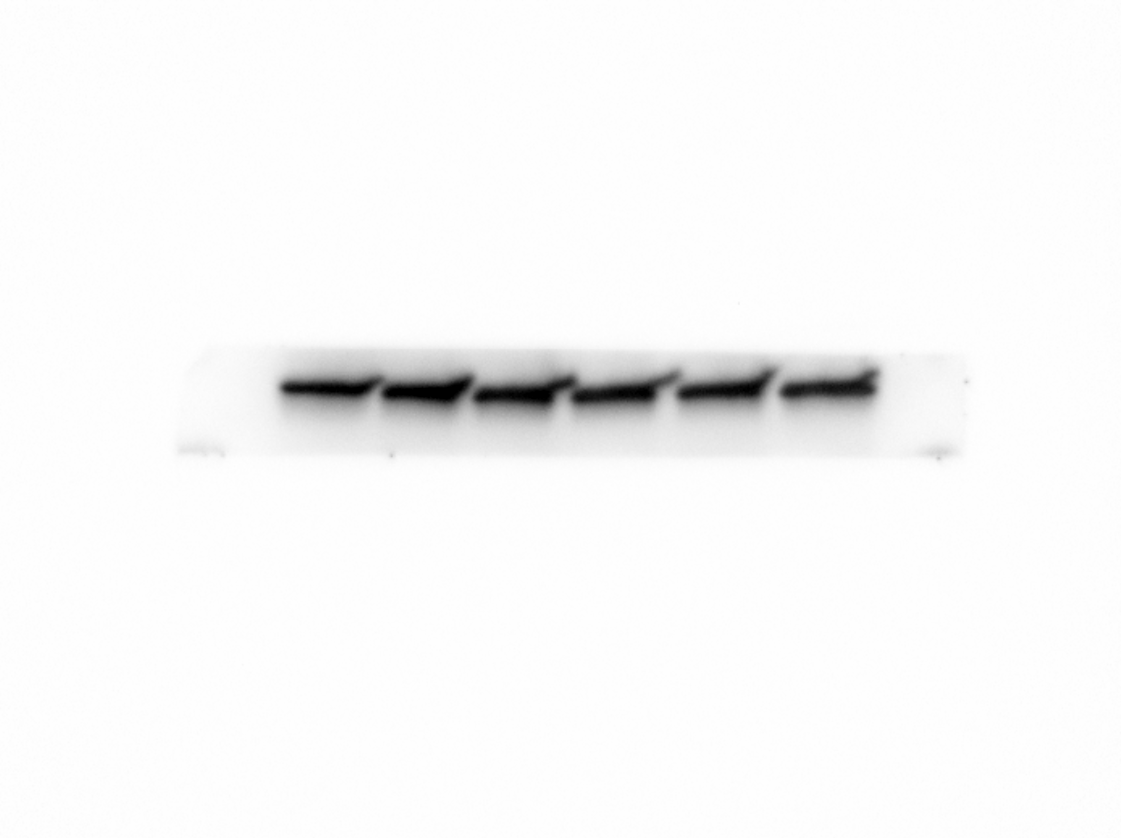

Supplement: Figure 7—figure supplement 1—source data 1. [file elife-98523-fig7-figsupp1-data1.zip › Figure suppl 6/MICE LIGHT DARK P38.tif]

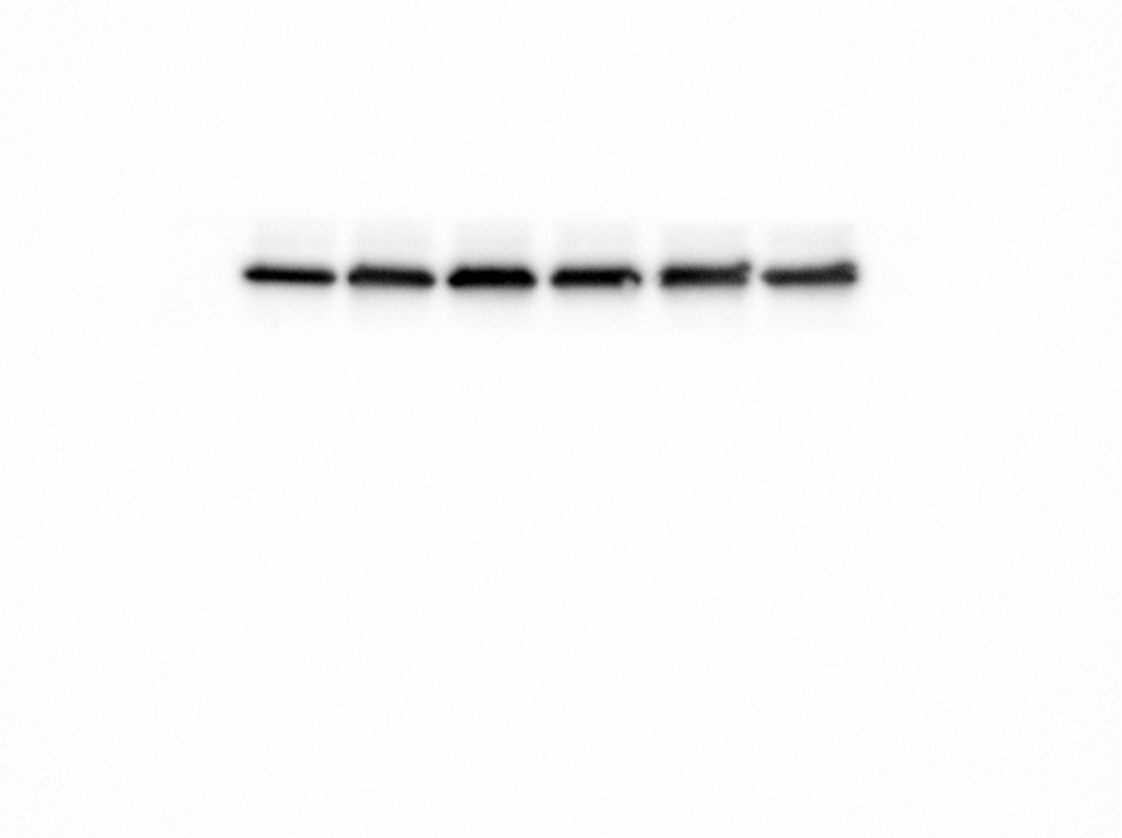

Supplement: Figure 7—figure supplement 1—source data 1. [file elife-98523-fig7-figsupp1-data1.zip › Figure suppl 6/MICE LIGHT DARK GAPDH.tif]

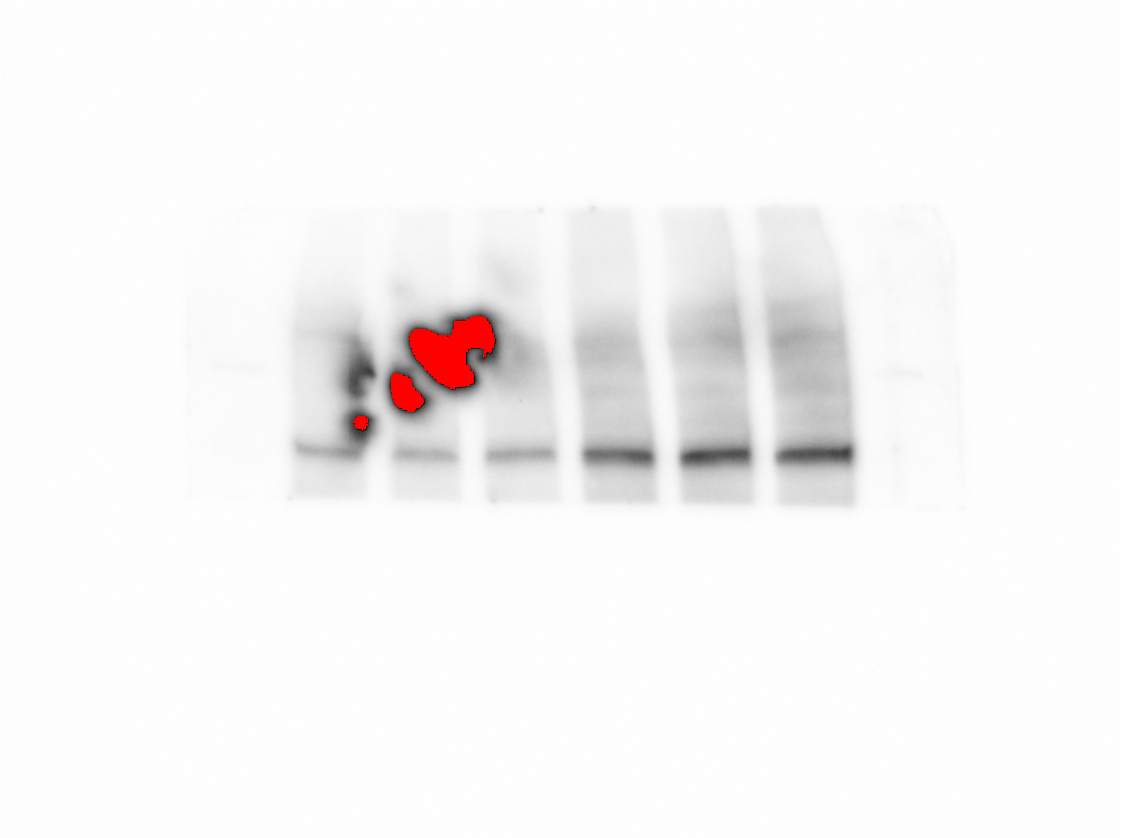

Supplement: Figure 7—figure supplement 1—source data 1. [file elife-98523-fig7-figsupp1-data1.zip › Figure suppl 6/MICE LIGHT DARK pTSC2.tif]

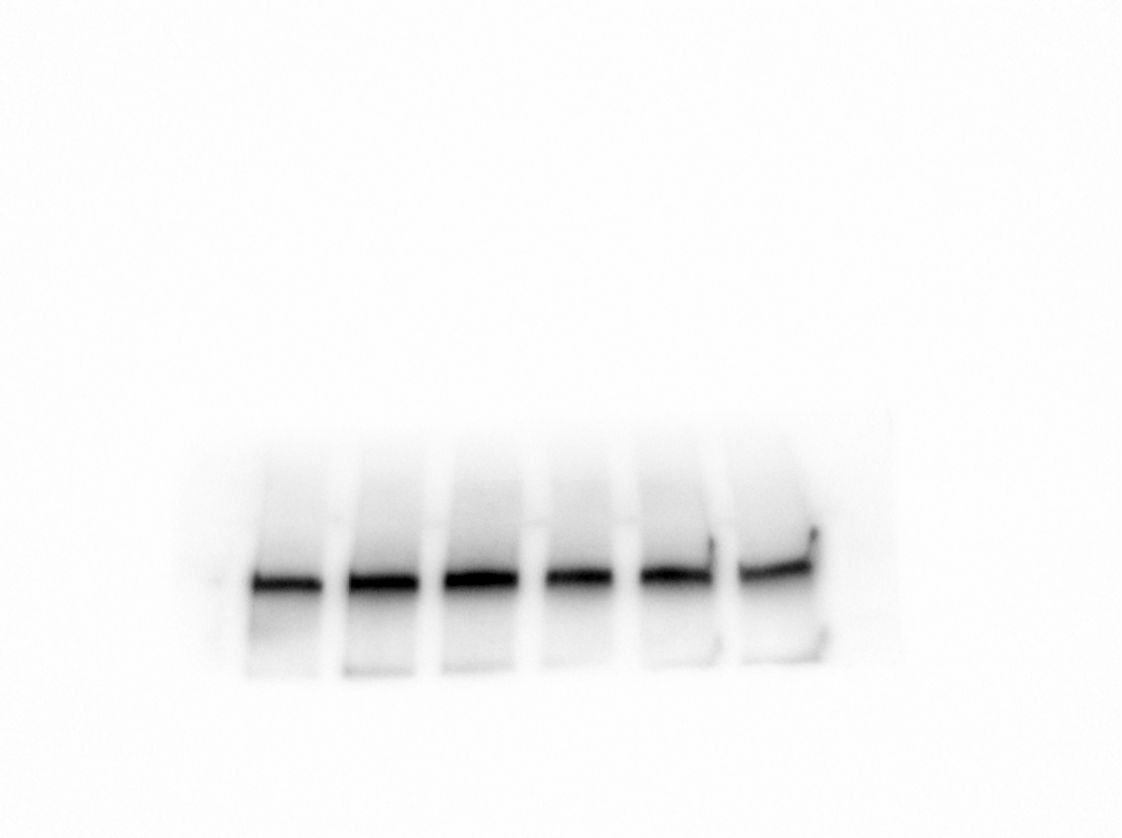

Supplement: Figure 7—figure supplement 1—source data 1. [file elife-98523-fig7-figsupp1-data1.zip › Figure suppl 6/MICE LIGHT DARK TSC2.tif]

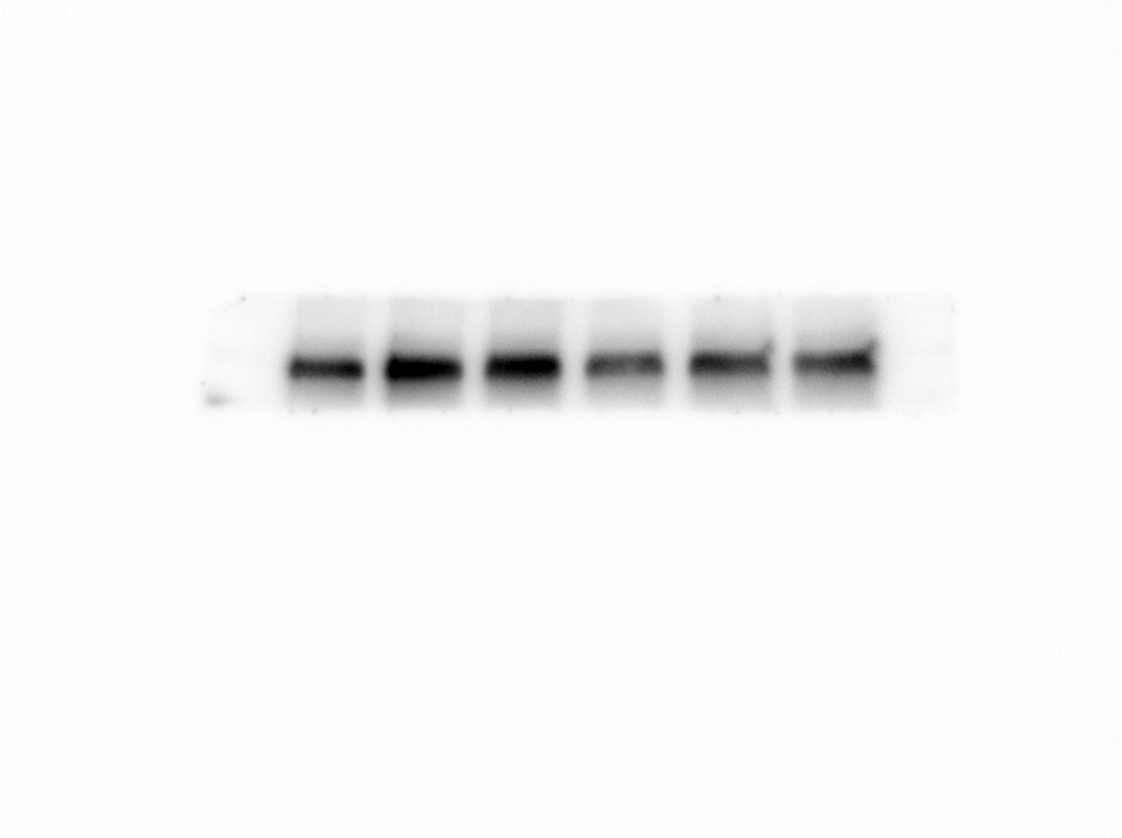

Supplement: Figure 7—figure supplement 1—source data 1. [file elife-98523-fig7-figsupp1-data1.zip › Figure suppl 6/MICE LIGHT DARK P70.tif]

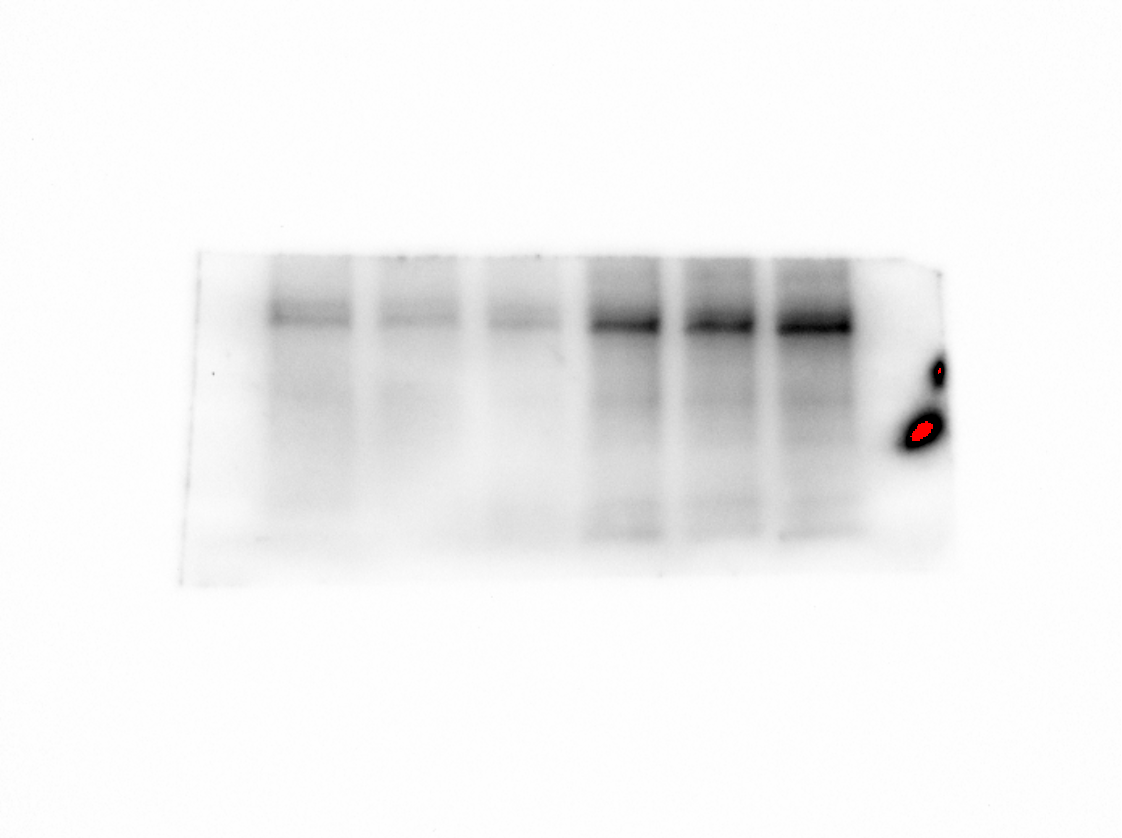

Supplement: Figure 7—figure supplement 1—source data 1. [file elife-98523-fig7-figsupp1-data1.zip › Figure suppl 6/MICE LIGHT DARK pP38.tif]

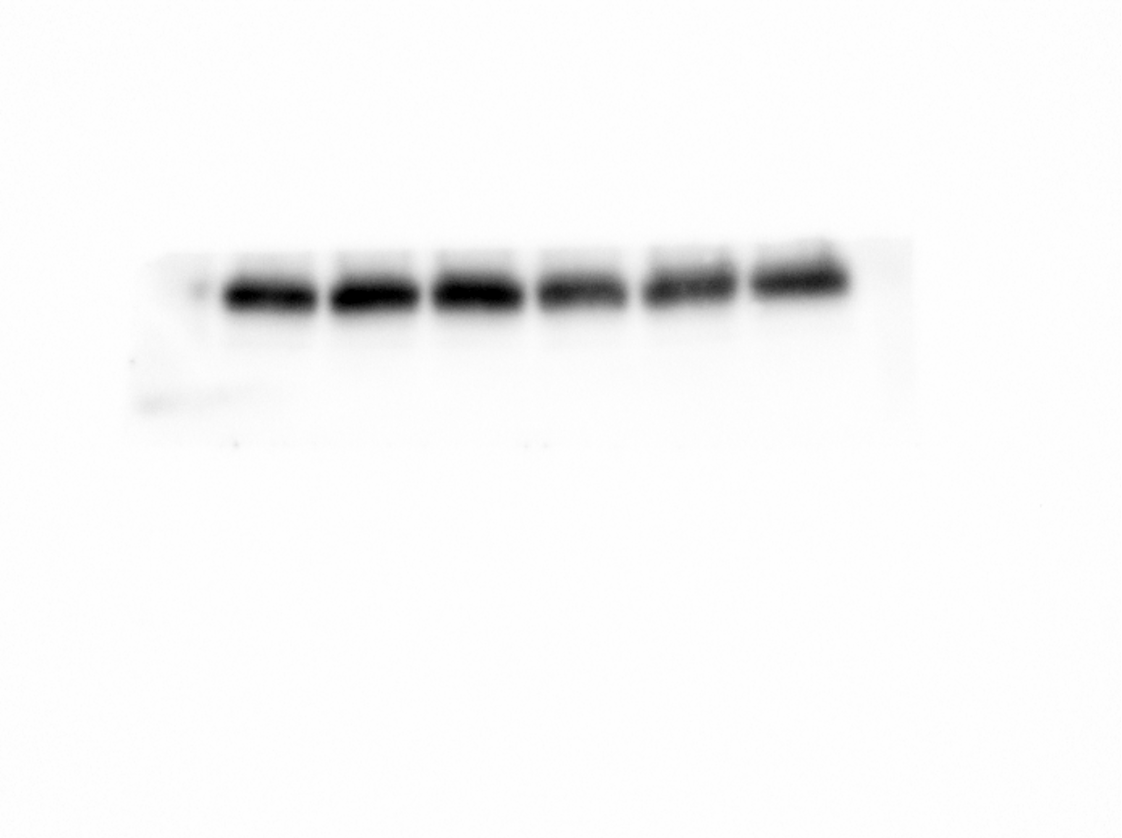

Supplement: Figure 7—figure supplement 1—source data 1. [file elife-98523-fig7-figsupp1-data1.zip › Figure suppl 6/MICE LIGHT DARK 4EBP.tif]

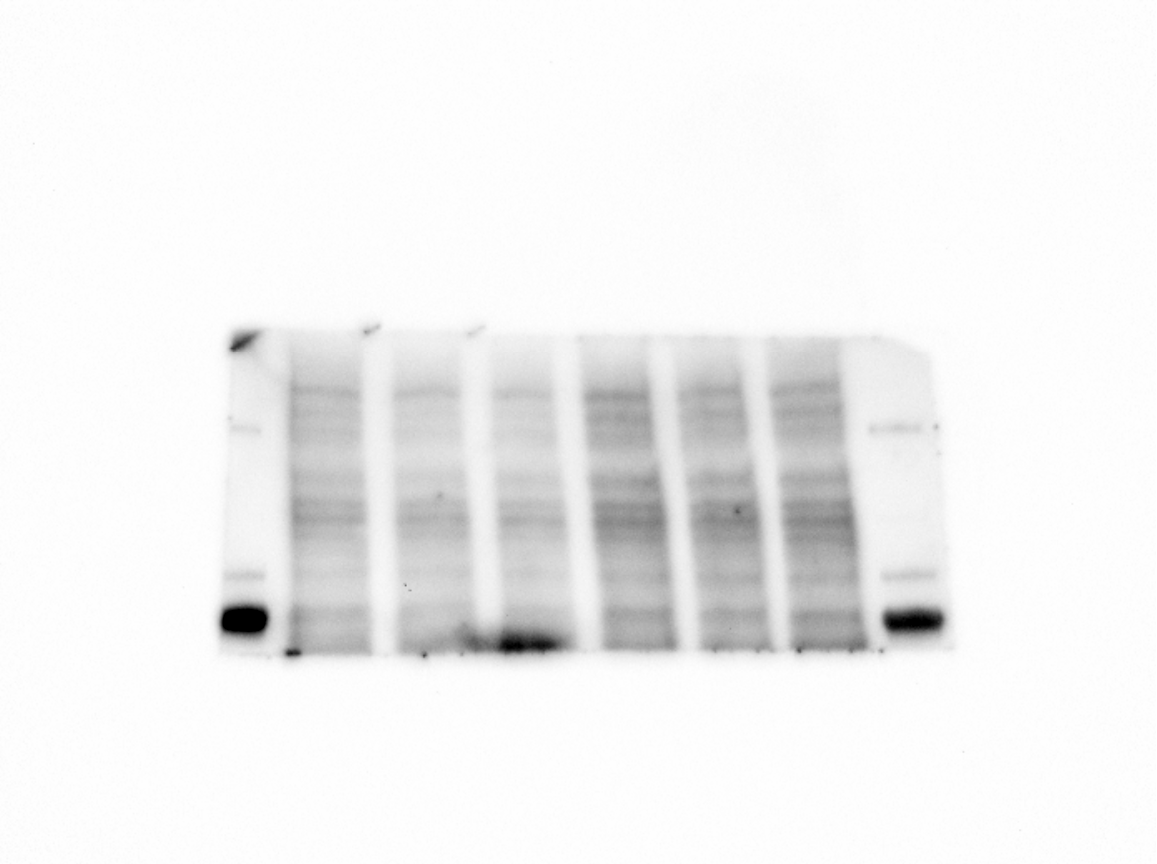

Supplement: Figure 7—figure supplement 1—source data 1. [file elife-98523-fig7-figsupp1-data1.zip › Figure suppl 6/MICE LIGHT DARK pEGFR.tif]

Figure Supplementary 6 b

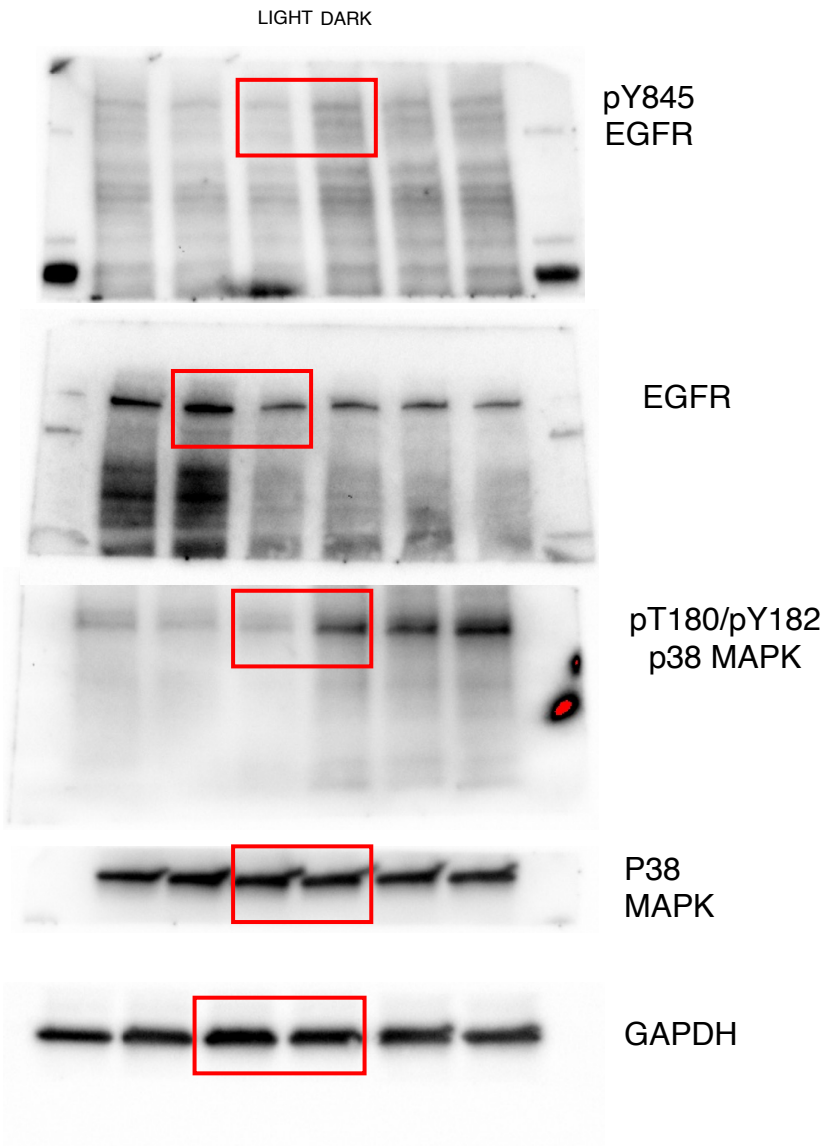

Figure Supplementary 6 c

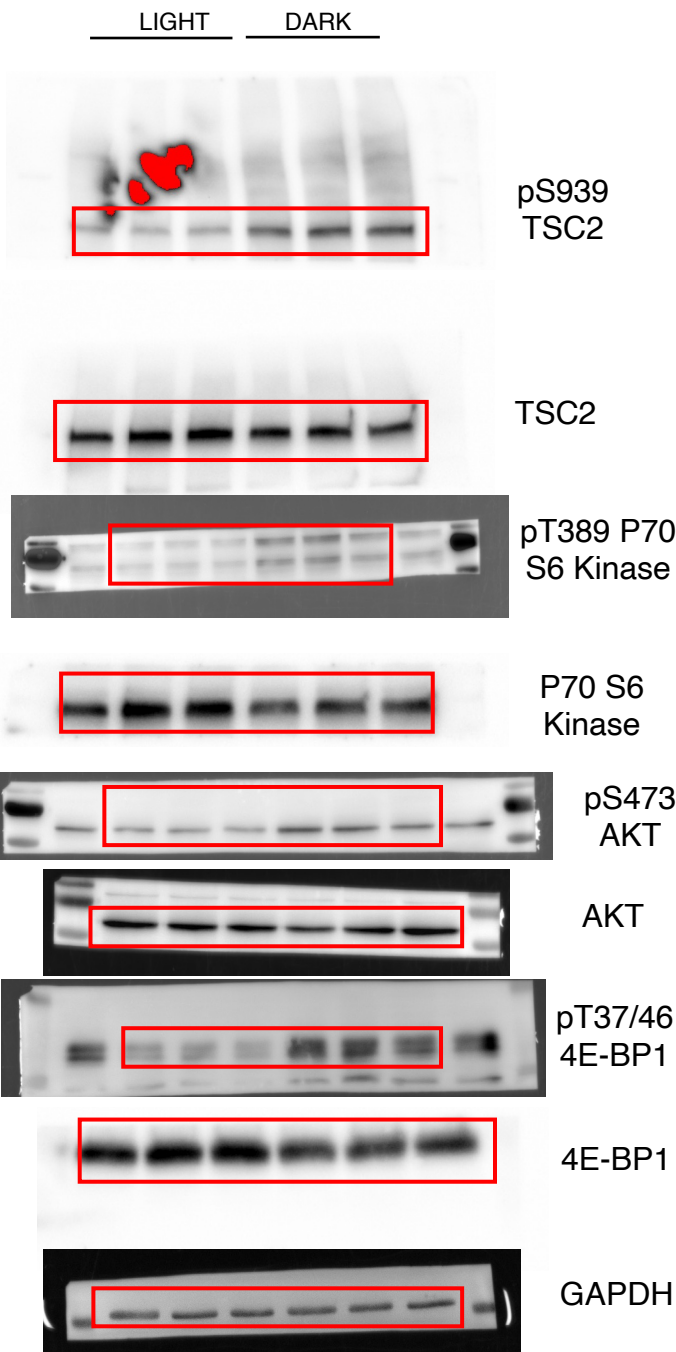

Supplement: Figure 7—figure supplement 1—source data 1. [file elife-98523-fig7-figsupp1-data1.zip › Figure suppl 6/Figure suppl. 6.pdf]
