# Supplementary material for: Ezrin defines TSC complex activation at endosomal compartments through EGFR–AKT signaling
Source: eLife. 2025 Feb 12;13:RP98523. doi: 10.7554/eLife.98523 (PMC11820125; doi:10.7554/eLife.98523)

Figure Supplementary 4 a

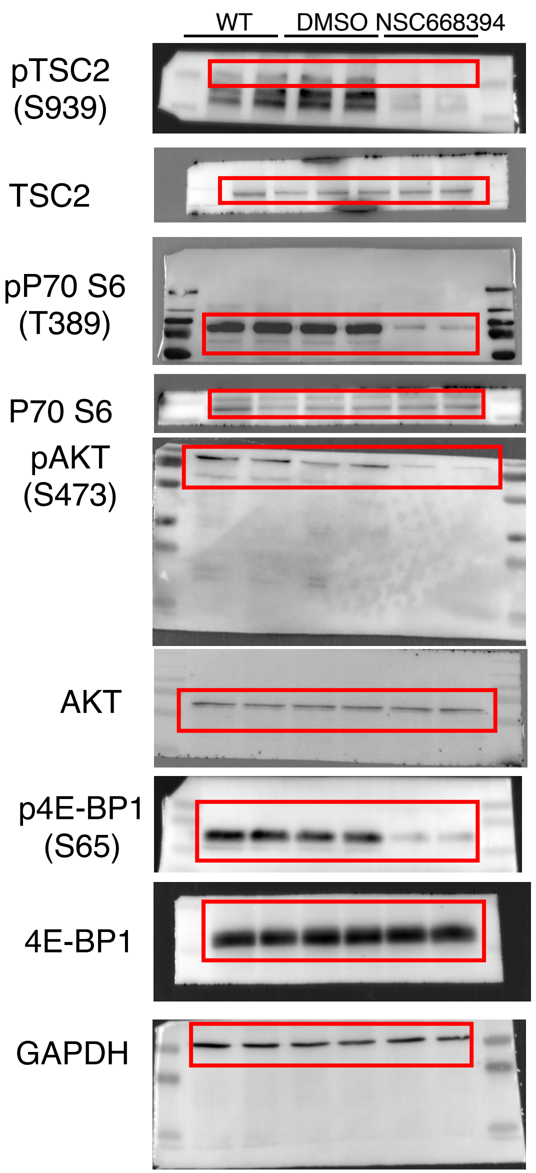

Figure Supplementary 4 b

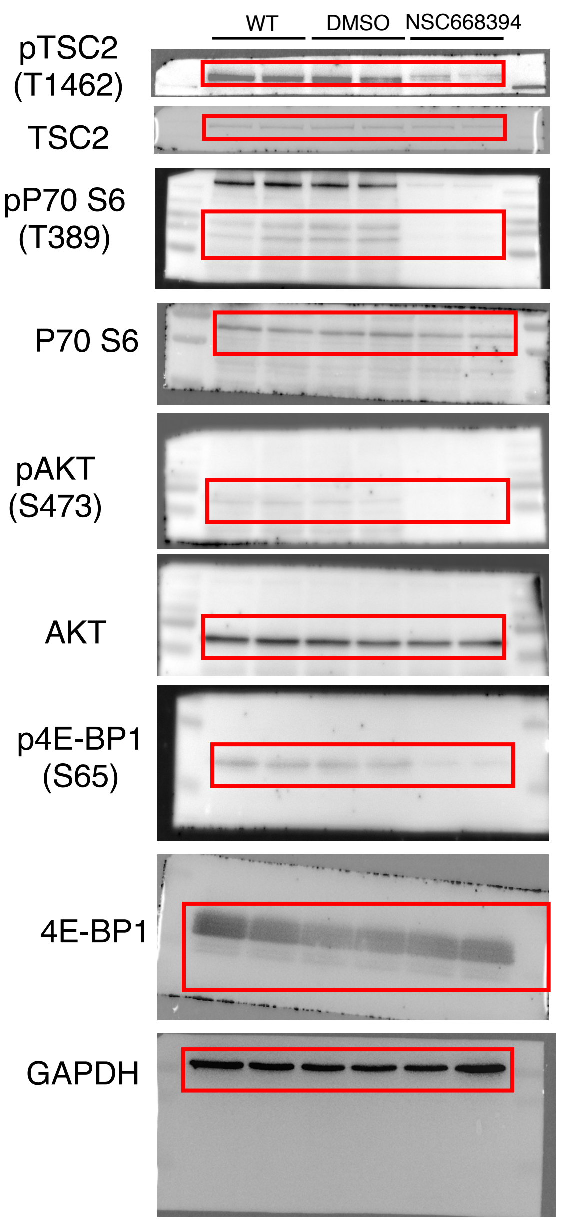

Figure Supplementary 4 d

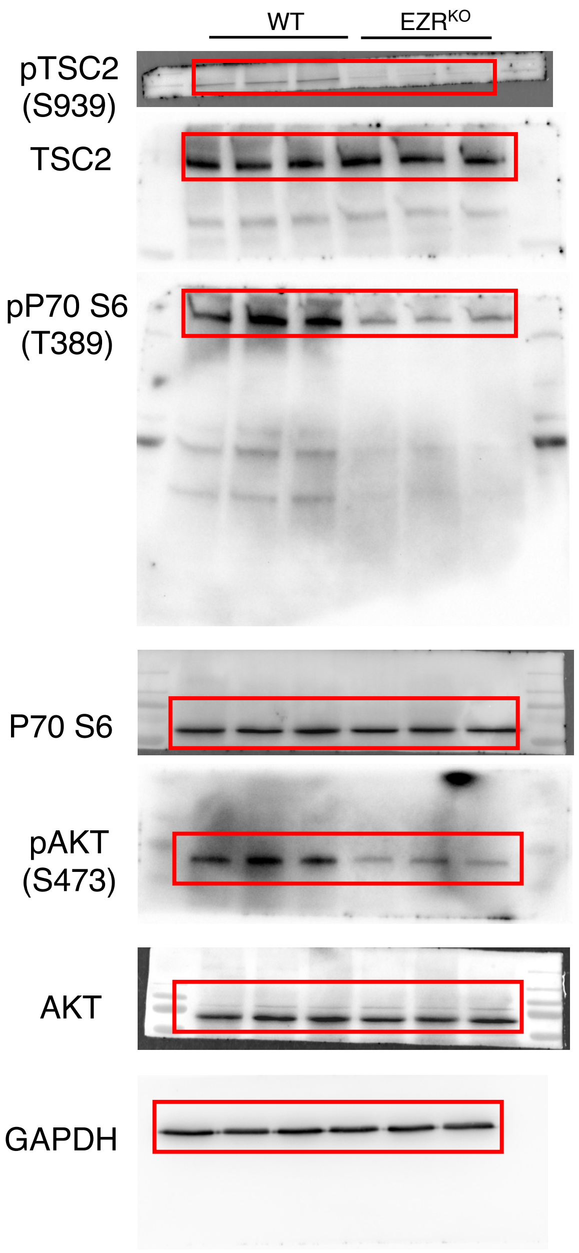

Figure Supplementary 4 f

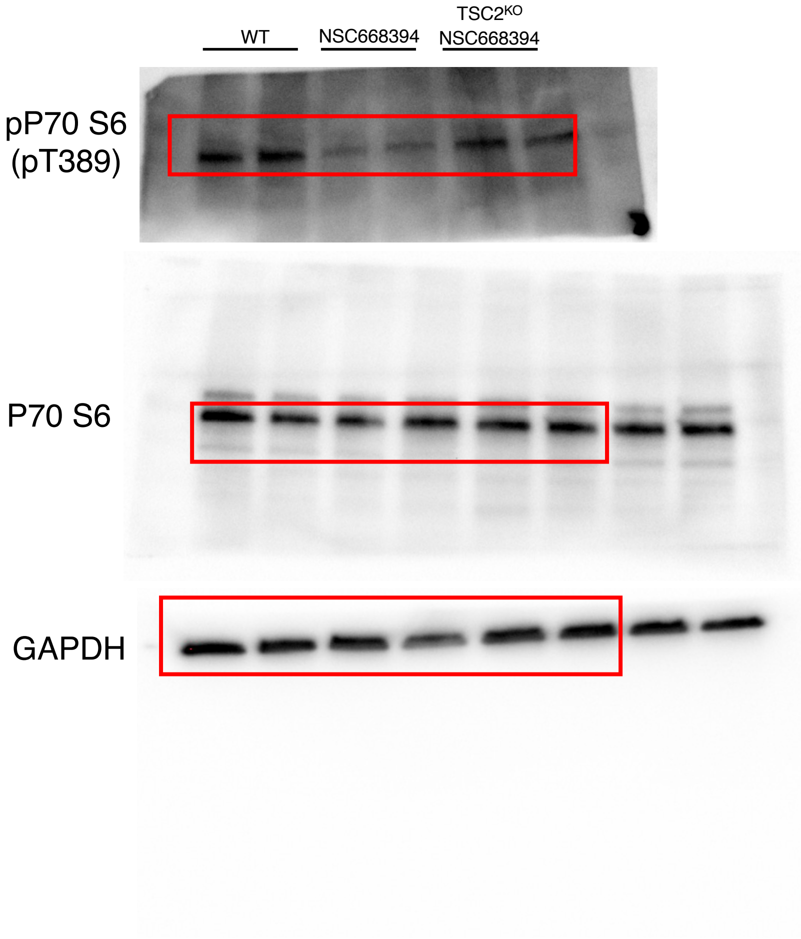

Supplement: Figure 6—figure supplement 1—source data 1. [file elife-98523-fig6-figsupp1-data1.zip › Figure suppl 4/Figure suppl. 4.pdf]
